# Enzymatic-related network of catalysis, polyamine, and tumors for acetylpolyamine oxidase: from calculation to experiment†

**DOI:** 10.1039/d3sc06037c

**Published:** 2023-12-29

**Authors:** Dong Fang, Zhiyang Zhang, Jihang Zhai, Baolin Guo, Pengfei Li, Xiaoyuan Liu, Jinshuai Song, Songqiang Xie, Ruibo Wu, Yuan Zhao, Chaojie Wang

**Affiliations:** a The Key Laboratory of Natural Medicine and Immuno-Engineering, Henan University Kaifeng 475000 P. R. China zhaoyuan@henu.edu.cn wcjsxq@henu.edu.cn; b School of Pharmacy, Henan University Kaifeng 475000 P. R. China; c School of Pharmaceutical Sciences, Sun Yat-sen University Guangzhou 510006 P. R. China; d Department of Chemistry and Biochemistry, Loyola University Chicago Chicago Illinois 60660 USA; e Green Catalysis Center, College of Chemistry, Zhengzhou University Zhengzhou 450001 P. R. China

## Abstract

The regulation of enzymes and development of polyamine analogs capable of controlling the dynamics of endogenous polyamines to achieve anti-tumor effects is one of the biggest challenges in polyamine research. However, the root of the problem remains unsolved. This study represents a significant milestone as it unveils, for the first time, the comprehensive catalytic map of acetylpolyamine oxidase that includes chemical transformation and product release kinetics, by utilizing multiscale simulations with over six million dynamical snapshots. The transportation of acetylspermine is strongly exothermic, and high binding affinity of enzyme and reactant is observed. The transfer of hydride from polyamine to FAD is the rate-limiting step, *via* an H-shift coupled electron transfer mechanism. The two products are released in a detour stepwise mechanism, which also impacts the enzymatic efficiency. Inspired by these mechanistic insights into enzymatic catalysis, we propose a novel strategy that regulates the polyamine level and catalytic progress through the action of His64. Directly suppressing APAO by mutating His64 further inhibited growth and migration of tumor cells and tumor tissue *in vitro* and *in vivo*. Therefore, the network connecting microcosmic and macroscopic scales opens up new avenues for designing polyamine compounds and conducting anti-tumor research in the future.

## Abstract 2

Enzymatic-related network of catalysis, polyamine, and tumors for acetylpolyamine oxidase: from calculation to experiment.

## Graphical abstract

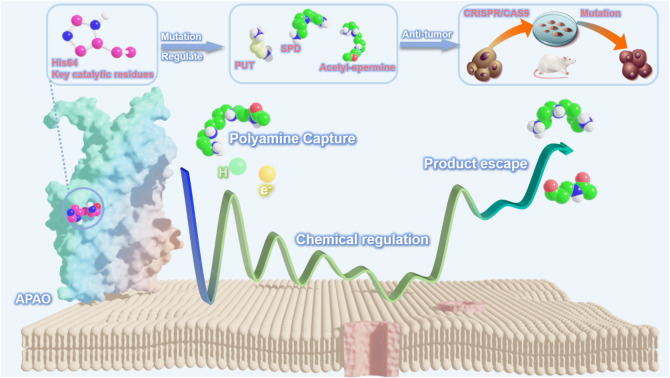

## Introduction

**Fig. 1:**
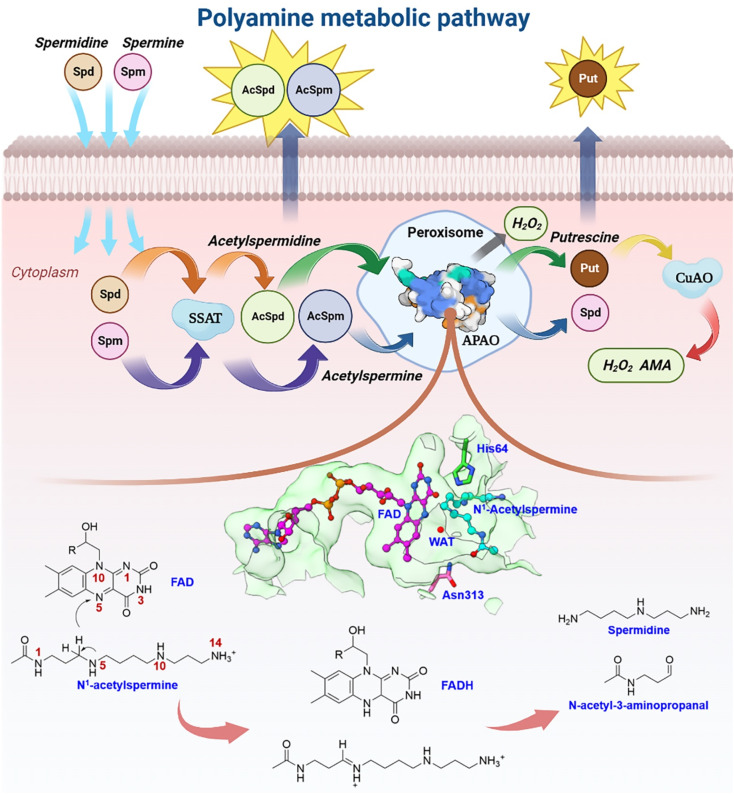
Plausible polyamine metabolic pathway and chemical catalytic mechanism.

Natural polyamines (PAs), including spermine (SPM), spermidine (SPD), and putrescine (PUT), are ubiquitous, positively charged organic amine compounds. They interact with biomolecules, such as DNA, RNA, and proteins, attending gene expression, cell proliferation, and signal transduction, which are related to disease and cancer.^1–8^ The malignant proliferation of tumor cells is highly associated with the polyamine level.^3,9–11^ Animal cells possess a set of mechanisms that regulate the catabolism of polyamines to maintain an optimal level of polyamine concentration.^10,12,13^ As shown in Fig. 1, SPM and SPD are converted into acetyl-spermine (AcSpm) and acetyl-spermidine (AcSpd), respectively, by *N*^1^-spermine/spermine *N*^1^-acetyltransferase (SSAT). Some acetyl polyamines are excreted by specific transport carriers on the cell membrane surface, while others are oxidized to SPD and PUT by acetylpolyamine oxidase (APAO). PUT is then oxidized to aminoaldehyde (AMA), ammonium ions, and H_2_O_2_ by Cu-containing amine oxidase (CuAO), upon which some of the AMA and H_2_O_2_ induces cell apoptosis and ammonium ion transportation *in vitro*. The other AMAs are converted into gamma-aminobutyric acid (GABA), which is used in cellular respiration. Furthermore, SPM can undergo oxidation to form SPD through spermine oxidase (SMO) without acetylation. This process generates AMA and H_2_O_2_, which can trigger apoptosis.

Our laboratory group has focused on investigating polyamine for about 20 years,^14–17^ and one of the biggest challenges we have encountered is related to the regulation of enzymes and design of polyamine analogs that can control the dynamics of endogenous polyamines to elicit anti-tumor effects. Investigating a catalytically-related network with multiple dimensions may be a good route to tackle the root of problems, which remains unsolved. Herein, we mainly focus on APAO, an important enzyme in maintaining polyamine homeostasis.^13,18–20^ It is a flavin adenine nucleotide (FAD)-dependent amine oxidase. Sjögren *et al.*^18^ identified the crystal structures of murine *N*1-acetylpolyamine oxidase in oxidized (APAO_ox_, where the FAD factor is half-reduced) and reduced (APAO_red_, where the FAD factor is oxidized) forms. They postulated that the chemical catalysis may involve hydride transfer and attendant water based on the structures (see Fig. 1). Asn313 was revealed to affect the binding affinity but not to participate in catalysis, and thus the residues involved in the chemical step remain unclear. A study by Fitzpatrick^20^ revealed that His64 mutation decreased the *k*_cat_ value ∼15-fold when *N*^1^-acetylspermine was the polyamine substrate, but the work did not reveal the detailed enzymatic mechanism.

Until now, details of how the enzyme works have remained a mystery. Intriguingly, it would directly affect research on polyamine homeostasis and anti-tumor. Currently, the design of polyamine derivatives is also limited. Herein, we first model the panorama of enzymatic catalysis using ∼6.2 μs classical molecular dynamics (MD) and ∼2.7 ns quantum mechanics (density functional theory)/molecular mechanics molecular dynamics (QM/(DFT)/MM MD) to study the chemical transformation, participant residues, unidentified intermediate and transition states, polyamine capture, and product release at electronic and atomic levels. Inspired by the calculations, we experimentally replace the wild-type APAO with a mutant system in murine breast cancer cells and tumor tissues to determine the role of key residues in regulating polyamine catabolism and tumor progression. By integrating insights from the realms of electron, atom, molecule, protein, cell, and tumor, we have successfully constructed a catalytic network that bridges the micro and macro scales. Within this network, we have identified a potential target site for antineoplaston therapy, offering novel perspectives for inhibitor design, polyamine regulation, and anti-tumor therapeutic strategies. This groundbreaking approach opens up new avenues of exploration in these areas, holding significant promise for advancing the field of cancer treatment.

## Results and discussion

### Strong ion-pair and hydrogen bond interactions facilitate the recognition and transportation of reactants by the APAO_red_·FAD complex

**Fig. 2:**
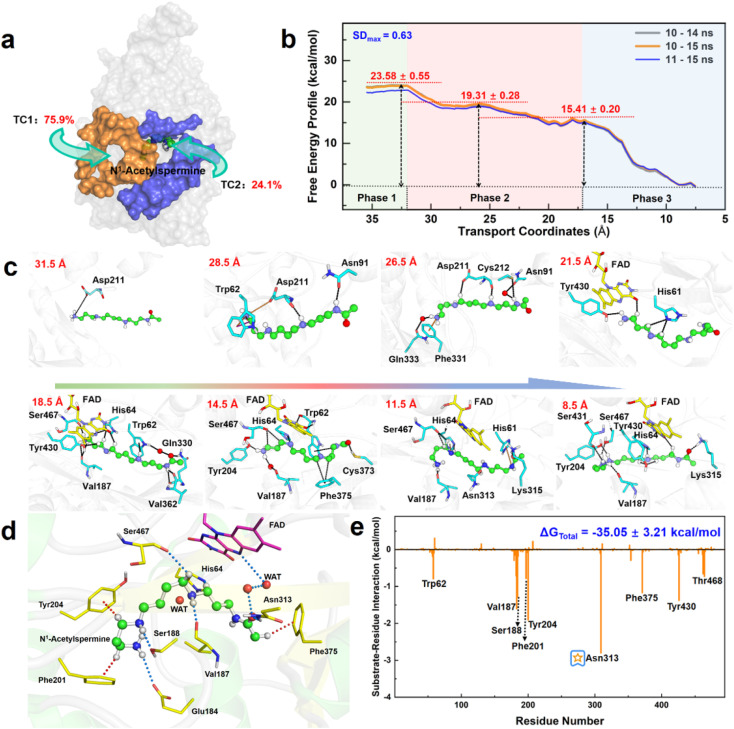
Capture, delivery, and binding of *N*^1^-acetylspermine by APAO. (a) Channels for *N*^1^-acetylspermine entering into APAO. (b) Free energy profile for the transportation of the reactant from outside the APAO into the active site. (c) Key snapshots for initial recognition and transportation. (d) Static binding mode for the reactant and FAD in the active site of APAO. (e) Contribution of all residues obtained by free energy decomposition.

Herein, the reactant *N*^1^-acetylspermine is transported through two possible channels (Fig. 2a). The most favourable channel is located between residues of Glu84–Pro103, Ser365–Ser367, and Pro327–Gln330 and has the dominant share of trajectories (∼76%) (Table S1†). Three stages are identified based on the combination of conformational changes in protein or ligand and variation tendency of energy (Fig. 2b, c, Sections S1.1, S1–S2†).

In the first phase (TC1 > 32 Å), the reactant hovers at the protein entrance and is exposed to the water environment without any binding force from the residues. Moreover, little fluctuation is observed in the free energy profile.

In the second stage (16.5 Å < TC1 ≤ 32 Å), the details of how specific residues participate in the whole process are listed in Section S2 and Fig. S3,† and the following three key issues should be noted. First, Asp211 is crucial in facilitating substrate initial recognition at the entrance pocket since its carboxyl group forms a strong ion-pair interaction with the positive amino group of the reactant. The interaction probably counteracts the desolvation of the acetylspermine, and no barrier can be observed. Second, between 32 and 22 Å, the probability of observing Asp211 is close to 100%, and the hydrogen bond interaction contributes most to the movement of *N*^1^-acetylspermine. In addition, the cation–π interactions arising from Trp62 and Phe331 are useful for driving the process. Third, cofactor FAD and more aromatic residues participate in the *N*^1^-acetylspermine transport process through hydrogen bond and cation–π interactions in the latter half-period between 22 and 16.5 Å. Furthermore, Val187 exhibits a high probability of occurrence, making it a key player in driving and stabilizing the reactant. Regarding energy changes, the whole second stage gently decreases by ∼8.2 kcal mol^−1^, which lasts for the longest time during the entire transportation. The newly formed hydrogen bond and conjugate interactions are potentially responsible for exothermic reactions in this process.

In the final stage (7.5 Å ≤ TC1 ≤ 16.5 Å), the reactant relaxes until it gradually reaches a stable state in the active site. Val187 and Ser467 have 80% and 90% probabilities of occurring, respectively, both of which enhance the capacity of the substrate to access the catalytic site. Finally, the *N*^1^-acetylspermine is bound to a catalytically active site, such as Val187, Tyr204, Asn313, or Ser467, by hydrogen bond interactions. The observed rapid decrease in free energy by approximately 15.4 kcal mol^−1^ can be primarily attributed to the formation of a hydrogen bond network within the catalytic site.

The recognition and transportation of the reactant are strongly exothermic, which means that acetylspermine has high binding affinity for APAO. Then, the enzyme-reactant static complex relaxes to reach equilibrium after MM MD simulations (Fig. S4†). As shown in Fig. 2d, the acetyl group at one end of the substrate forms a hydrogen bond interaction with the side chain of Asn313. The protonated amino group of the substrate forms an ion-pair interaction with the carbonyl group on the side chain of Glu184. In addition, Ser188 and Val187 contribute to the localization of AcSpm by hydrogen bond interactions. Tyr204, Phe201, and Phe375 provide conjugated rings to form σ–π hyperconjugation with *N*^1^-acetylspermine. Between FAD and *N*^1^-acetylspermine, the water-bridge hydrogen bond is also useful for stabilizing the reactant. The total binding free energy of the complex is ∼−35.1 kcal mol^−1^, and the van der Waals (Δ*G*_vdW_), electrostatic (Δ*G*_ele_), and non-polar solvent (Δ*G*_SA_) interactions offer a positive driving force for substrate binding, while the solvent energy (Δ*G*_GB_) is disadvantageous (Table S2†). Understanding how to reduce the effect of solvent energy may be useful in designing inhibitors with better binding affinity. In this study, Glu184, Val187, Ser188, Phe201, Tyr204, Asn313, and Phe375, are identified to increase substrate stabilization from an energy perspective (Fig. 2e). Among the amino acids examined, Asn313 plays a prominent role in driving the system due to its crucial involvement in polar interactions. These interactions encompass electrostatic forces and solvation energy, as represented in Fig. S5.† Notably, the stability of this interaction is further confirmed through a comprehensive hydrogen bond analysis (see Fig. S6†). This explains the findings by Sjögren *et al.*^18^ that Asn313 mutants increased *K*_s_ in the experiment. Moreover, the experimental group discovered that Asn313 mutants did not affect *k*_cat_, indicating that the residue was not involved in this chemical reaction. We note that the His64 is adjacent to the possible reacted water and *N*^1^-acetylspermine, and thus it may serve as a “proton-shuffler” to participate in chemical catalysis.

### Water and His64 assist in the transfer of a hydride to form APAO_ox_·FADH^−^·hydroxylated polyamine

**Fig. 3:**
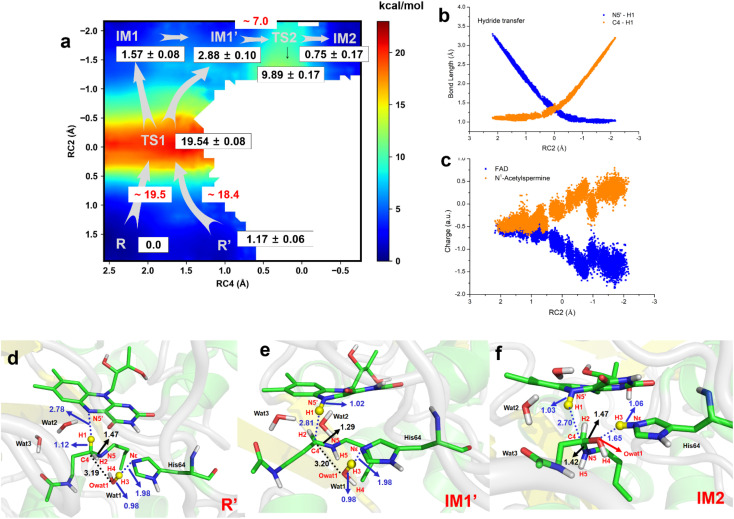
Generation of FADH- and hydroxylated polyamine. (a) Two-dimensional free energy profile for generation of FADH- and hydroxylated intermediate calculated on the basis of QM/MM MD simulations (RC2 = dN5′-H1 – dC4-H1, RC4 = dNε-H3 – dH3-Owat1). (b) Changes in bond length for N5′-H1 and C4–H1 in the hydride transfer process. (c) Changes in charge of FAD and *N*^1^-acetysperine in the QM region in the hydride transfer process. (d) The most favorable reactant of R′ for the chemical reaction is used. (e) The intermediate state of IM1′ after complete hydride transfer. (f) Hydroxylated intermediate after water attacks.

The two-dimensional free energy profile (Fig. 3a, and Sections S1.2, S3, S7–S13†) shows that before hydride transfer, Wat1 floats between *N*^1^-acetylspermine and His64. It likes to target and attack the C4 position, leading to the identification of two distinct equilibrium states, referred to as R and R′. Fig. 3a and S13† show that R′ has higher energy than R with a difference of ∼1.2 kcal mol^−1^, and a small barrier of ∼0.7 kcal mol^−1^ is observed. Hence, it is more like a reactant platform. The distances of H1–N5′, H3-Nε and C4-Owat1 are shorter in R′ than in the R state, and thus R′ is beneficial for the reaction occurring from energetic and conformational perspectives (Fig. 3d).

Initially, H1 undergoes a transfer process, moving from the C4 position of *N*^1^-acetylspermine to the N5′ position of FAD. During this transfer, the C4–H1 bond lengthens, while the H1–N5′ bond shortens. Additionally, the bond length between C4 and N5 is shortened, resulting in the manifestation of double-bond characteristics. These changes are attributed to the negative charge transfer from C4 to N5′, accompanied by shifts in the electron cloud from N5 to C4. These transformations are visually represented in Fig. 3d–e and S13.† The complete transfer of H1 breaks the original hydrogen bond network between the two substrates, which causes fluctuation of the water-bridge. Hence, two main forms of IM1 and IM1′ are identified as the first intermediates (Fig. S13†). The FAD and polyamine in the two forms exhibit a similar conformation, whereas the location of water molecules is different. The route of R → TS1 → IM1 is shorter than R → TS1 → IM1′, while Wat1 in IM1′ is more likely to attack C4 by delivering the proton to His64 because of the shorter distance of Owat1-C4 (Fig. 3e).

The hydride transfer involves electron and hydrogen transfer, and thus the transfer mechanism is studied. As listed in Fig. 3b and c, when RC2 changes from ∼2.1 to ∼ −0.8 Å, H1 approaches the N5′ of FAD. Synchronously, the charge of FAD changes from ∼−0.5 to ∼−1.5 a.u. accompanied by charge changes of *N*^1^-acetylspermine from ∼−0.5 to ∼0.5 a.u. Moreover, localizing RC2 from ∼−0.8 to ∼−2.2 Å indicates the end of H1 transfer, and the charge of FAD and *N*^1^-acetylspermine fluctuates. Accordingly, the hydride transfer process follows an H-shift coupled electron transfer mechanism.

After hydride transfer, the water molecules attack C4 with proton capture by His64 (see Fig. 3e–f and S13–S14†). The C4–N5 bond is lengthened from 1.29 ± 0.02 to 1.42 ± 0.03 Å, which may be caused by the shift of the electronic cloud from Owat1 to C4 that offsets the electron deficiency of C4. The neutral state of His64 changes to a proton state, and C4 and Owat1 form a single bond to generate a hydrolyzed intermediate (see Fig. 3f).

If the reaction follows the R′ → TS1 → IM1 (or IM1′) route, it requires ∼18.4 kcal mol^−1^ (see Fig. 3a). During the transition from IM1 (or IM1′) to IM2, there is a clear reduction in the energy barrier. As a result, the ability of the water molecule to attack C4 and donate a proton to His64 becomes significantly more favorable compared to the hydride transfer process. Besides, the barrier to the enzymatic process is 15.6 kcal mol^−1^, which is estimated from experimental data (*k*_cat_ = 27 ± 2 s^−1^, measured in air-saturated buffers at pH 9) using transition-state theory. Our calculated barrier is overestimated, and the tunneling effect is evaluated. The Wigner tunneling correction factor^21^ is 1.24–1.74 based on three levels, and the corresponding decreasing active energy barrier is 0.37–0.56 kcal mol^−1^. Hence, the tunneling effect is not significant (Section S4, Table S3,† and Appendix). Besides, the results of B3LYP and M06-2X agree with each other, and the maximum error of three levels for the energy barrier is 1.10 kcal mol^−1^ (see Section S4, Fig. S15, Table S3,† and Appendix). In previous studies, Hammes-Schiffer *et al.*^22^ mentioned that the dominant distance facilitating the PCET reaction is smaller than the equilibrium donor–acceptor distance. Vibrational motion plays a vital role in changing the donor–acceptor distance. Brooks III *et al.*^23^ suggested that conformational switching strongly affects the barrier for hydride transfer in DHFR. The reactant here is a long-chain polyamine that is very flexible with various conformations. Only one or two may have its limitations; this is probably the main reason for overestimation of the barrier. More studies are needed to explore the influence of different initial conformations. In addition, the tested one-dimensional sampling shows that two dimensions may reduce the error caused by the selection of reaction coordinates (Sections S1.3, S5, and Fig. S16–S20†).

### Successive proton shuffling leads to the formation of various protonated products

For the oxidization of *N*^1^-acetylspermine into spermidine and *N*-acetyl-3-aminopropanal, the C4–N5 bond should be broken, and the newly formed hydroxyl should be deprotonated. The direct way is for the amino group to attract the proton in the hydroxyl group with the breaking of the C4–N5 bond to form aldehyde and spermidine with a neutral amino group in N5. Apart from that, there is another route. When His64 is protonated, it provides the proton for the amino group, and the proton in the hydroxyl group transfers to His64 or the amino group to mediate the cleavage of *N*^1^-acetylspermine. If the reaction follows this mechanism, the neutral and protonated amino groups in the N5 of spermidine may be generated (see Fig. S21†). The mechanism and thermodynamics of both routes were studied (see Sections S1.4–S1.5 and Fig. S22–S26†).

If the proton donor is the hydroxyl group in the substrate, a stepwise mechanism is observed (Fig. S24†). IM2 to IM3a is a “two-proton-transfer” process. H4 completes the delivery from hydroxyl to N5. Meanwhile, the Nε of His64 delivers H3 to Owat1 to re-form the hydroxyl group, and the intermediate with an –NH_2_^+^– group is generated. The C4–N5 bond changes from 1.42 ± 0.03 to 1.55 ± 0.03 Å without breaking. From IM3a to the final products, His64 recaptures H3 and becomes protonated during the lengthening and breaking of C4–N5. Thus, the final products of *N*-acetyl-3-aminopropanal and spermidine with one terminal protonated are generated. This route follows a “self-deprotonation-reprotonation” mechanism, and His64 is responsible for mediating proton transfer, which overcomes two barriers of ∼23.9 and ∼9.4 kcal mol^−1^. The first barrier is much higher than that found in the experimental data, and thus may be not the favorable route for product generation.

**Fig. 4:**
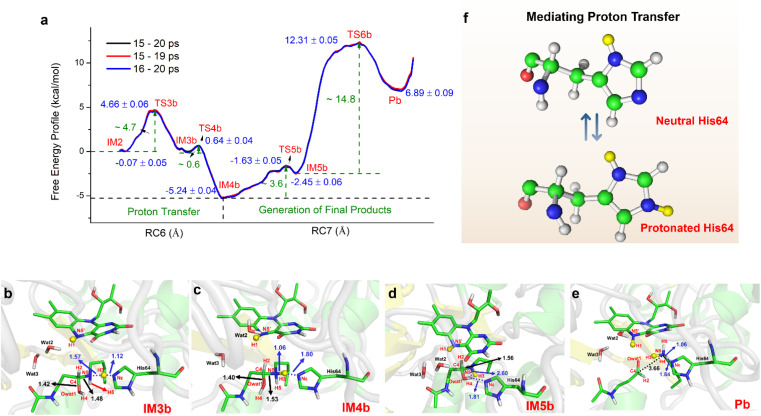
Generation of *N*-acetyl-3-aminopropanal and spermidine with two protonated terminal amino groups. (a) Free energy pattern during the generation of *N*-acetyl-3-aminopropanal and spermidine with two terminal amino groups protonated, calculated on the basis of QM/MM MD simulations (RC6 = *d*_Nε-H3_ − *d*_N5-H3_, RC7 = *d*_C4-N5_ − *d*_N5-H4_ − *d*_Nε-H4_). (b) The intermediate with the diversion of the hydroxyl group. (c) The intermediate after proton transfer from His64 to hydroxylated polyamine. (d) Changes in the orientation of the hydroxyl group that forms a hydrogen bond with His64. (e) Competition between His64 and the N5 of polyamine for H4 to form the final products of *N*-acetyl-3-aminopropanal and spermidine with two protonated terminal amino groups. (f) His64 is the only residue participating in the chemically regulated step, and it serves as a “proton-shuffler”, mediating the proton transfer process.

**Fig. 5:**
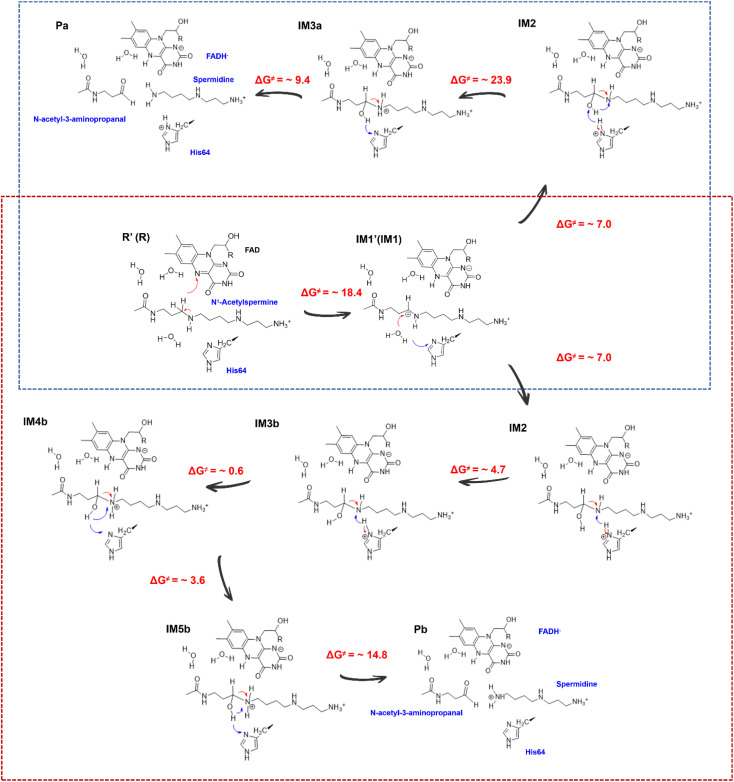
Mechanism for the generation of *N*-acetyl-3-aminopropanal and different protonated spermidines.

If the proton donor is protonated His64, the proton transferring to the amino group overcomes the barrier of ∼4.7 kcal mol^−1^ (see Fig. 4a and S25†), and thus the proton of His64 is more attractive to the amino group than the hydroxyl group of the substrate. Initially, the orientation of the hydroxyl group is changed to give space for proton transfer from His64 (Fig. 3f, 4b, Sections S6, S25, and S27†). Then, it overcomes a small barrier to reach IM4b to complete the proton delivery (Fig. 4b and c). During this process, C4-N5 gradually stretches to 1.53 ± 0.04 Å without breakage. Herein, both the Nε of His64 and the –NH_2_^+^- group probably accept the proton of the –OH group on C4. The free energy profile (Fig. 4a, S23, S26, and Section S1.6†) shows that the formation of the final products needs to overcome two barriers of ∼3.6 and ∼14.8 kcal mol^−1^. The first barrier corresponds to the torsion of the hydroxyl and imino groups in polyamine, and the second one corresponds to transfer of a proton to the protonated imino group and the cleavage of C4-N5 (Fig. 4c and d). Note that the competition between the two receptors for the proton and the cleavage of C4-N5 to generate the final stable products of protonated spermidine and *N*-acetyl-3-aminopropanal follow a concerted mechanism (Fig. 3–5, Sections S7 and S26†). Sampling based on different reaction coordinates further demonstrates the mechanism. An energetically unfavorable non-intermediate state is identified for proton transfer from the hydroxyl group to His64, characterized by an upward free energy barrier (see Section S1.7 and Fig. S28–S29†).

Consequently, the production of *N*-acetyl-3-aminopropanal and *N*-acetyl-3-aminopropanal is the most favorable route. Herein, His64 provides a proton to the imino group to trigger the following reactions of proton transfer, and C4-N5 cleavage is achieved with a lower barrier compared with substrate-self assisted proton transportation. In addition, on the basis of the following unrestrained MM MD simulations, SP1 and SP3 (or SP2) would thermodynamically seek more comfortable locations to generate the final enzyme-product state with strong binding affinity (see Fig. S30†). This will probably benefit the progress of the reaction.

### Detour releases *N*-acetyl-3-aminopropanal and various protonated forms of spermidine

**Fig. 6:**
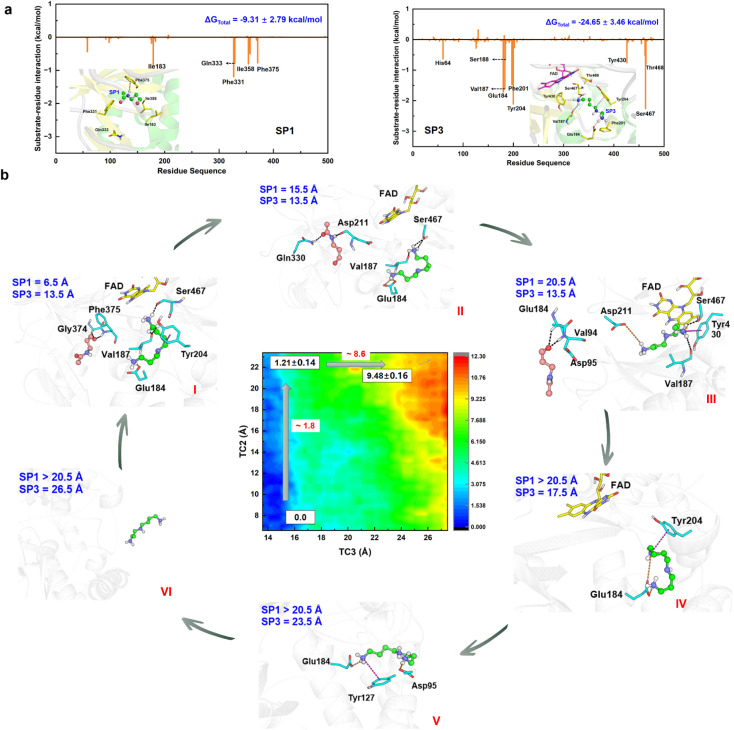
Detour release mechanism of products in APAO catalysis. (a) Static binding mode and free energy decomposition for SP1 and SP3 with APAO in the active site. (b) Free energy profile and key snapshots for the release of SP1 and SP3.

The chemical step generates two types of catalytic product. The first set is *N*-acetyl-3-aminopropanal (SP1) and spermidine with one protonated terminal amino group (SP2), and the second set is SP1 and spermidine with two protonated terminal amino groups (SP3). The second set is more favorable, and we mainly focus it. The hydrogen bond and conjugated interaction from hot residues is the main driving force for the localization of the two products (Fig. S30†). The van der Waals and electronic interactions contribute most to SP1 and SP3, respectively (Fig. 6a, Section S8, Fig. S31–S32, and Table S4–S5†). For their release Section S9, Fig. S33 and Table S6–S7†), two possible channels are discovered for SP1 and SP3. For SP1, the “door” for the most favorable channel localizes among Glu84–Pro103, Ser365–Ser367, and Pro327–Gln330. For SP3, the “door” of the most favorable channel comprises Tyr204–Pro208, Leu116–Phe136, and Leu93–Pro103.

The 2D-PMF (Fig. 6b) shows three possible mechanisms (Section S1.8 and Fig. S34–S35†). (i) SP1 is released first by crossing a barrier of ∼1.8 kcal mol^−1^, and SP3 moves away from the active site completely by overcoming a barrier of ∼8.6 kcal mol^−1^ (ii) SP3 is released before SP1. A large pocket is visible as SP3 leaves, and SP1 is easily attracted by FAD to fluctuate in the pocket because of its small size. Therefore, it directly leads to a reduction in the resistance of SP1 in the spatial site. The free energy shows up the tendency for there not to be a stable platform after SP3 release. (iii) The two products are released simultaneously with a higher free energy barrier than the first mechanism. Accordingly, the most reasonable mechanism is the first one, which can be divided into six stages based on the free energy profile and conformational changes (Fig. 6b and S36†).

In the first stage (6.5 Å ≤ TC2 ≤ 11.5 Å, 13.5 Å ≤ TC3 < 15.5 Å), the free energy is relatively gentle, which seems to be an initial binding state. SP3 stays at the active site, while SP1 begins to leave the active site. The conformation and location of SP1 are very flexible. At the outset, Asn313 and Phe375 create a driving force that leads to the elimination of the original site in SP1 through hydrogen bond and σ–π interactions, respectively. Subsequently, as SP1 progresses along TC2 within a range of 9.5 to 11.5 Å, its movement is notably influenced by aromatic and classical hydrogen bond interactions mediated by Phe331 and Gln333.

In the second stage (11.5 Å < TC2 ≤ 17.5 Å, 13.5 Å ≤ TC3 < 15.5 Å), the free energy shows an apparent upward trend. SP1 is livelier than that in the first stage, whose orientation shows a distinct change, which is probably caused by Asp211 and Gln330. For Asp211, it appears with almost 100% probability between 12.5 and 15.5 Å, forming a strong hydrogen bond with SP1, which initially contributes most to the motion. For Gln330, the probability of occurrence is 80% between 13.5 and 17.5 Å, which further facilitates SP3 to adjust its direction and select the escape channel.

In the third stage (17.5 Å < TC2 ≤ 20.5 Å, 13.5 Å ≤ TC3 ≤ 14.5 Å), the free energy gently flattens out without rising further, suggesting that the system reaches a local minimum platform. SP1 is exposed to the water environment with small constraint, and it also swings around the exit.

In the fourth stage (TC2 > 20.5 Å, 14.5 Å < TC3 ≤ 18.5 Å), the free energy shows a mild upward trend, and SP1 is completely released. The pocket becomes larger by the relaxation of the protein. Because of the weak binding and open space, the conformation of SP3 experiences an apparent change. Glu184 plays an important driving role by a strong ion-pair interaction formed with SP3, which has almost 100% probability. Moreover, cation–π interactions from Tyr204 and His64 influence the release of SP3.

In the fifth stage (TC2 > 20.5 Å, 18.5 Å < TC3 ≤ 25.5 Å), the free energy continues to rise. The conformation of SP3 is stretched and it is still moving toward the outside of the protein. The role of Glu184 is gradually becoming an obstacle to SP3 release, and its effect is gradually reduced. Besides, ion-pair electronic interactions arising from Asp95 and Glu131 along with the σ–π interaction from Tyr127 make a tractive effort for SP3 transportation. Then, SP3 breaks away from the hydrogen bond and forms ion-pair interactions with Thr96 and Glu131, and swings around the door.

In the final stage (TC2 > 20.5 Å, TC3 > 25.5 Å), the free energy reaches a stable platform without rising. There is only one weak interaction left between Asp95 and SP3, and thus the water easily attracts SP3 to assist its complete exposure to the solvent. In this stage, both products fully escape from the protein.

It should therefore be noted that SP1 and SP3 experience obvious conformational changes in their release process, which follow a detour stepwise mechanism. The hydrogen bond interaction is the main driving force for SP1 release, and the strong ion-pair interaction contributes most to SP3 escape. Conjugate interactions also influence the process, but a steric effect caused by the large ring is not apparent.

### Modelling of enzymatic panorama and role of His64

As mentioned above, enzymatic panorama including bond and non-bond process were built. First, the –COO^−^ group of Asp211 initially recognizes *N*^1^-acetylspermine by strong ion-pair electronic interaction, which is beneficial for the polyamine to select the favorable channel to start the catalysis. In addition, hydrogen bond and cation–π interactions provide the main driving force for reactant transportation. A strong exothermic phenomenon is observed, which may be caused by the formation of new interactions and self-adaption of *N*^1^-acetylspermine, providing crucial power for the subsequent chemical reaction and product release. Glu184, Val187, Ser188, Phe201, Tyr204, Asn313 and Phe375 are discovered as hot residues for reactant static binding in the active site, among which Asn313 contributes the most, which agrees with and explains the previous experimental data.^18^ Second, the intermediates and transition states as well as their stability and binding mode are revealed for the first time. *N*-Acetyl-3-aminopropanal and spermidine with two protonated terminal amino groups would be more favorably generated in the chemical reaction. Hydride transfer from *N*^1^-acetylspermine to FAD occurs first, which follows an H-shift coupled electron transfer mechanism. The participation of water is a feature of the attraction of polar polyamine. With the assistance of His64, the water attacks the substrate to form a hydroxylated intermediate and FADH^−^. The protonated imino group is generated following the deprotonation of His64. Afterward, the hydroxyl group transfers a proton on the –NH_2_^+^- group with the help of His64 to trigger the cleavage of C4-N5 to form the final products. Note that hydride transfer is a rate-limiting step in the chemical step, which also determines the whole enzymatic catalytic efficiency. The corresponding free energy barrier, considering tunneling correction and computational method of estimation, accords well with the enzyme kinetics experiment.^20^ Thirdly, the release of the two products follows a detour stepwise mechanism, and the non-bonding step also influences the enzymatic efficiency. *N*-Acetyl-3-aminopropanal flips because of its small size and various surrounding interactions, and the hydrogen bond is the driving force for its escape. The double-protonated spermidine experiences distinct conformational changes between a microroll and a stretch, and the strong ion-pair interactions are beneficial for its movement.

It is noteworthy that during the entire process, the chemical transformation determines the whole enzymatic efficiency, and His64 stands out as the sole participant in the chemical reaction as a “proton-shuffler” that mediates proton transfer three times (Fig. 3–5). First, it captures a proton from water that helps in the formation of the hydroxylated intermediate. Second, its protonated form provides a proton to the –NH– group of the polyamine substrate. Third, it participates in the formation of the final product and plays a competing role in proton transfer. Tormos *et al.*^20^ found that the His64 mutant decreased the catalytic rate constant (*k*_cat_) compared with wild-type acetylpolyamine oxidase (APAO). Our current study reveals a detailed mechanism underlying the phenomenon. The following pharmacological experiment will further reveal the role of His64.

### Regulation of polyamines and anti-tumor effect of His64

**Fig. 7:**
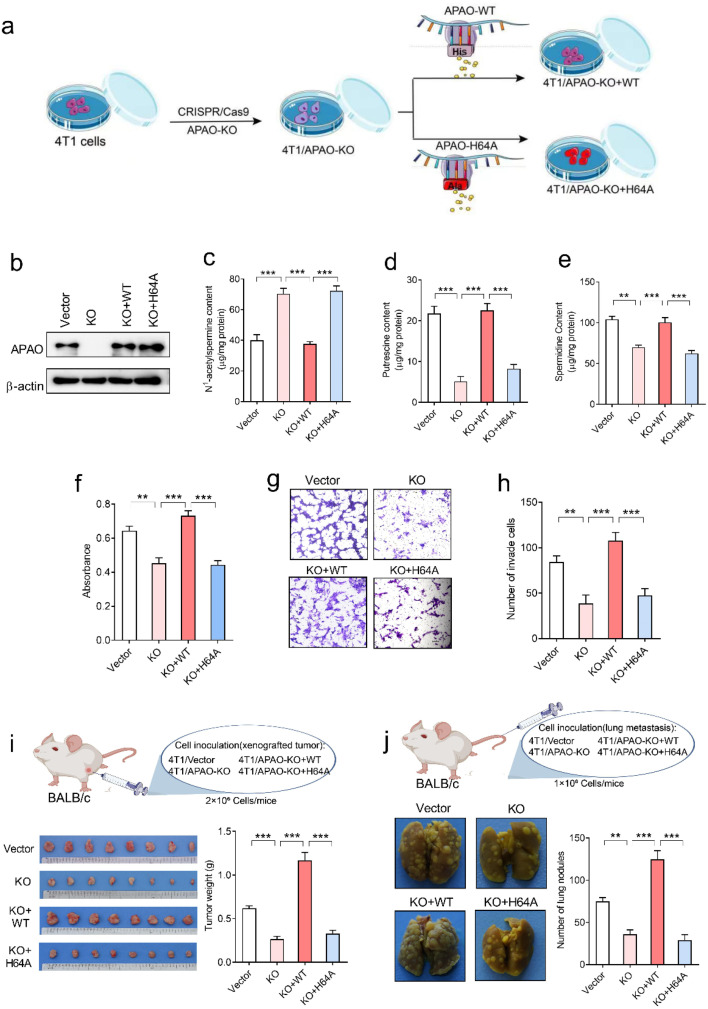
Effects of the His64 residue of APAO on polyamine metabolism and tumor progression. (a) Schematic diagram establishing a 4T1 cell model for APAO gene editing. (b) Western blot analysis of APAO expression in indicated 4T1 cells, KO: APAO knock out, WT: APAO wild-type, H64A: APAO H64A mutant. (c) HPLC analysis of *N*^1^-acetylspermine in indicated cells, ***p < 0.001, one-way ANOVA, *n* = 5 per group. (d) HPLC analysis of putrescine, ***p < 0.001, one-way ANOVA, *n* = 5 per group. (e) HPLC analysis of spermidine in indicated cells, **p < 0.01, ***p < 0.001, one-way ANOVA, *n* = 5 per group. (f) MTT analysis of the proliferative activity of the indicated 4T1 cells, **p < 0.01, ***p < 0.001, one-way ANOVA, *n* = 6 per group. (g) Representative images of migrated cells. (h) Quantitative analysis of the migratory activity of the indicated 4T1 cells, **p < 0.01, ***p < 0.001, one-way ANOVA, *n* = 5 per group. (i) Representative images of xenografted tumors and quantification of tumor weight collected from vector, KO, KO + WT and KO + H64A groups. ***p < 0.001, one-way ANOVA, *n* = 8 per group. (j) Representative images of lung metastasis and quantification of tumor metastatic nodules from vector, KO, KO + WT, and KO + H64A groups. **p < 0.01, ***p < 0.001, one-way ANOVA, *n* = 8 per group.

To investigate whether the His64 residue affects the catalytic activity of the APAO enzyme, we first knocked out endogenous APAO in 4T1 mouse breast cancer cells using CRISPR/Cas9 and reconstituted the expression of WT APAO or APAO H64A mutant in endogenous APAO-depleted 4T1 cells (Fig. 7a). The construction efficiency was confirmed by western blot analysis (Fig. 7b). Then, high-performance liquid chromatography (HPLC) was performed to examine the content of *N*^1^-acetylspermine, spermidine and putrescine in these cells. The results show that the content of *N*^1^-acetylspermine in APAO-depleted cells is significantly increased, but spermidine and putrescine are dramatically decreased compared with the vector group (Fig. 7c–e). Reconstituted expression of APAO, but not the APAO H64A mutant, significantly restores the increased *N*^1^-acetylspermine and reduces spermidine and putrescine in endogenous APAO-depleted cells (Fig. 7c–e). These results indicate that the His64 residue plays a critical role in catalyzing *N*^1^-acetylspermine to generate spermidine and putrescine.

Although suppression of APAO activity can potentiate the anti-tumor effects of cytotoxic drugs by increasing apoptosis of cancer cells,^24,25^ whether APAO itself promotes or suppresses cancer cell was still unknown. Therefore, we herein investigate the direct role of APAO in cancer cell proliferation and migration. The MTT (3-[4,5-dimethylthiazol-2-yl]-2,5-diphenyltetrazoliumbromide) assay results show that APAO knockout dramatically inhibited 4T1 cell viability *in vitro*, and restoration of APAO expression increased cell viability in APAO-depleted 4T1 cells (Fig. 7f). Based on the above results, that mutating His64 would partially inhibit the catalysis of APAO, we also expressed APAO H64A mutant in APAO-depleted 4T1 cells and examined cell proliferation. As expected, reconstitution of the APAO H64A mutant fails to restore cell viability (Fig. 7f). Similarly, the transwell migration assay reveals that reconstituted expression of APAO, but not the APAO H64A mutant, significantly restores the migration ability in endogenous APAO-depleted 4T1 cells (Fig. 7g and h). Subsequently, a mouse xenograft model and metastatic cancer model were established to investigate the significance of the APAO H64 residue *in vivo*. As anticipated, APAO knockout significantly inhibited tumor growth and metastatic seeding *in vivo*, whereas reconstituted expression of the APAO H64A mutant, unlike its WT counterpart, failed to restore tumor growth and metastasis *in vivo* (Fig. 7i and j). Accordingly, APAO plays a positive role in the progress of cancer cells and suppressing it may be useful for antineoplastic therapies. Mutating His64 is a useful route for inhibiting the function of APAO.

## Conclusions

Our main purpose in the present work is how to construct a map among enzymatic catalysis, polyamine regulation, and tumors, which is of great significance in the large polyamine research area. An enzymatic-related network was first established using extensive QM(DFT)/MM MD and MM MD simulations combined with pharmacological experiments (Fig. S37†). Traditional enzymatic catalytic calculations primarily emphasize the chemical steps, neglecting the delivery of a substrate or product. In contrast, our study takes a comprehensive approach, considering both these aspects to uncover the intricate enzymatic features at play. Moreover, dynamic snapshots are sampled throughout the whole process, which describes the entire catalytic process more reliably and vividly. The environment of the protein is dynamic, and the addition of molecular dynamics on QM/MM also makes up for the shortcomings of the static QM/MM. The calculations reveal that Asp211 easily captures polyamine *via* strong ion interaction. Hydrogen bond and cation–π interaction drives the transportation of polyamine from outside to the active site of the enzyme. In addition, two sets of products are generated, and release follows a stepwise mechanism. It should be noted that the rate-determining step has been discovered to localize at the chemical regulation step, and His64 is the only residue participating in the reaction. It is identified as a key mediator of three consecutive “protonation–deprotonation” processes, effectively facilitating a proton shuffle within the enzymatic mechanism. His64 directly promotes enzymatic catalysis. Therefore, whether the residue affects the play of enzyme function and further affects the regulation of polyamines is of great interest to us.

Inspired by the electronic and atomic simulations, a novel strategy arising from His64 responsible for a network from microcosmic to macroscopic scale was modeled. Regarding the connection between His64 and polyamine regulation, HPLC analysis confirmed that the His64 mutant leads to an increase in the reactant and a decrease in the products, indicating that His64 directly affects the function of enzymatic catalysis. With regard to APAO and its anti-tumor effect, directly suppressing APAO by mutating His64 was first identified to further inhibit tumor cell and tumor tissue growth and migration *in vitro* and *in vivo*. This anti-tumor effect is likely to be due to the changes in polyamine homeostasis.

In additional revelation, polyamine and its derivatives are easily protonated in physiological conditions, and if the residues around the external surface of the possible target have a negatively charged side chain, it will recognize the polyamine compounds and help them enter the active site, which may be a notable feature not just for the current protein. Besides, the polyamine is polar and charged, and thus, attendant water may be a universal feature for other enzymatic processes related to polyamine by electrostatic interaction. For the inhibitor design of APAO, reducing the solvent energy and increasing the donor or receptor of the hydrogen bond and ion-pair can improve the static-kinetic behavior of potential inhibitors. Inhibitors can also be designed based on the structures of the reactant, intermediate and product. Furthermore, it is also a good choice to design an inhibitor that targets His64 directly. The discovery of the present chemical mechanism might be crucial in the biosynthesis of polyamine derivatives, providing a clue to comprehend the enzymatic process of metal-independent oxidase with FAD. Moreover, modeling the enzymatic network starting from a microscopic catalytic mechanism with a reliable scheme could accurately identify the residue sites, saving experimental resources and could also be applied to other systems. This groundbreaking study represents the first in-depth exploration of polyamine metabolism at the electronic level, providing crucial insights into the fundamental regulation of enzymes. By delving into the underlying mechanisms, this research paves way for future advances in the design of polyamine compounds and field of anti-tumor studies.

## Computational and experimental details

### Building an APAO_o*x*_·*N*^1^-acetylspermine·FAD complex

In experiments, Sjögren *et al.*^18^ proposed that the X-ray crystal structure of APAO_o*x*_ cocrystallized with *N*^1^-acetylspermine and FAD (PDB ID: 5LFO) is a more catalytically relevant structure. This is because APAO shows its highest activity at pH 8.0 and almost loses activity at pH 5.5. Soaking of the crystal with the substrate at pH 5.5 did not cause bleaching, and APAO is thought to still be in its oxidized state. Therefore, the initial structure of the enzyme–substrate complex built on APAO_ox_·*N*^1^-acetylspermine·FAD can simulate the reactant state well. The missing fragments of Asp95–Leu102, Asn138–Met1144, and Ser365–Ser369 are complemented by the biopolymer module in the Sybyl-X 2.1 software^26^ on the basis of RMS value and homology score. Herein, two water molecules are observed between FAD and *N*^1^-acetylspermine that are probably important for controlling the distance between the two substrates, and one is detected around *N*^1^-acetylspermine that is probably the source of APAO-catalyzed oxidation of *N*^1^-acetylspermine. Therefore, they are retained and the other original water molecules are removed. The proton states of ionizable residues are determined at pH = 8.0 *via* H++^27^ as well as the hydrogen bond network around them. For *N*^1^-acetylspermine and FAD, the Amber GAFF force field^28^ was chosen to describe them, and their charge parameters were calculated at HF/6-31G(d) level by the restrained electrostatic potential (RESP) methodology^29^ using the Gaussian 09 program.^30^ With regard to APAO, the Amber99SB force field^31,32^ was utilized. The whole enzyme–substrate complex was dissolved in a 78.0 × 112.5 × 87.9 Å TIP3P^33^ cubic water box with a buffer distance of 10 Å between the water box edge and closest protein atoms. Na^+^ ions were added to keep the system neutral. For the enzyme complexed with products of SP1, SP2, and SP3, the treatment was the same with the enzyme complexed with the reactant. The topological parameters and initial coordinates were produced using the tleap module in Amber 18 software.^34^

### MM MD simulations

First, the interactions among atoms were corrected by minimization. Then, the system was heated gradually from 0 to 300 K under the NVT ensemble, and further a series of simulations were performed under NPT and NVT ensembles alternately to relax the density to 1 g cm^−3^. Afterwards, 40 ns classical MD simulations with the NVT ensemble using periodic boundary conditions were performed with FAD restrained to only 2 kcal mol^−1^ Å^−2^ to prevent the protein moving to the edge of the water box. A cutoff distance of 12 Å was applied for calculation of electrostatic and van der Waals interactions. Long-range electrostatic interaction was treated with the particle mesh Ewald (PME) method.^35–37^ An integration time step of 1 fs was used, and the collision frequency was set as 1 ps^−1^. The SHAKE algorithm^37^ was applied to constrain all hydrogen-containing bonds. The Langevin method was used to maintain the temperature at 300 K. For the enzyme complexed with products of SP1, SP2, and SP3, 50 ns MM MD simulations were performed. For each system, the free energy decomposition was determined using the MM/GBSA method on the basis of 200 snapshots extracted from the last 5 ns of the equilibrium trajectory. The molecular dynamic simulations were performed using Amber 18 software.^34^

### RAMD MD simulations

On the basis of stable snapshots, combined random acceleration molecular dynamic (RAMD) and molecular dynamic (MD) simulations^38–40^ were carried out to identify the possible channels of *N*^1^-acetylspermine delivery by APAO. The method involves enough classic relaxation by classic MD simulations to avoid a wrong direction caused by a larger random force. Herein, the threshold distances of 0.005, 0.004, 0.003, and 0.002 Å and random accelerations of 0.50, 0.40, 0.30, and 0.20 kcal Å^−1^ g^−1^ were applied to the center of mass of the reactant. A total of 112 trajectories were obtained to use statistical analysis and find the most favorable channel. For the identification of product release pathways, the same parameters were used, and 224 trajectories were acquired for the following statistical analysis. The calculations in this part were carried out using NAMD software.^41^

### Umbrella sampling

For the most favorable channel, the mechanism and corresponding thermodynamic and dynamic properties for the entrance of the reactant and release of two products were studied using MM MD simulations combined with umbrella sampling technology. One- and two-dimensional free energy profiles were obtained. The appropriate delivery coordinate and biasing harmonic potential were chosen with 20 ns NVT MD simulations for each window. The probability distributions were applied for free energy profile mapping by employing the weighted histogram analysis method (WHAM).^42,43^ The calculations were performed using Amber18 software.^34^

### Building of QM/MM MD model

When the reactant binds into the active site of an enzyme, a chemical reaction will take place. In a previous study, Hammes-Schiffer's group found that the distance between donor and acceptor relative to its equilibrium value is vital in a proton-coupled electron transfer reaction.^22^ Moreover, the water molecules around the two substrates are flexible and have different orientations and locations. Therefore, to reinstate the catalytic center of the crystal structure as far as possible and keep the system fully relaxed, when the temperature reaches 300 K, 50 ns NVT MM MD simulations were executed, and a very small restraint force of 2 kcal mol^−1^ Å^−2^ was applied on FAD, *N*^1^-acetylspermine, His64, and three crystal water molecules in the active site. The QM/MM system was prepared by deleting the water molecules beyond 28 Å from C21 of FAD on the basis of stable snapshots. The residues of His64, three main water molecules, part of FAD and *N*^1^-acetylspermine were chosen as the QM region (see Fig. S38†), which were treated with the B3LYP functional^44,45^ and 6-31G(d) basis set. This level has been used successfully in many enzyme catalysis systems.^46–50^ Moreover, three levels were also tested (see Section S4 and Table S3†). The other atoms were considered as the MM region. The protein was described by the Amber99SB force field^31,32^ and water molecules were employed by the TIP3P model.^33^ The improved pseudo-bond approach^51–53^ was used to depict the QM/MM boundary.

### QM/MM MD simulations

For all electronic structures along the reaction path, the MM atoms were relaxed for 200 ps by classic MD simulations with QM atoms frozen to obtain a promising reaction profile. On the basis of the stable snapshots generated in the last step, 20 ps QM/MM MD simulations were carried out with umbrella sampling technology for each window. The spherical boundary condition was applied, and all atoms within a radius of 25 Å from the spherical center atom were free to move. The cutoff radii were set as 18 and 12 Å to deal with the electrostatic and van der Waals interactions in the MM subsystem, respectively. The Beeman algorithm^54^ was applied with a time step of 1 fs, and the Berendsen thermostat^55^ was used to maintain the temperature at around 200 K. The probability distributions for different periods of time along the reaction coordinate were employed to determine the free energy profile by using the weighted histogram analysis method (WHAM).^42,43^ All the *ab initio* QM/MM calculations were performed by applying Q-Chem 4.0 (ref. 56) and Tinker 4.2 packages.^57^

### Tunneling effect evaluation

The coordinate and thermodynamic data of reactant and transition state were obtained at M06-2X/6-31+G(d), M06-2X/6-31G(d), and B3LYP/6-31G(d) levels. Then, the tunneling effect was estimated on the basis of transition state theory (TST) including one-dimensional quantum mechanical tunneling treatments through the Wigner correction.^21^ The vibration scaling factors were set as 0.97, 0.97, and 0.98,^58^ respectively for the three levels. Gaussian 09 (ref. 30) and KiSThelP software^21^ were employed.

### Cell lines and cell culture

Murine breast cancer cell line 4T1 was obtained from the Cell Bank of the Chinese Academy of Sciences (Shanghai, China) and cultured in RPMI-1640 medium (Gibco, Carlsbad, CA) containing 10% fetal bovine serum (HyClone®, UT, USA) at 37 °C in an atmosphere containing 5% CO_2_.

### DNA constructs, mutagenesis and lentiviral transduction

APAO was knocked out in 4T1 cells using the CRISPR/Cas9 genome editing system. Briefly, the 4T1 cells were cotransfected with CRISPR/Cas9 and HDR plasmids by Lipofectamine 2000 (Invitrogen, Thermo Fisher Scientific, Inc). After 2 days of transfection, the cells were selected with puromycin for 1 week. To reconstitute APAO WT or APAO H64A in APAO-knockout cells, the lentivirus vectors expressing APAO WT or APAO H64A were constructed by inserting the cDNA into pCDH-CMV-MCS-EF1-CopGFP-T2A-Puro. The APAO H64A mutagenesis was constructed with a Fast Mutagenesis System Kit (Cat# FM111-01, TransGen Biotech, Beijing). The lentivirus particles were packaged by transfecting 293T cells with envelope plasmid pMD2.G and packaging plasmid psPAX2. The lentivirus productions were harvested after 48 h of transfection. Next, 4T1 cells were infected with viruses and selected in the presence of 1.5 μg mL^−1^ puromycin (Sigma, St. Louis, USA) for 1 week.

### Measurement of intracellular polyamines

The concentration of natural polyamines (*N*^1^-acetyl-SPM, spermidine, putrescine) in 4T1 cells was determined by high performance chromatography (HPLC), as previously described.^59^ Briefly, the cells were harvested in 200 μL of PBS, and 200 μL of hexanediamine (1.624 μg mL^−1^) was added as an internal standard. After adding 200 μL of dansyl chloride (5 mg mL^−1^), the mixtures were incubated at 50 °C for 30 min, and the reaction was terminated by the addition of 1 mL of ethyl acetate. The supernatant containing the polyamines was functionalized with dansyl chloride and purified by organic filtration. Further, 20 μL of the sample was injected onto an XDB-C18 column (4.6 × 250 mm, Agilent Technologies), which achieved excitation at 340 nm and measured emission at 515 nm with a fluorescence detector. The solvent system consisted of methanol and water, running at 65% (v/v) to 100% (v/v) methanol within 25 min at a flow rate of 1 mL min^−1^.

### Cell proliferation assay

Cell proliferation was determined using a 3-(4,5-dimethylthiazol-2-yl)-2,5-diphenyltetrazoliumbromide (MTT) assay. Briefly, 4T1 cells were seeded in 96-well plates at an initial density of 5 × 10^3^ cells per well in 100 μL of culture medium and allowed to grow for 48 h. Further, 10 μL of 0.5 mg mL^−1^ MTT (Sigma-Aldrich) solution was added to the medium for further 4 h at 37 °C. The supernatants were then removed, and 150 μL of dimethyl sulfoxide (DMSO) was added to dissolve the formed formazan crystals. Cell viability was measured at 570 nm using a microplate reader (BioTek Instruments, Inc., Winooski, VT, USA).

### Cell migration assay

Cell migration was carried out using a Transwell chamber (8 μm, Corning, NY, USA). Briefly, 5 × 10^4^ 4T1 cells in serum-free medium were plated in the upper chamber system. RPMI 1640 medium containing 10% FBS was placed in the lower chambers. After incubation for 24 h, the cells that had migrated to the bottom insert surface were stained with 0.1 mg mL^−1^ crystal violet in 20% methanol. The number of migrated cells was determined by counting five random fields on each membrane.

### Subcutaneous xenograft and experimental metastasis mouse models

Female Balb/c mice (aged 5–6 weeks and weighing 20–25 g) were purchased from Beijing Weitong Lihua Animal Co. The mice were kept in an animal room at a constant temperature (23 °C) and humidity (60%), with a 12 h light–dark cycle, and had free access to food and water. All the experimental protocols and procedures were approved by the Animal Care and Ethics Committee of Henan University (Kaifeng, China). For the *in vivo* tumorigenesis experiment, 2 × 10^6^ 4T1 cells were injected subcutaneously into the right flank of the Balb/c mice. Two weeks later, the mice were euthanized by cervical dislocation. Tumor tissues were harvested and weighed. For the metastasis model, a total of 1 × 10^6^ 4T1 cells were injected into the lateral tail vein of the Balb/c mice. Six weeks after injection, the mice were euthanized by cervical dislocation. The lungs were harvested and fixed with 4% paraformaldehyde for 1 day, and the number of lung metastases were counted.

### Western blot assay

Total protein from the cancer cells was extracted using ice-chilled RIPA lysis (Beyotime Biotechnology, China) containing 0.25 mM phenylmethylsulfonyl fluoride. The protein concentration was determined with a BCA protein assay kit (Thermo Fisher Scientific, Inc). Equal amounts of proteins were separated by 10% sodium dodecyl sulfate polyacrylamide gel electrophoresis (SDS-PAGE) and transferred onto polyvinylidene difluoride membranes. After blocking with 5% nonfat milk at room temperature for 1 h, the membranes were incubated with primary antibodies anti-APAO (1:500, Sigma-Aldrich, USA) and anti-β-actin (1:1000, Santa Cruz, USA) overnight at 4 °C. Subsequently, the membranes were incubated with horseradish peroxidase-conjugated secondary antibodies for 1 h at room temperature. Protein bands were detected using Enhanced Chemiluminescence Plus Reagent (Thermo Fisher Scientific, Inc.) and visualized using a FluorChem E Imager System (Protein Simple, San Jose, CA, USA).

### Statistical analysis

All data in this study are expressed as the mean ± standard error of the mean (SEM). Statistical analyses were performed using GraphPad Prism 8.0 (GraphPad Software, Inc., La Jolla, CA, USA). One way analysis of variance (ANOVA) following Tukey post hoc tests was used to compare the mean values of multiple experimental groups. *p* < 0.05 is considered to indicate a statistically significant difference.

## Data availability

Essential data are provided in the main text and ESI.† Data can be made available from the corresponding author upon reasonable request.

## Author contributions

Y. Z., C. W., D. F., and S. X. designed the research. Y. Z and R. W. supervised the computational work. Y. Z. performed the QM/MM MD simulations and data analysis. Z. Z., Y. Z., and X. L. performed MM MD simulations and data analysis. Y. Z. and J. Z. wrote statistic scripts. D. F. carried out the experimental work and data analysis. Y. Z., Z. Z., B. G., J. Z., and X. L., drew figures. Y. Z., Z. Z., and D. F. wrote the first draft. R. W., P. L, and J. S revised the draft. Y. Z., D. F., and C. W. wrote the final manuscript.

## Conflicts of interest

There are no conflicts to declare.

## Supplementary Material

SC-015-D3SC06037C-s001
