# Supplementary material for: Enzymatic-related network of catalysis, polyamine, and tumors for acetylpolyamine oxidase: from calculation to experiment
Source: Chem Sci. 2023 Dec 29;15(8):2867–82. doi: 10.1039/d3sc06037c (PMC10882482; doi:10.1039/d3sc06037c)
Supplement: SC-015-D3SC06037C-s001 [file SC-015-D3SC06037C-s001.pdf]

Electronic Supplementary Material (ESI) for Chemical Science.
This journal is © The Royal Society of Chemistry 2023

Supporting Information

Enzymatic-Related Network of Catalysis, Polyamine, and Tumor for Acetylpolyamine Oxidase: from Calculation to Experiment

Dong Fang,‡[ab] Zhiyang Zhang,‡[a] Jihang Zhai,[a] Baolin Guo,[a] Pengfei Li,[d] Xiaoyuan Liu,[a] Jinshuai Song,[e] Songqiang Xie,[b] Ruibo Wu,[c] Yuan Zhao*[a] and Chaojie Wang*[a]

[a] *The Key Laboratory of Natural Medicine and Immuno-Engineering, Henan University, Kaifeng 475000, P.R. China.* ***E-mail: zhaoyuan@henu.edu.cn, wcjsxq@henu.edu.cn***

[b] *School of Pharmacy, Henan University, Kaifeng 475000, P.R. China.*

[c] *School of Pharmaceutical Sciences, Sun Yat-sen University, Guangzhou 510006, P.R. China.*

[d] *Department of Chemistry and Biochemistry, Loyola University Chicago, Chicago, Illinois 60660, United States.*

[e] *Green Catalysis Center, and College of Chemistry, Zhengzhou University, Zhengzhou 450001, P.R. China.*

‡ These authors contributed equally.

Contents

S1. Convergence of PMF curves 1

S2. The detail delivery process of reactant in second stage 3

S3. The reaction coordinate selection for formation of FADH and hydroxylated intermediate 3

S4. Wigner tunneling correction[1] and calculated level estimation 4

S5. One-dimension free energy profile and mechanism for formation of FADH and hydroxylated intermediate 4

S6. The orientation changes of hydroxyl group of N^1-acetylspermine 5

S7. The competition of Nε of His64 and the -NH_2^+- group of polaymine for proton of hydroxyl group on C4 5

S8. The binding mode and binding free energy decomposition for SP1 and SP3 (SP2) 5

S9. RAMD MD simulations of two sets of products 6

Fig. S1. The TC1 for reactant entrance into the active site of APAO 7

Fig. S2. The free energy profiles obtained for the periods of 0 ~ 5, 5 ~ 10, and 10 ~ 15 ns obtained by MM MD combined umbrella sampling. 8

Fig. S3. The represent snapshot of the windows for the second stage obtained by the cluster analysis in the last 5 ns of MM MD simulations. 9

Fig. S4. The RMSD of the APAO in the 40 ns MM MD simulations of the complex system 10

Fig. S5. The free energy decomposition for the key residues in the static binding of APAO and reactant. 11

Fig. S6. The stabilization of hydrogen bond interactions in the MM MD simulations of complex system. 12

Fig. S7. The reaction coordinate test in the QM/MM RCD simulations on the basis of the stable state obtained in MM MD simulations (The reaction coordinate is defined as - $d_{Owat1\text{-}C4}$ - $d_{H1\text{-}N5'}$ (RC1)). 13

Fig. S8. The comparison of R-test1, IM1-test1, and IM2-test1 for the bond length of Owat1-C4 in the 10 ps QM/MM MD simulations. 14

Fig. S9. The relative energy comparison for fixing RC2 and driving RC3 (RC4) in QM/MM RCD simulations. 15

Fig. S10. The changes of bond length for RC3 and RC4 for fixing RC2 and driving RC3 (RC4) in QM/MM RCD simulations. 16

Fig. S11. The relative energy profile obtained by 2D-QMM/MM RCD simulation on the basis of RC2 and RC4. 17

Fig. S12. The free energy profile of different periods for generation of $FADH^-$ and hydroxylated polyamine obtained by QM/MM MD simulations combined with umbrella sampling on the basis of RC2 and RC4. 18

Fig. S13. The reactant, intermediates, and transition states along with free energy profile in generation of $FADH^-$ and hydroxylated polyamine obtained by last 5ps QM/MM MD simulations combined with umbrella sampling on the basis of RC2 and RC4 19

Fig. S14. The bond length changes of Owat1-C4, Nε-H3, and Owat1-H3 along the RC4 in the last 5 ps QM/MM MD simulations. 20

Fig. S15. The structure of reactant and transition state obtained by different DFT methods, and the corresponding Cartesian coordinates is listed in Appendix. 21

Fig. S16. The free energy profiles of different periods obtained by one dimension QM/MM MD simulations combined with umbrella sampling on the basis of RC2 and RC4. 22

Fig. S17. The structure of reactant, transition state, and intermediate together with corresponding free energy profiles for hydride transfer from polyamine to FAD along RC2 obtained on the basis of last 5 ps QM/MM MD simulations. 23

Fig. S18. The changes of bond length for N5'-H1 and C4-H1 along with the RC2 in the last 5 ps QM/MM MD simulations. 24

Fig. S19. The changes of charge for FAD and N^1-Acetylspermine along with the RC2 in the last 5 ps QM/MM MD simulations. 25

Fig. S20. The structure of reactant, transition state, and intermediate together with corresponding free energy profiles for water attacking to polyamine along RC4 obtained on the basis of last 5 ps QM/MM MD simulations. 26

Fig. S21. The prediction of proton transfer and bond cleavage for the generation of final products. 27

Fig. S22. The free energy profiles of different periods for generation of N-acetyl-3-aminopropannal and spermidine with one terminal amino group protonated obtained by QM/MM MD simulations combined with umbrella sampling. 28

Fig. S23. The free energy profiles of different periods for generation of N-acetyl-3-aminopropannal and spermidine with two terminal amino group protonated obtained by QM/MM MD simulations combined with umbrella sampling. 29

Fig. S24. The structure of reactant, transition states, and intermediates together with corresponding free energy profiles for generation of N-acetyl-3-aminopropannal and spermidine with one terminal amino group protonated along RC5 obtained on the basis of last 5 ps QM/MM MD simulations. 30
Fig. S25. The structure of reactant, transition states, and intermediates together with corresponding free energy profiles for proton transfer from His64 to polyamine along RC6 obtained on the basis of last 5 ps QM/MM MD simulations. 31
Fig. S26. The structure of reactant, transition states, and intermediates together with corresponding free energy profiles for generation of N-acetyl-3-aminopropannal and spermidine with two terminal amino group protonated along RC7 obtained on the basis of last 5 ps QM/MM MD simulations. The different between Fig. S26 and the Fig. 3g mainly because two free energy profiles along RC6 and RC7 are merged. In the Fig. S26, the IM4b is start point, and the data of different periods is zero, and in Fig. 3g, the IM4b is calculated corresponded to the terminal of RC6 for three sets of data for 15 - 20, 15 - 19, and 16 - 20 ps that are -5.19681, -5.22666, and -5.28190 kcal/mol, respectively. 32
Fig. S27. The structure of TS3b and TS3b' for proton transfer from His64 to polyamine obtained on the basis of last 5 ps QM/MM MD simulations. 33
Fig. S28. The free energy profiles of different periods for proton transfer and bond broken along RC8 obtained on the basis of QM/MM MD simulations combined with umbrella sampling. 34
Fig. S29. The structure of reactant, transition state, intermediate, and product together with corresponding free energy profile for proton transfer and bond broken along RC8 obtained on the basis of 5 ps QM/MM MD simulations. 35
Fig. S30. The static binding mode of products SP1 and SP2 (SP3) in the active site of APAO obtained by MM MD simulations. 36
Fig. S31. The contribution for each residue and binding free energy decomposition for key residues for SP1 and SP2 in protein-products complex. 37
Fig. S32. The binding free energy decomposition for key residues for SP1 and SP3 in protein-products complex. 38
Fig. S33. The possible release channels and corresponding possibilities for two sets of products obtained by RAMD MD simulations. 39
Fig. S34. The transportation coordinates for SP1 and SP3 in QM/MM MD simulations combined with umbrella sampling. 40
Fig. S35. The 2D free energy profiles obtained by QM/MM MD simulations combined with umbrella sampling for two products release from APAO in different time period. 41
Fig. S36. The represent snapshot of the windows for release of N-acetyl-3-aminopropannal (SP1) and spermidine with two terminal amino group protonated (SP3) obtained by the cluster analysis in the last 5 ns of MM MD simulations. 42
Fig. S37. The bridge of microcosm and macrocosm by catalytic-related network of APAO 43
Fig. S38. The QM/MM model building. 44
Table S1. The location, number, and possibility of trajectories for N^1-acetylspermine delivery pathways based on RAMD MD simulations. 45
Table S2. Individual energies terms of MM/GBSA results for the N^1-acetylspermine-APAO system. (All data units are kcal/mol) 46
Table S3. The barrier heights with zero-point energy (ZPE) corrections, Wigner correction factor, and active energy barrier calculated by Arrhenius equation with three parameters 47
Table S4. Individual energies terms of MM/GBSA results for the N-acetyl-3-aminopropannal and spermidine with one terminal amino group protonated system. (All data units are kcal/mol) 48
Table S5. Individual energies terms of MM/GBSA results for the N-acetyl-3-aminopropannal and spermidine with two terminal amino group protonated system. (All data units are kcal/mol) 49
Table S6. The location, number, and possibility of trajectories for N-acetyl-3-aminopropannal (SP1) and spermidine with one terminal amino group protonated (SP2) delivery pathways based on RAMD MD simulations. 50
Table S7. The location, number, and possibility of trajectories for N-acetyl-3-aminopropannal (SP1) and spermidine with two terminal amino group protonated (SP3) delivery pathways based on RAMD MD simulations. 51
Appendix 52

S1. Convergence of PMF curves

S1.1 N^1-acetylspermine transportation by MM MD simulations. The 1D umbrella sampling simulations are carried out based on TC1 (Figure S1) for N^1-acetylspermine transportation. TC1 is defined as the line of Cα@Asn313 and C11@N1-acetylspermine. For the N^1-acetylspermine transfer, 29 windows with 217,500 snapshots are obtained. As shown in Fig. S2, the free energy profiles are calculated for the periods of 0 ~ 5, 5 ~ 10 and 10 ~ 15 ns. The curves of 5 ~ 10 and 10 ~ 15 ns is very close in shape and energy and the maximal SD for the two periods is 1.72 kcal/mol along the whole transportation coordinate, and the proportion less than 1 is 85.3%. It means the free energy gradually close to convergence after 5 ns. Moreover, the cross-stage verification is performed by using the snapshots in 10 ~ 14, 10 ~ 15, 11 ~ 15 ns, and maximal SD along the reaction coordinate is 0.63 kcal/mol. The PMF curves of the three periods are almost overlapped, and the maximal and SD of the barrier are 23.58 and 0.55 kcal/mol. which means that the PMF curves reach good convergence (see Fig. 2b).

S1.2 FADH and hydroxylated intermediate formation by 2D QM/MM MD simulations. The $FADH^-$ and alcohol intermediate generation involves hydride transfer from N^1-acetylspermine to FAD, water attacking, and proton-shuffle by His64. This process has three reaction coordinates that include $d_{N5'-H1}$-d_{C4-H1}, $d_{Owat1-C4}$, and $d_{N\varepsilon-H3}$-$d_{H3-Owat1}$, named RC2, RC3, and RC4, respectively. The RC3 and RC4 reaction coordinates have a dependent relationship. Thus, the two-dimension free energy profile is acquired based on RC2 and RC4 using QM/MM MD simulations combined with umbrella sampling, and 40 windows with 800,000 snapshots are obtained. The snapshots of 0 ~ 5, 5 ~ 10, 10 ~ 15, and 15 ~ 20 ps are sampled to obtain the 2D free energy profile. As shown in Fig. S12, the free energy distribution is similar for 10 ~ 15 and 15 ~ 20 ps. The maximal SD is 1.24 kcal/mol among 1392 grid points, and only 9 grid points are larger 1. For the barrier of R to IM1, the average and SD are 19.68 and 0.09 kcal/mol, respectively. The energy of TS2 compared with R is 9.98 ± 0.07 kcal/mol. It means that the free energy profile is gradually convergence after 10 ps. Besides that, the free energy profiles obtained based on the periods of 15 ~ 19, 16 ~ 20, 15 ~ 20 ps are further used to verify, and the maximal SD is 0.74 kcal/mol. The average value and SD for the barrier of R to IM1 is 19.54 and 0.08 kcal/mol. The energy of TS2 compared with R is 9.89 ± 0.17 kcal/mol. The mentioned above proves that the free energy profile reach good convergence. Therefore, the snapshots of the last 5 ps are applied for the structure analysis.

S1.3 FADH and hydroxylated intermediate formation by 1D QM/MM MD simulations. The 1D QM/MM MD simulations are carried out based on RC2 and RC4 for formation of FADH and hydroxylated intermediate. For the hydride transfer, 16 windows with 320,000 snapshots are obtained. As shown in Fig. S16a and S17, the free energy profiles are calculated for the periods of 0 ~ 5, 5 ~ 10, 10 ~ 15, and 15 ~ 20 ps. The shape and overall energy are similar for the four periods. The maximal SD for the latter two periods is 1.23 kcal/mol, and the proportion less than 1 is 91%. The SD for the free energy barrier is 0.51 kcal/mol. It means the free energy gradually close to convergence after 10 ps. Moreover, the cross-stage verification is performed by using the snapshots in 15 ~ 19, 16 ~ 20, 15 ~ 20 ps, and maximal SD along the reaction coordinate is 0.15 kcal/mol. The average and SD of the barrier are 22.95 and 0.08 kcal/mol (Figure S17). It means the free energy profile arrive at convergence. As to water attacking by assistance of His64, and 11 windows with 220, 000 snapshots are acquired. The free energy profiles for the periods of 0 ~ 5, 5 ~ 10, 10 ~ 15, and 15 ~ 20 ps are obtained. As shown in Fig. S16b, the shape and overall energy are similar for the latter three period. The maximal SD along the whole reaction coordinate for 10 ~ 15 and 15 ~ 20 ps is 0.43 kcal/mol, and for the barrier, the SD is 0.13 kal/mol. It proves that the free energy profile reaches good convergence after 10 ps. In addition, the free energy profiles of 15 ~ 19, 16 ~ 20, 15 ~ 20 ps are obtained. The maximal SD is 0.19 kcal/mol, and the barrier SD is 0.15 kcal/mol (Figure S20). It also verifies that the good convergence of free energy. Therefore, the snapshots for the last 5 ps are used for the following analysis.

S1.4 The proton transfer from hydroxyl group to N5 of hydrolysed N^1-acetylspermine. The lengthening of C4-N5 and shortening of N5-H4 is defined as reaction coordinate (RC5), and 500,000 snapshots for 25 windows are obtained by using QM/MM MD simulations to determine if the hydroxyl group attracted by the amino group more than His64 in N^1-acetylspermine. As listed in Fig. S22, the free energy are calculated in for the periods of 0 ~ 5, 5 ~ 10, 10 ~ 15, and 15 ~ 20 ps, and the profiles of latter three periods agrees

with each other with the maximal SD of 0.89 kcal/mol. The maximal SD along the whole reaction coordinate for 10 ~ 15 and 15 ~ 20 ps is 0.97 kcal/mol. The SD of the two barriers are 0.61 and 0.75 kcal/mol. It means that the free energy closes to convergence after 5 ps. In addition, the free energy profiles obtained based on the periods of 15 ~ 19, 16 ~ 20, 15 ~ 20 ps are further used to verify (see Fig. S24). The maximal SD is 0.36 kcal/mol, and the SD of the two free energy barriers are 0.10 and 0.16 kcal/mol. It can be seen that the free energy reaches the convergence. The snapshots for the last 5 ps are applied for the structure analysis.

S1.5 The proton transfer from His64 to N5 of hydrolysed N^1-acetylspermine. The lengthening of Nε-H3 and shortening of N5-H3 is selected as reaction coordinate (RC6), and 340,000 snapshots for 17 windows obtained by QM/MM MD simulations are to determine whether His64 attracted by the amino group more than the hydroxyl group. The free energy profiles are acquired for four periods of 0 ~ 5, 5 ~ 10, 10 ~ 15, and 15 ~ 20 ps, which is shown in Fig. S23. It can be seen clearly that the shape and overall energy are overlap well for 10 ~ 15 and 15 ~ 20 ps with the maximal SD of 0.24 kcal/mol. The average and SD for the barrier is 4.74 and 0.05 kcal/mol, respectively. It means that the free energy arrives at good convergence after 10 ps. Moreover, the cross-stage verification is performed for the periods of 15 ~ 19, 16 ~ 20, 15 ~ 20 ps. The maximal SD is 0.10 kcal/mol, and the SD of barrier is 0.06 (see Fig. 4a). It is also verifies the convergence of the free energy profile. As mentioned above, the snapshots for the last 5 ps are used for the following analysis.

S1.6 The formation of N-acetyl-3-aminopropannal and spermidine with two terminal amino group protonated. It should be noted that if the proton donor is a -OH group on C4, the proton transfer causes the cleavage of C4-N5 to form the last products of spermidine and N-acetyl-3-aminopropanal. However, regarding this mechanism, C4-N5 is lengthened but not cleaved. The final products are not generated. Accordingly, another question of “which mechanism will win ultimately?” is likely to emerge. Both Nε of His64 and the $-NH_2^+$- group probably accept the proton of the -OH group on C4. Therefore, the lengthening of C4-N5 and shortening of N5-H4 and Nε-H4 are chosen as reaction coordinates (RC7) to obtain the free energy profile. The 20 ps QM/MM MD simulations are carried out for the generation of N-acetyl-3-aminopropannal and spermidine with two terminal amino group protonated. The 17 windows and 340,000 snapshots are obtained for the free energy calculations. Four periods of 0 ~ 5, 5 ~ 10, 10 ~ 15, and 15 ~ 20 ps are used to estimate the convergence. It can be seen in Fig. S23, the maximal SD for free energy profiles of 10 ~ 15 and 15 ~ 20 ps is 0.88 kcal/mol, and the shape of the free energy profiles seems similar. The SD of the two barriers are 0.26 and 0.15 kcal/mol. It means that it reaches the convergence after 10 ps. The three periods of 15 ~ 19, 16 ~ 20, 15 ~ 20 ps are further to verify, and the maximal SD is 0.11 kcal/mol. It proves that the free energy reaches good convergence. Therefore, the snapshots of the last 5 ps are used for the structure analysis.

S1.7 The test of stepwise reaction coordinate for product generation. To further verify if there is an intermediate of the proton transfer from hydroxyl group to His64, the shortening of Nε-H4 and lengthening of C4-N5 is used as reaction coordinate (RC8). The 20 ps QM/MM MD simulations are carried out, and 20 windows with 400,000 snapshots are obtained. As shown in Fig. S28-S29, the free energy profiles are acquired for the periods of 0 ~ 5, 5 ~ 10, 10 ~ 15, and 15 ~ 20 ps, and it closes to convergence after 5 ps. The maximal SD for 10 ~ 15 and 15 ~ 20 ps is 1.17, and the proportion for SD larger than 1 is 7%. The free energy is up and up, and the average and SD of highest point is 17.38 and 0.57 kcal/mol. Besides that, the three periods of 15 ~ 19, 16 ~ 20, 15 ~ 20 ps are further to verify. The maximal SD is 0.23, and the highest point is 16.97 ± 0.22 kcal/mol. It means that the free energy reaches convergence.

S1.8 The two products escape from the APAO. As to the most favorable channels, the N-acetyl-3-aminopropanal (SP1) and spermidine with two protonated terminal amino group (SP3) delivery from the active site to the outside of the APAO is calculated on the basis of MM MD simulations combined with umbrella sampling along the TC2 and TC3 (Figure S34). TC2 is the line of Cα@Ile358 and C4@SP1. TC3 is the line of Cα@Ser431 and C1@SP3. 288 windows and 2,880,000 snapshots are obtained. As is shown in Figure S35, the free energy profiles of the periods of 0 ~ 4, 4 ~ 8, 8 ~ 12, 12 ~ 16, and 16 ~ 20 ns are obtained, which gradually reach convergence after 12 ns. The maximal SD for 12 ~ 16 and 16 ~ 20 ns is 1.05, and the proportion for SD less than 1 is 99.95%. Besides that, the three periods of 15 ~ 19, 16 ~ 20, 15 ~ 20 ns are further to verify. The maximal SD is 0.63, which means that the profile arrives at convergence. The last 5 ns are used for the detail mechanism analysis.

S2. The detail delivery process of reactant in second stage

The ligand gradually enters the cavity and when the ligand moves to at 31.5 Å, the amino group of the ligand forms a strong electrostatic interaction with the carbonyl group on the Asp211 side chain. As the ligand moves, further new hydrogen bonds are formed with Asp211 and also with Asn91, and the unconventional hydrogen bond interaction of Ala101 drives the ligand into the cavity. the ligand slowly moves toward the catalytic site as the interaction increases with further movement of the substrate. When the substrate moves near 29.5 Å, Trp62 is involved in substrate transport via π-cation interactions. Subsequently, the substrate continued to move toward the catalytic site guided by Asp211, and at 26.5 Å, Phe331 formed a π-cation interaction with the substrate. As the substrate gradually approaches the pocket, the number of hydrogen bonds formed increases, enhancing the interaction between N^1-acetylspermidine and APAO. The side chain conformation of Asp211 changes, consistently directing the ligand toward the center of the catalytic site, Asp211 plays an important role in substrate recognition and guidance, showing a general pattern of polyamine molecules being recognized. At 24.5 Å, Glu184 forms an electrostatic interaction with the substrate to drive its movement. Subsequently, the substrate comes near 21.5 Å and the cofactor FAD is involved in the substrate transport process through an unconventional hydrogen bond. When the reaction coordinates come to 20.5 Å, the polyamine chain has partially entered the active site, hydrogen bonding interactions increase, and His64 and Val187 form a hydrogen bond with the ligand, respectively, and the formation of these hydrogen bonds can help acetyl spermine find a suitable recognition channel. In addition, Gln330 forms a hydrogen bond with it at the tail of the ligand, providing a driving force for the movement of the ligand at this stage. Meanwhile, His61 and cofactor FAD have hydrogen bond interactions with the ligand and these play an important role in the recognition process of the ligand. As the ligand moves, its long chain conformation is further adjusted under the influence of these interactions. The details are listed in Fig. S3.

S3. The reaction coordinate selection for formation of FADH and hydroxylated intermediate

In QM/MM MD simulations, the model is firstly optimized by using interactive minimization procedure on basis of stable snapshot in MM MD simulations, and then 10 ps QM/MM MD simulations are performed to relax it without any constrain. The snapshots show that the water molecular that may attack N^1-acetylspermine is not stable, and the distance between the Owat1 and C4 of polyamine fluctuates from 3.4 to 6.2 Å. Here we speculate that if intermediate completed water attacking as the starting point, the C4-Owat1 bond forms that probably stable compared with free water molecular, and thus the most favorable reactant and water location will be confirmed from the intermediate that may avoid the error. Hence the minimum reaction energy path (MEP) is mapped by employing reaction coordinate driving (RCD) method starting from reactant to ensure the intermediate. The hydride transferring between FAD and N^1-acetylspermine and water attacking to N^1-acetylspermine involves distance shortening of Owat1-C4 and H1-N5′, and thus the reaction coordinate is defined as - $d_{Owat1\text{-}C4}$ - $d_{H1\text{-}N5'}$ (RC1). The relative energy shows that two intermediates are observed, and the IM2-test1 has lower energy (see Fig. S7). Here the reaction coordinate driving does not involve relax umbrella sampling, the RC1 is the initial test reaction coordinate to find the stable intermediate, and the stable binding point may be not the most reactive stable point. The two reasons may cause the high relative energy barrier. While if the intermediate is more stable, it is useful for identify the favorable reactant conformation. Then the 10 ps QM/MM MD simulations are performed on them to further verify. It can be seen that the IM2-test1 is definitely stable with slight fluctuation from both structure and energy (see Fig. S8). Accordingly, the IM2-test1 is served as the key stable point for obtaining the minimum reaction energy path. Here the hydride will be transferred from FAD back to N^1-acetylspermine, and the hydroxyl will capture the proton of Nε in His64 to make the C4-Owat1 break. Therefore, three sets of reaction coordinate are involved that are $d_{N5'\text{-}H1}$-$d_{C4\text{-}H1}$, $d_{Owat1\text{-}C4}$, and $d_{N\varepsilon\text{-}H3}$-$d_{H3\text{-}Owat1}$, which named as RC2, RC3 and RC4, respectively. If we want to obtain the dependence of three reaction coordinates and relative energy, four-dimensions will be considered that is difficult to dispose, and thus reducing the dimension is a solution. Here we observed that if RC2 is fixed, driving RC3 and RC4, respectively, will bring almost the similar relative energy and the reaction coordinates tendency (see Fig. S9-S10). They have a dependent relationship with one decrease and the other decrease, and thus RC3 and RC4 can be fixed or changed together. Hence three dimensions of RC2, RC3 (or RC4), and relative energy can describe

the sequence of the reaction preliminarily, and two-dimension RCD calculations will be performed. Firstly, we take the energy scanning along RC4, and constraining RC2. The RC3 will be changed accompanied with the reaction coordinate driving. Then on basis of snapshots obtained by it, the energy scanning along RC2 is performed with RC3 and RC4 fixed. Here the RC2 is one dimension, while RC4 is another dimension. As Fig. S11 shows, it may be a stepwise mechanism with hydride transfers from N^1-acetylspermine to FAD preferentially, and then the water attacks the C4 with the proton carried away by His64 to form the tetrahedral intermediate (IM2-scan2d). Besides that, for both reactant-scan and IM1-scan2d, we find that their energies are not focused, which may be related with the unstable water that around His64. As mentioned above, the QM/MM MD calculations combined with umbrella sampling is necessary. Adequate sampling can describe the mechanism more detail, and the potential of mean force is more reliable. Considering the cost of computation and key feature of the two steps, 1D and partial 2D umbrella sampling are performed. The $d_{N21\text{-}H56}$-$d_{C54\text{-}H56}$ (RC2) and $d_{N9\text{-}H73}$-$d_{H73\text{-}O71}$ (RC4) that are related with the hydride transfer between FAD and N^1-acetylspermine and proton transfer between water and His64 are used for the 1D free energy profile calculation, and 27 windows and 540,000 snapshots are collected. The same reaction coordinates as 2D-RCD are applied for the two dimensions free energy profile calculation, and 40 windows and 800,000 snapshots are gathered. Noted that the reactant platform in 2D free energy profile illustrates that the unstable water or small molecules interfered in confirming the reactant state. The relatively more stable intermediate state combined with free energy calculations is benefit for identifying the equilibrium reactant state.

S4. Wigner tunneling correction[1] and calculated level estimation

Because M06-2X has advantages in dealing with weak interactions, and thus M06-2X and B3LYP methods are tested. Moreover, basis set considering dispersion function is useful for disposing system with negative charge. Though the charge of the present QM region is 0, we also tested the basis sets of 6-31G(d) and 6-31+G(d). The coordinate and thermodynamic data of reactant and transition state are obtained at B3LYP/6-31G(d), M06-2X/6-31G(d) and M06-2X/6-31+G(d) levels, respectively. The Cartesian coordinates for these states are listed in Appendix, and the corresponding structures are shown in Fig. S15. The barrier heights with zero-point energy (ZPE) corrections, Wigner correction factor,[2] and active energy barrier calculated by Arrhenius equation with three parameters on the basis of TST and TST/W are listed in Table S3. It can be seen that the Wigner correction factor are 1.24 ~ 1.74. The tunneling correction will reduce the energy barrier by 0.37 ~ 0.56 kcal/mol. It means that the tunneling effect is unapparent. As to B3LYP and M06-2X, the difference of barrier heights with ZPE corrections for the same basis set is 0.83 kcal/mol. It implies that the effect of barrier by two methods is small. For basis sets of 6-31G(d) and 6-31+G(d), the difference of barrier heights with ZPE corrections for M06-2X method is 1.10 kcal/mol. It implies the effect of barrier by dispersion function is also small. The results of B3LYP/6-31G(d) and M06-2X/6-31+G(d) agree well with each other with 0.27 kcal/mol. It means that the B3LYP/6-31G(d) treat QM region combined with MM MD simulations could obtain reliable results for the present system.

S5. One-dimension free energy profile and mechanism for formation of FADH and hydroxylated intermediate

As to the stepwise mechanism, the hydride transferring from N^1-acetylspermine to FAD is prior to the water attacking to N^1-acetylspermine. As shown in Fig. S17, From R-one to IM1-one, the strength of hydrogen bonds formed by FAD, Wat1, Wat2, and N^1-acetylspermine changes little. The distance between H1 and N5′ significantly reduced from 3.04 ± 0.05 to 1.02 ± 0.04 Å, which means that the hydride is transferring from FAD to N^1-acetylspermine. Here all the charges of atoms in FAD in QM/MM region and H1 along the reaction coordinate are collected, which shows that the electron transfer from N^1-acetylspermine to FAD accompanied by hydrogen transfer, and thus the hydride transfer is a H-shift coupled electron transfer process (see Fig. S18-S19). Accordingly, the electro-positivity of C4 is reinforced, and the electronic cloud of N5 shifts to the C4 that lead the bond length of C4-N5 is obviously shortened from 1.47 ± 0.03 to 1.29 ± 0.03 Å. The Wat3 unsteadily floats between N^1-acetylspermine and His64 with weak force around it. From IM1′ to IM2′ (see Fig. S20), the proton has transferred from Wat1 to His64, and the water completely accomplished its attacking to form C4-Owat1 bond. Therefore, the electronic cloud of hydroxyl group

neutralizes electro-positivity of C4, and thus the bond length of C4-N5 is lengthen from 1.29 ± 0.02 to 1.43 ± 0.03 Å. The mechanism is similar with the result of 2D-PMF profile.

As to the free energy profile, the hydride transfer process needs to overcome the barrier of ~ 22.95 kcal/mol, and the barrier of the following water attacking is ~ 7.82 kcal/mol. The former is much higher than the experimental data, and it also higher than the barrier calculated on the basis of 2D-QM/MM MD sampling by ~ 3.15 - ~ 4.25 kcal/mol. The two-dimension considers more factors that may reduce the error brought by the selection of reaction coordinate to some extent.

S6. The orientation changes of hydroxyl group of N^1-acetylspermine

A hydrogen bond interaction is identified between the hydroxyl group and His64 at IM2 (Fig. 3f), which disturbs the delivery of proton to amino group of polyamine. Hence the hydroxyl group attracts the proton to form a water molecular, and the water molecule forms a water bridge with the other two water molecules. The changes lead to the formation of the two wavelet packets of transition states from -2.0 to -1.6 Å along RC6 of the free energy profile referred to as TS3b and TS3b', respectively. The TS3b' has a higher energy than TS3b. The main difference in this transition state is the hydrogen bond interactions between three water molecules (see Fig. S27). In TS3b, the hydroxyl group has the tendency of departure from C4 to form water molecular. In TS3b', the three water molecules form a water-bridge hydrogen bond interactions, while the state is very unstable, and occasionally, as the reaction proceeds, the hydroxyl group returns to the N^1-acetylspermine, and its orientation is changed to further arrive at IM3b (Fig. 4b).

S7. The competition of Nε of His64 and the -NH_2^+- group of polaymine for proton of hydroxyl group on C4

A transition state platform is observed from -1.5 to -0.5 Å of RC7, which may come from the competition of two receptors. During the period, the proton belongs to His64, and N5-H4 has a strong fluctuation that means the protonated imino group prepares to capture the proton. Next, His64 has the advantage of attracting the -OH group proton and an IM5b without C4-N5 cleavage is identified (Fig. 4d). The free energy goes up after the bonding of Nε-H4, and the transition state is observed from -1.5 to -0.5 Å of the reaction coordinate. During the period, the proton belongs to His64, and N5-H4 has a strong fluctuation that means the protonated imino group prepares to capture the proton. Moreover, the C4-N5 bond is broken, which generates neutral spermidine and N-acetyl-3-aminopropanal, while it is super unstable in the transition state platform. In quick succession, the imino group is predominant for drawing the proton in protonated His64, which further forms the final stable products of protonated spermidine and N-acetyl-3-aminopropanal (Fig. 4e).

S8. The binding mode and binding free energy decomposition for SP1 and SP3 (SP2)

In the active site, the two products are dispersed. SP1 forms a hydrogen bond with Phe375. An attractive charge interaction is formed between Glu184 and SP3. SP3 forms a cation - π interaction with Tyr430 and hydrogen bonding interactions with Val187 and Ser467. In addition, Tyr204 forms a π - σ interaction with the -CH_2- group. SP2 and SP3 share similar bonding characteristics (Fig. S30). The MM/GBSA results show that the binding free energies for SP1 and SP3 are -9.3 and -24.7 kcal/mol respectively. The decomposition of the binding energy indicates that the hotspots of His64, Val187, Ser188, Phe201, Tyr204, Tyr430 and Thr468 anchor SP2 to the protein pocket mainly by van der Waals and non-polar interactions (Fig. 6a and S31-S32 and Table S4-S5), while electrostatic and polar interactions are served as the main binding forces for Glu184 and Ser467. The key residues affecting SP1 binding are Trp62, Ile183, Phe331, Gln333, Ile358, Phe360, Leu361 and Phe375, all of which have van der Waals interactions and non-polar interactions as the main binding drivers. The binding modes of the different protonated systems are similar. In terms of binding free energy, SP3 shows better binding affinity to APAO, which may be caused by additional two hydrogen bonds formed with Ser467 and Val187. For the product molecule SP1, the binding free energy and binding mode show slight changes.

S9. RAMD MD simulations of two sets of products

The release processes for the two catalytic products (SP1 and SP2, SP1 and SP3) from APAO probably influence the enzymatic efficiency in the whole catalysis. Therefore, RAMD combined with MD simulations

are performed to find possible release channels for each est of products. Totally 448 trajectories are obtained for the four molecules. For each molecular, two possible release pathways are identified, which is same with the channels of reactant entrance. They are named as P1 and P2. As shown in Fig. S33, the P1 is enclosed by a circular-like cavity composed with the residues of Glu84 ~ Pro103, Ser365 ~ Ser367, and Pro327 ~ Gln330. The P2 is composed with residues of Iyr204 ~ Pro208, Leu116 ~ Phe136, and Leu93 ~ Pro103 that enclosed by a circular-like cavity. Statistically, the probability of product molecules crossing the two potential release channels is shown in the Table S6-S7. As for the first set, the release probabilities of SP1 from the P1 and P2 are 52.7% and 47.3%, respectively. The release probability of SP2 from P1 and P2 is 41.1% and 58.9%. With respect to the other set (SP1 and SP3), the probabilities of the P1 and P2 for SP1 are 67.0% and 33.0%, and probabilities of the P1 and P2 for SP3 are 20.5% and 79.5%. Accordingly, SP1 tends to release from P1, and SP3 and SP2 is likely to release from P2.

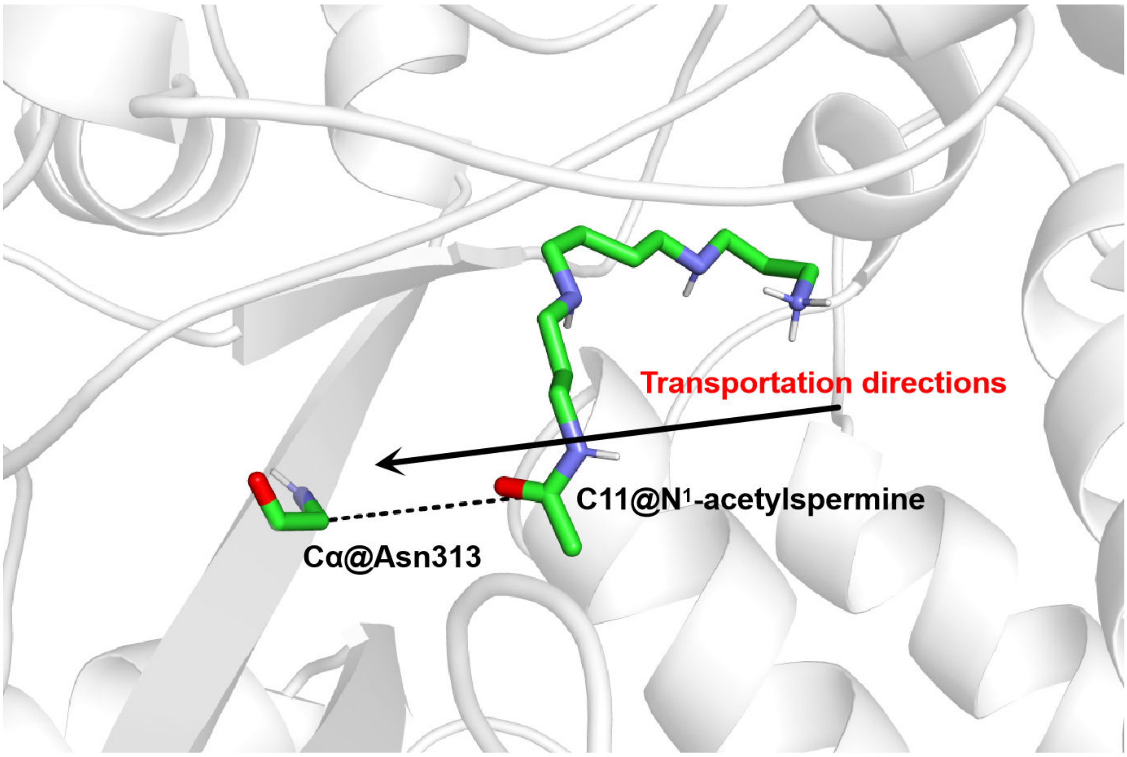

Fig. S1. The TC1 for reactant entrance into the active site of APAO.

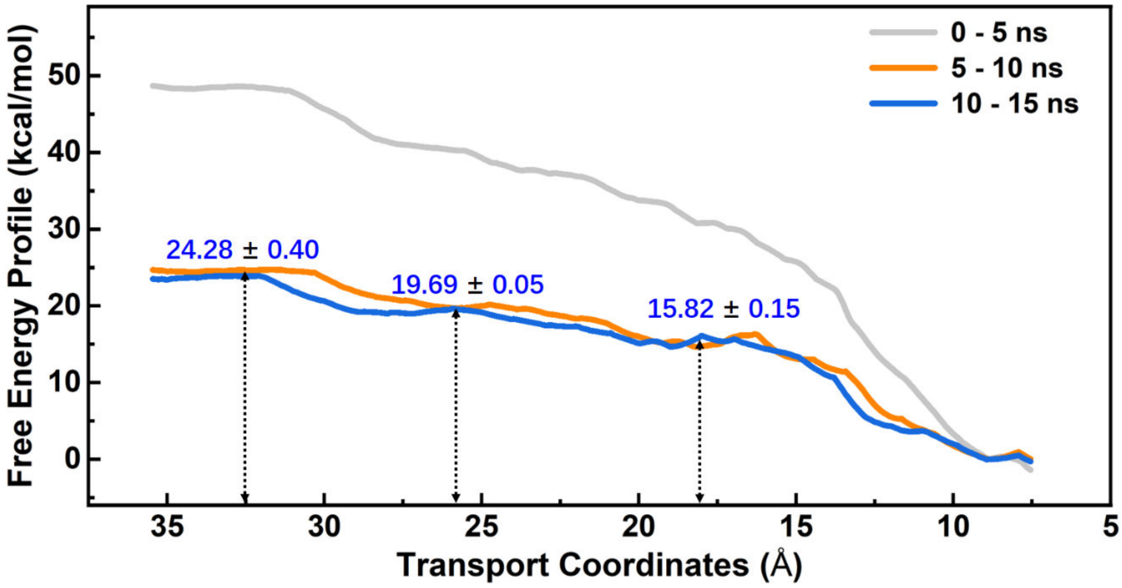

Fig. S2. The free energy profiles obtained for the periods of 0 ~ 5, 5 ~ 10, and 10 ~ 15 ns obtained by MM MD combined umbrella sampling.

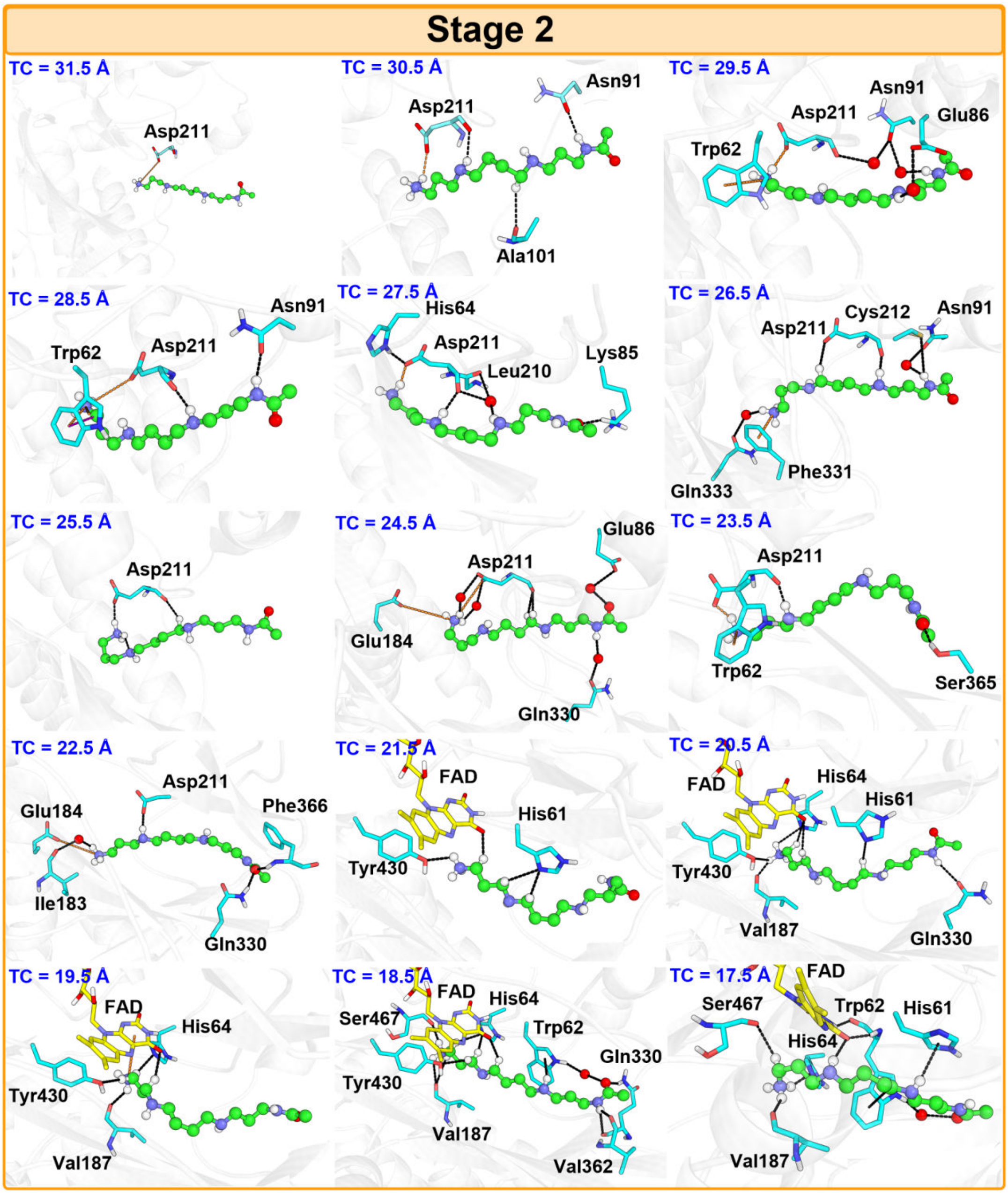

Fig. S3. The represent snapshot of the windows for the second stage obtained by the cluster analysis in the last 5 ns of MM MD simulations.

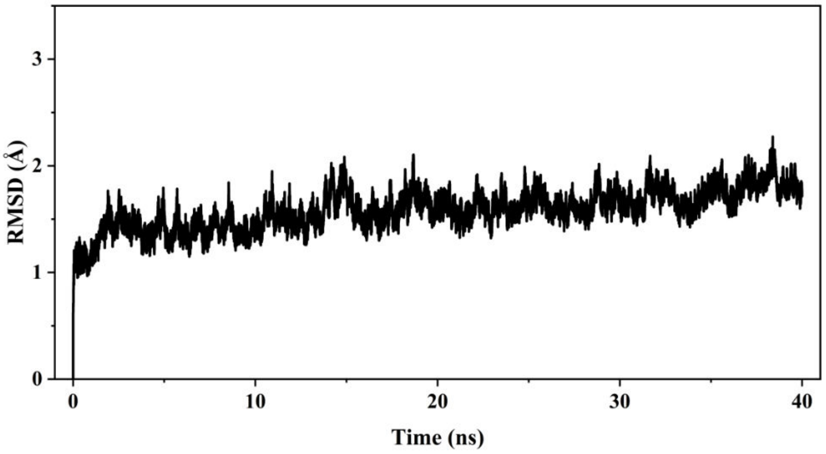

Fig. S4. The RMSD of the APAO in the 40 ns MM MD simulations of the complex system.

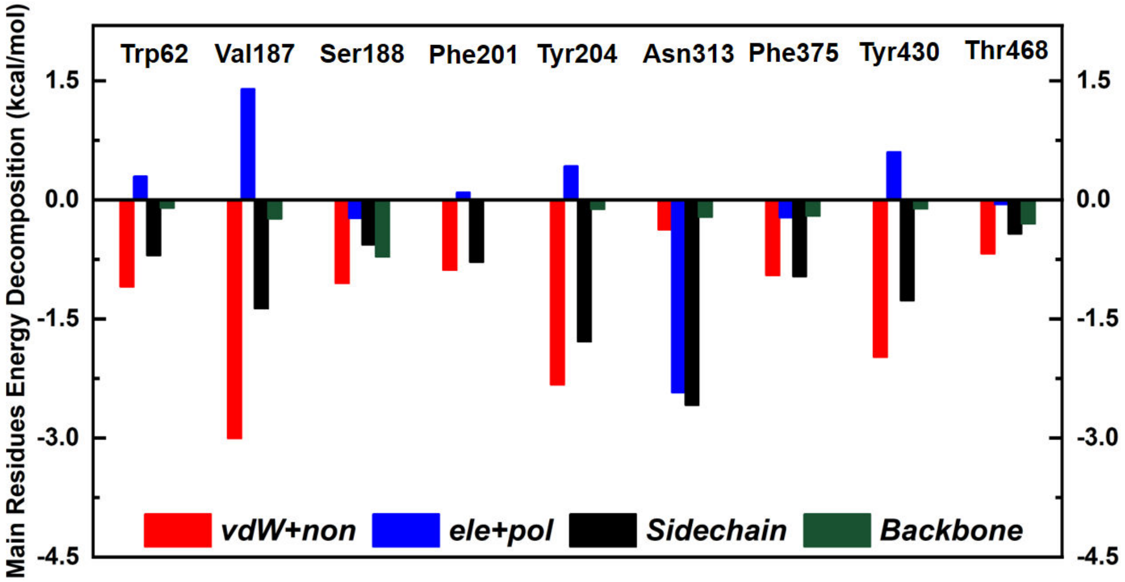

Fig. S5. The free energy decomposition for the key residues in the static binding of APAO and reactant.

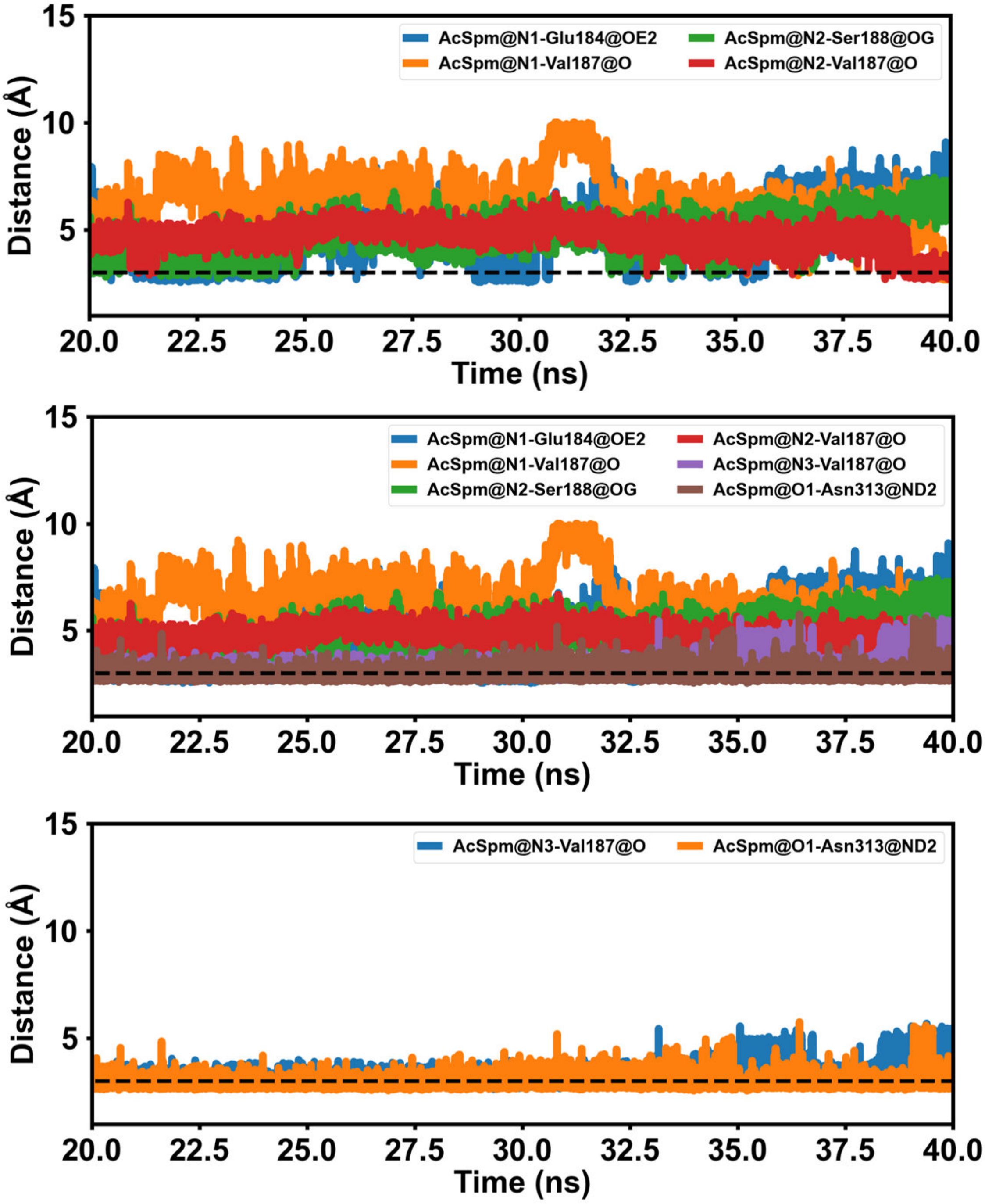

Fig. S6. The stabilization of hydrogen bond interactions in the MM MD simulations of complex system.

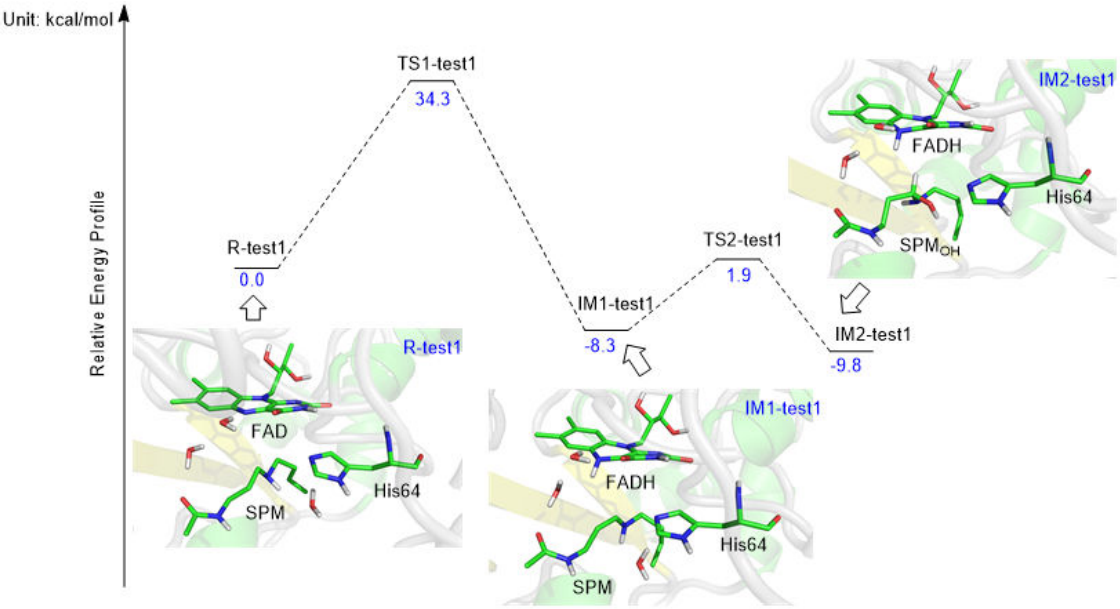

Fig. S7. The reaction coordinate test in the QM/MM RCD simulations on the basis of the stable state obtained in MM MD simulations (The reaction coordinate is defined as - $d_{Owat1\text{-}C4}$ - $d_{H1\text{-}N5'}$ (RC1)).

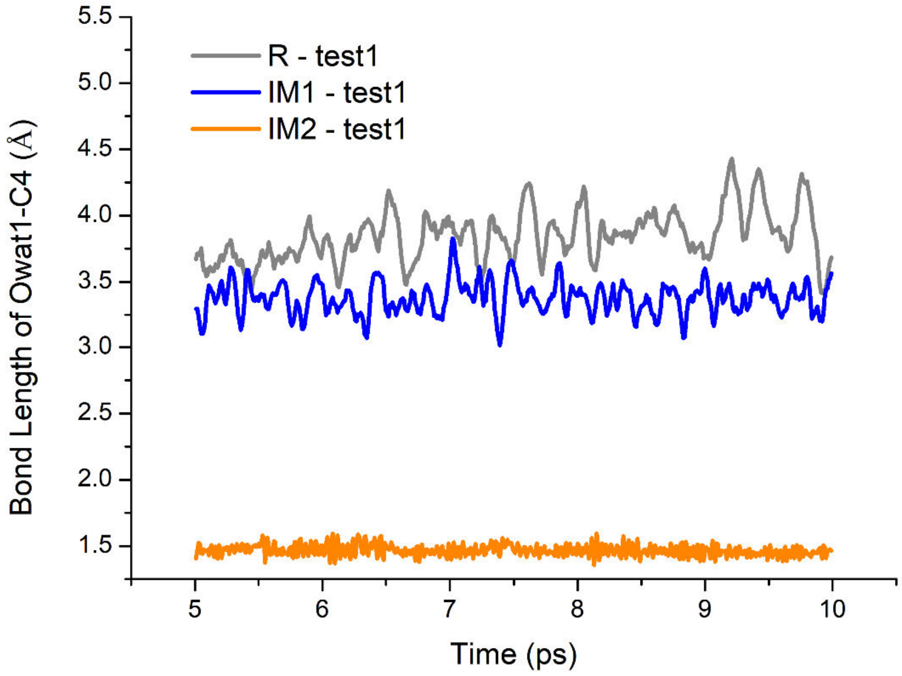

Fig. S8. The comparison of R - test1, IM1 - test1, and IM2 - test1 for the bond length of Owat1-C4 in the 10 ps QM/MM MD simulations.

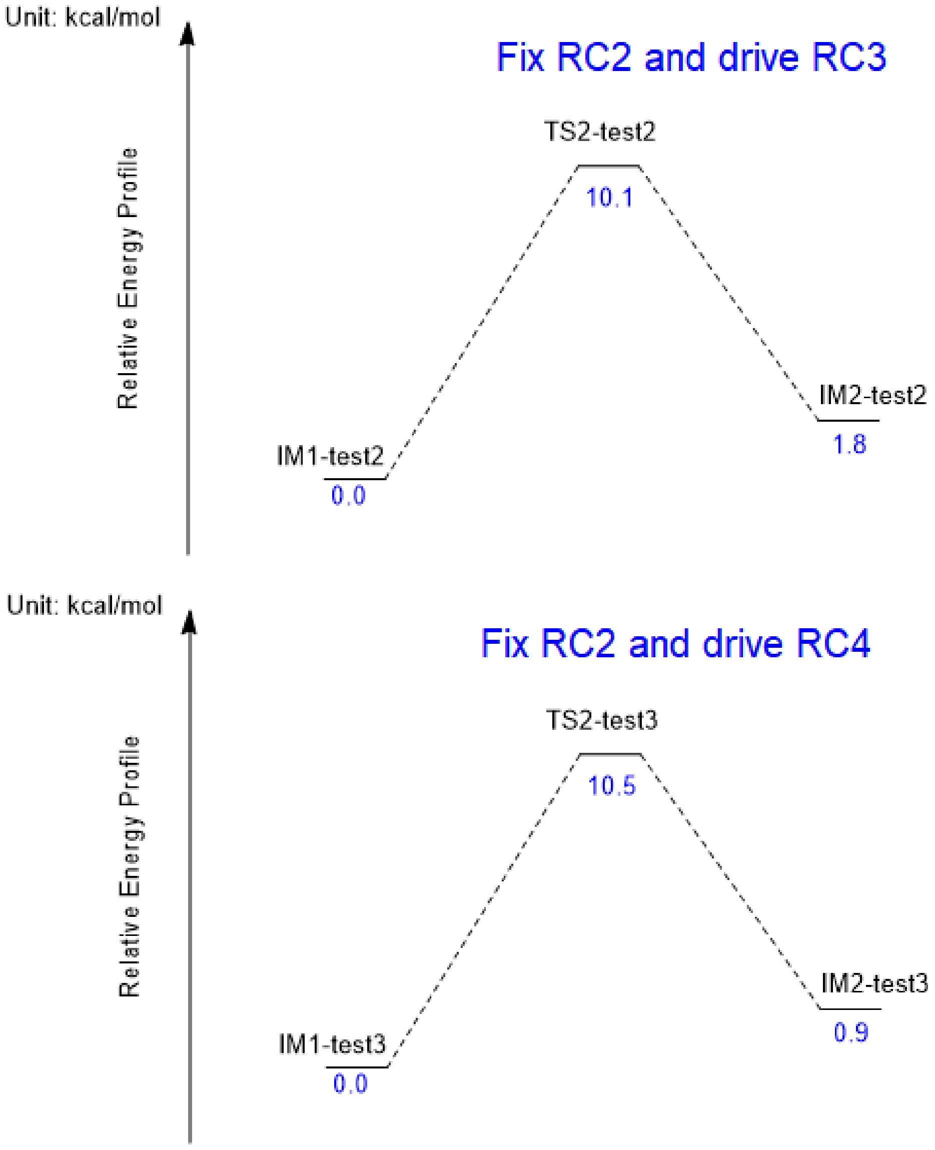

Fig. S9. The relative energy comparison for fixing RC2 and driving RC3 (RC4) in QM/MM RCD simulations.

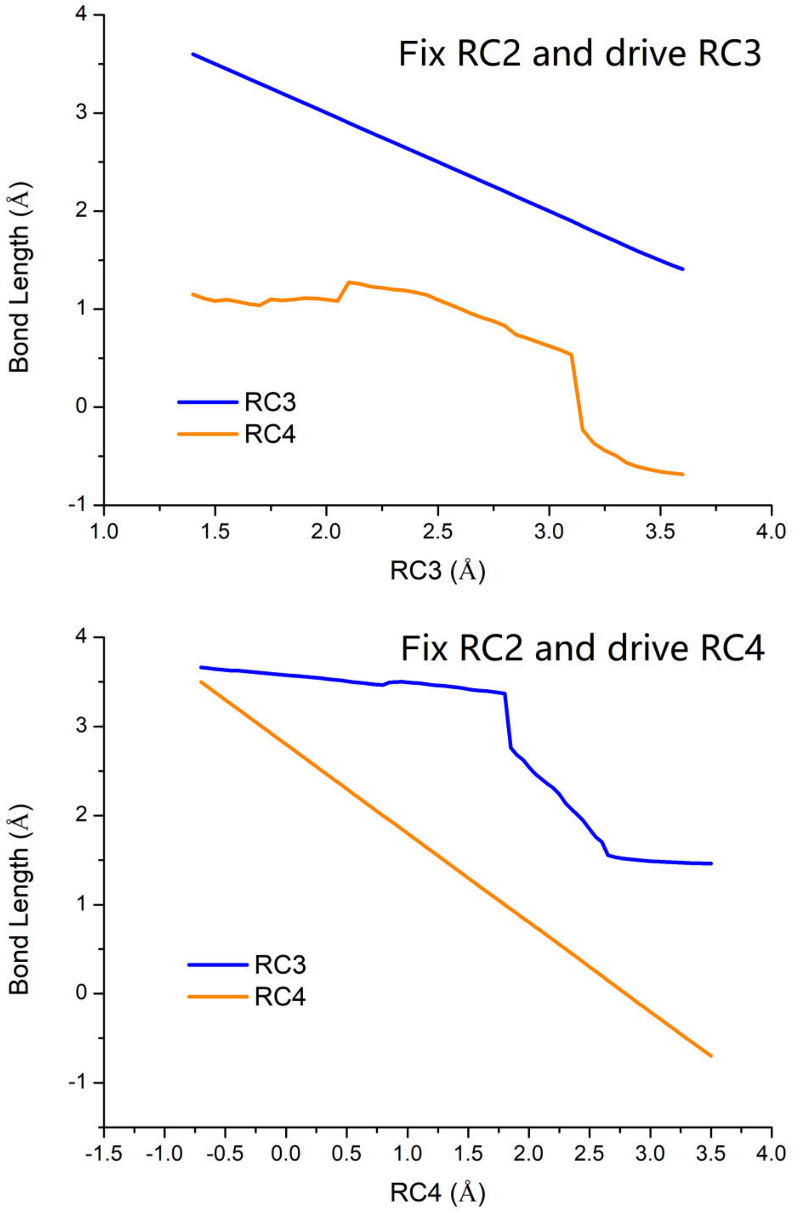

Fig. S10. The changes of bond length for RC3 and RC4 for fixing RC2 and driving RC3 (RC4) in QM/MM RCD simulations.

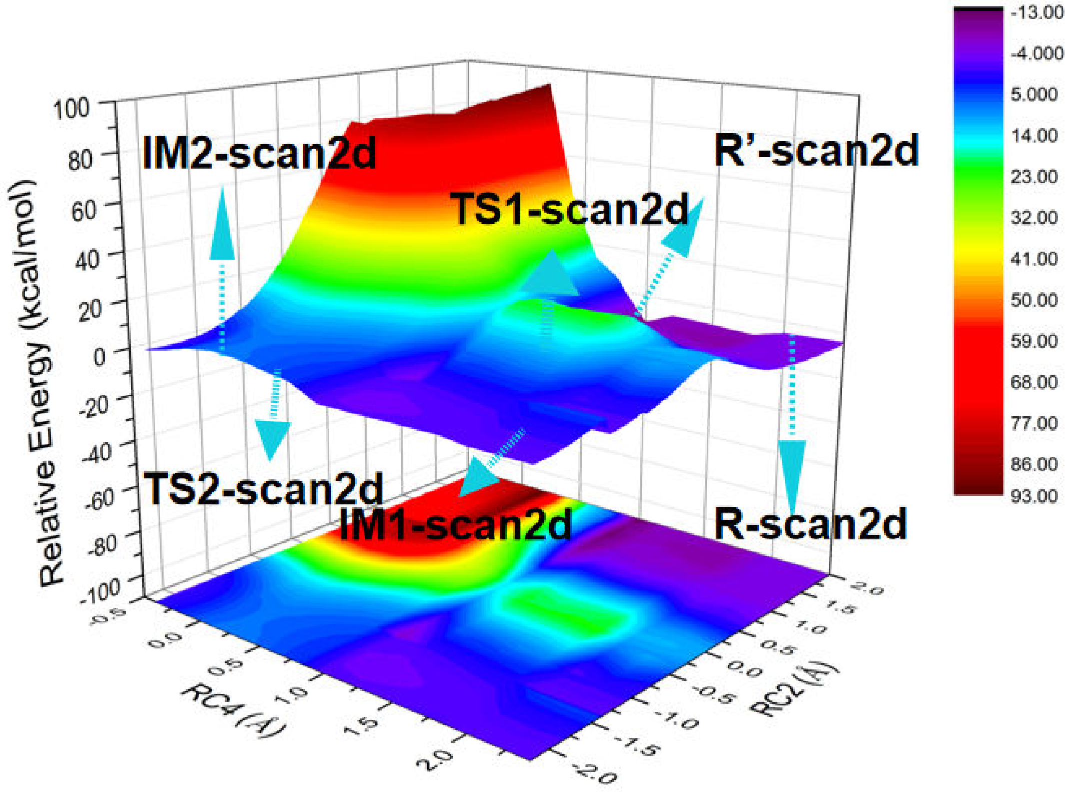

Fig. S11. The relative energy profile obtained by 2D-QMM/MM RCD simulation on the basis of RC2 and RC4.

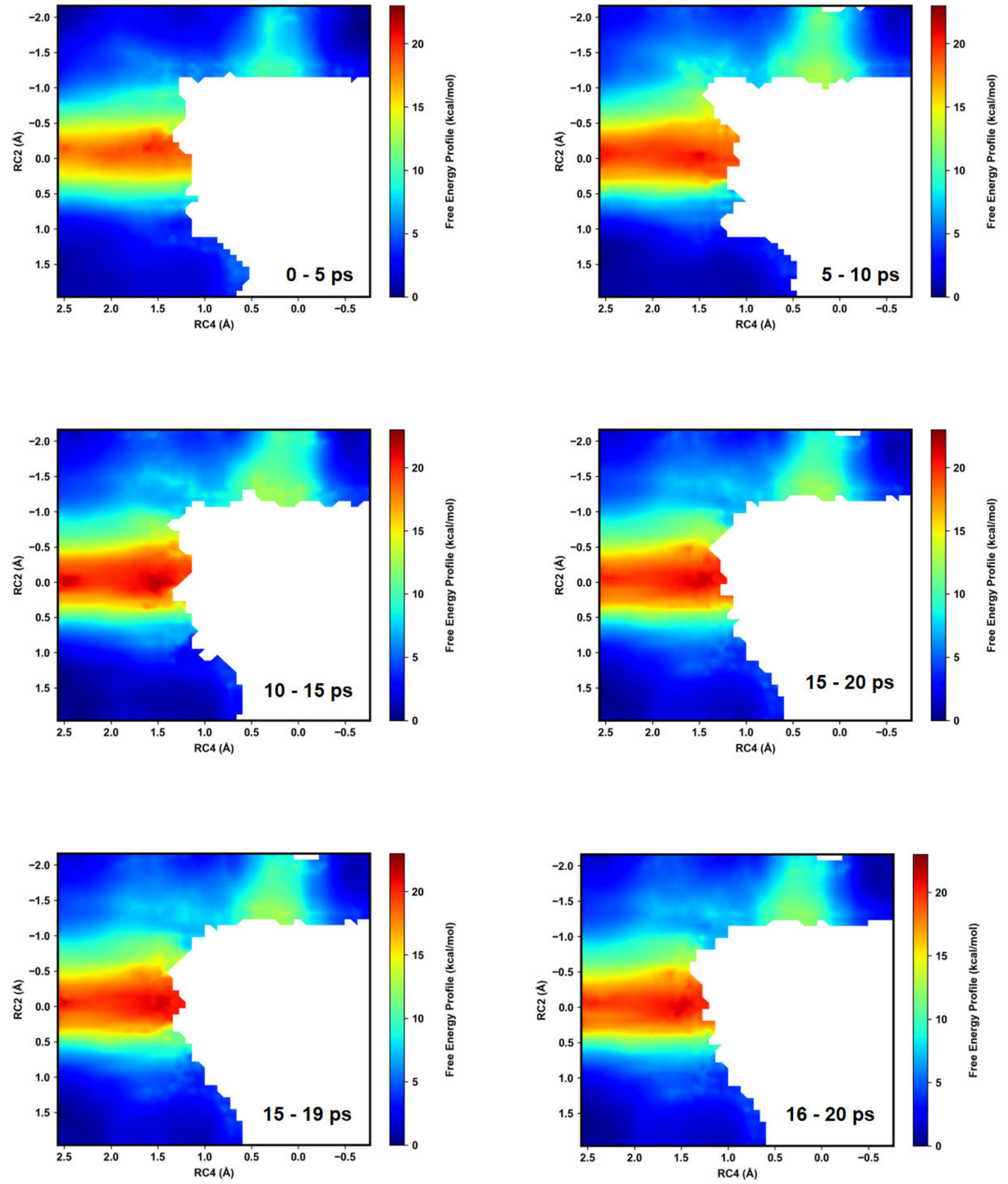

Fig. S12. The free energy profile of different periods for generation of $FADH^-$ and hydroxylated polyamine obtained by QM/MM MD simulations combined with umbrella sampling on the basis of RC2 and RC4.

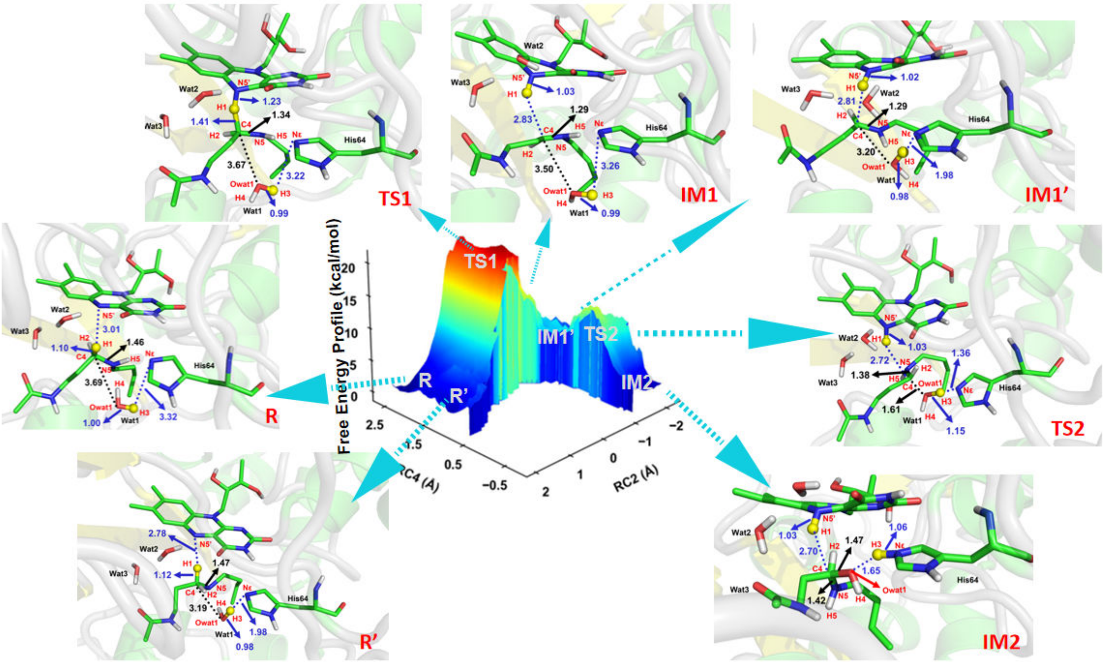

Fig. S13. The reactant, intermediates, and transition states along with free energy profile in generation of FADH⁻ and hydroxylated polyamine obtained by last 5ps QM/MM MD simulations combined with umbrella sampling on the basis of RC2 and RC4.

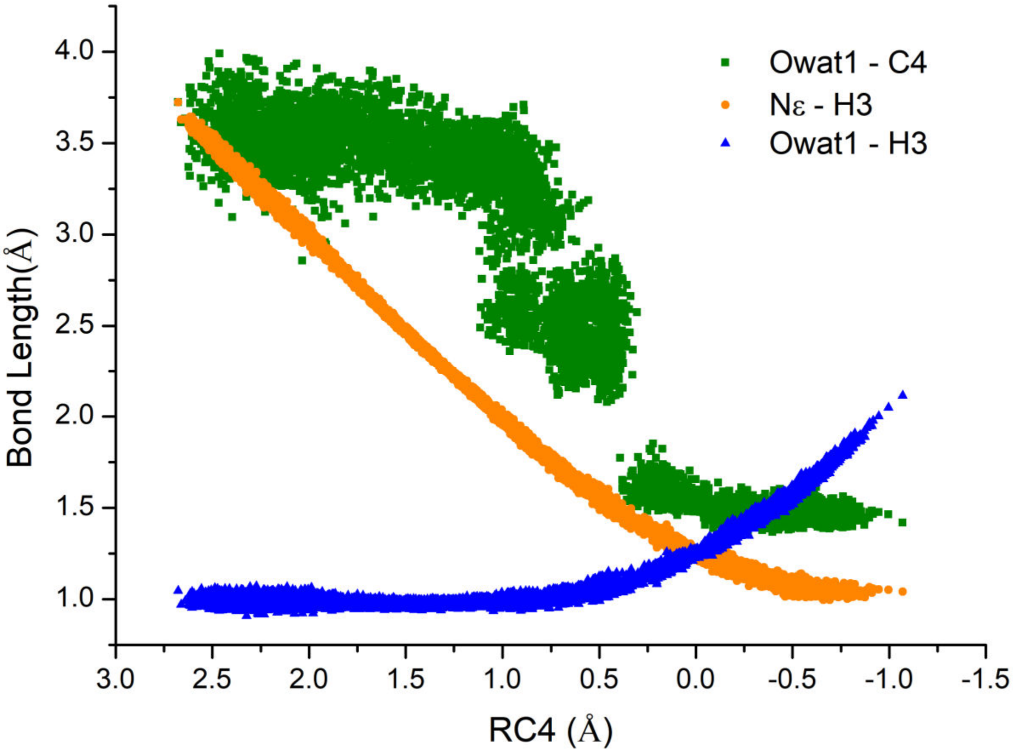

Fig. S14. The bond length changes of Owat1-C4, Nε-H3, and Owat1-H3 along the RC4 in the last 5 ps QM/MM MD simulations.

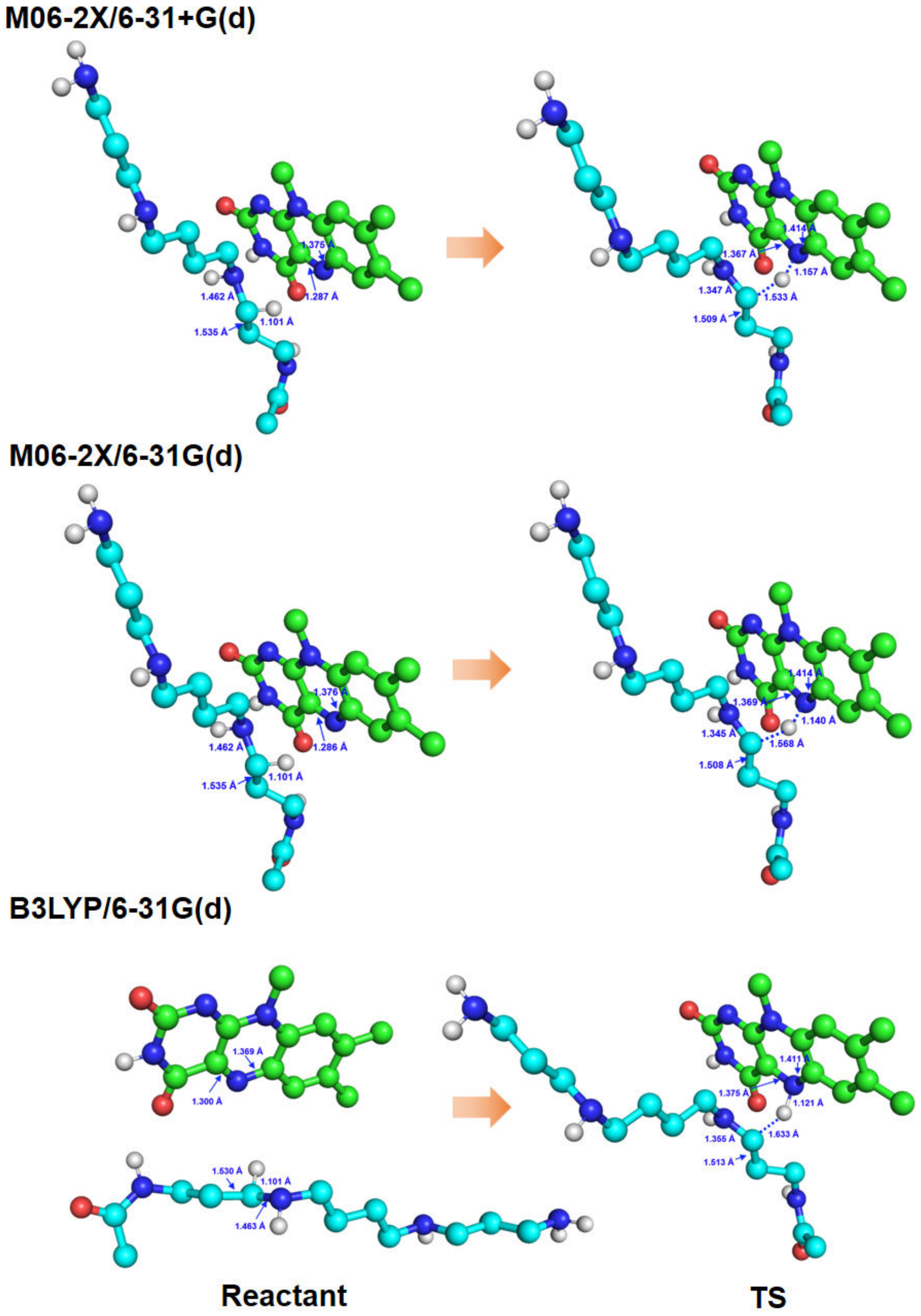

Fig. S15. The structure of reactant and transition state obtained by different DFT methods, and the corresponding Cartesian coordinates is listed in Appendix.

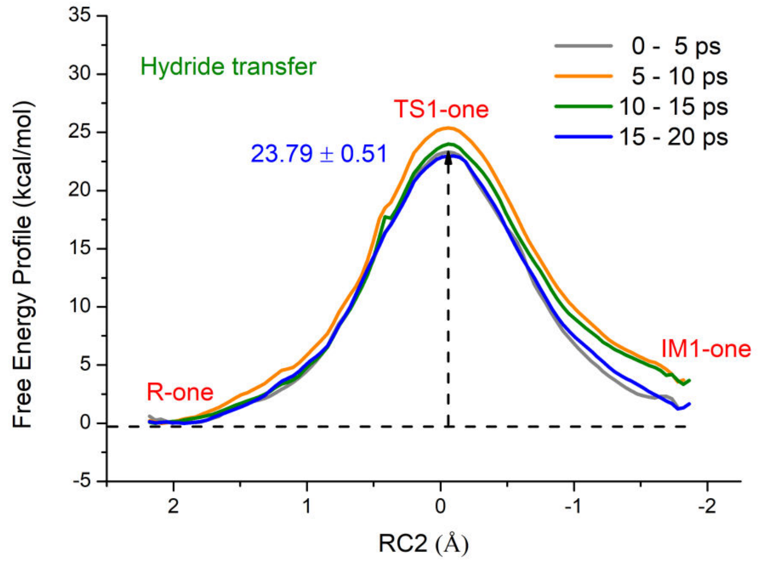

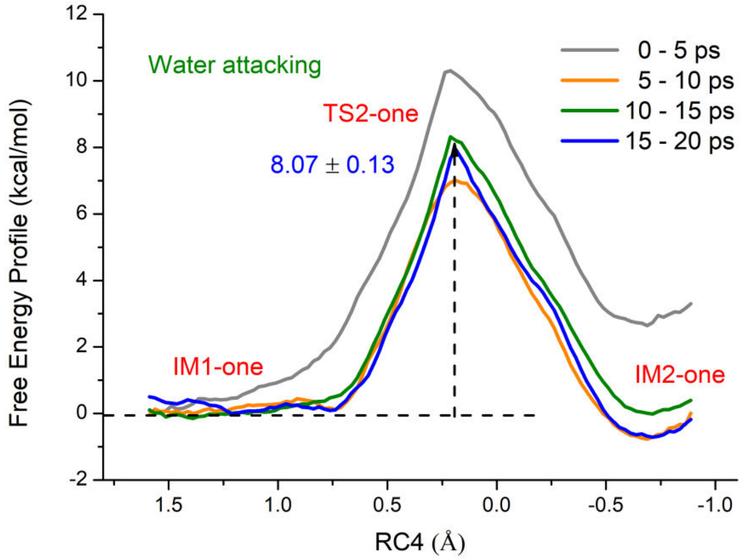

Fig. S16. The free energy profiles of different periods obtained by one dimension QM/MM MD simulations combined with umbrella sampling on the basis of RC2 and RC4.

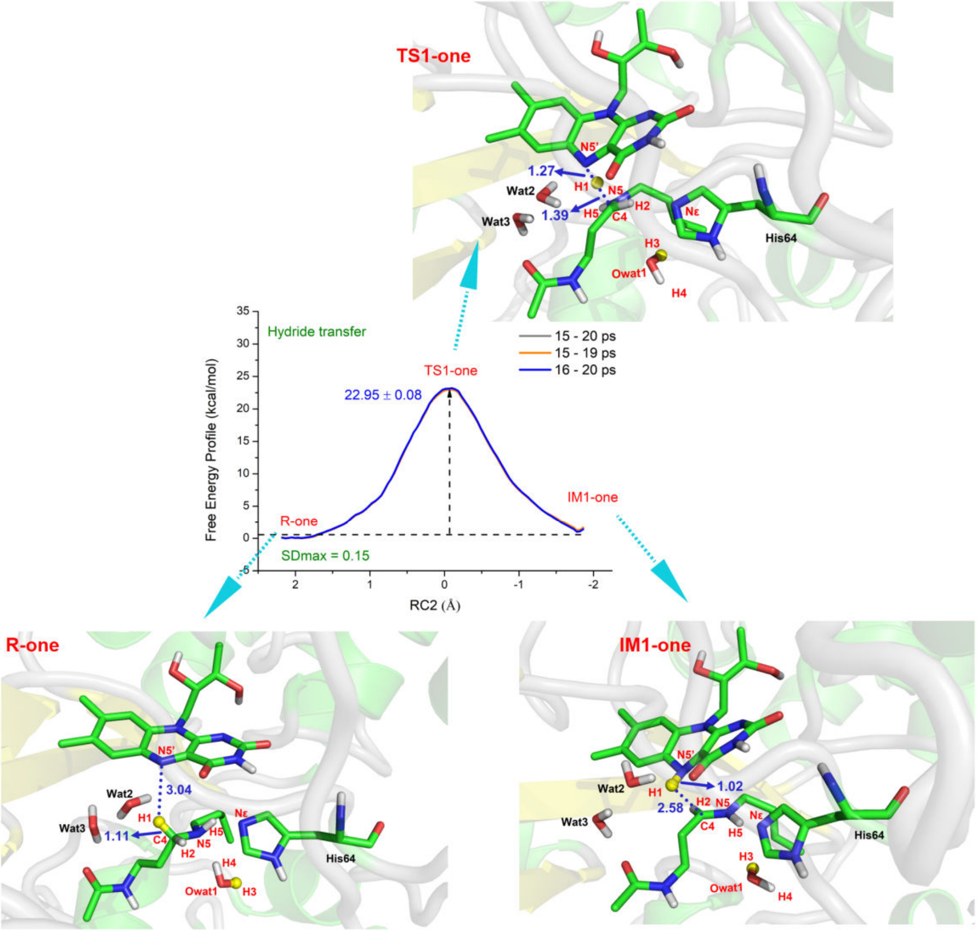

Fig. S17. The structure of reactant, transition state, and intermediate together with corresponding free energy profiles for hydride transfer from polyamine to FAD along RC2 obtained on the basis of last 5 ps QM/MM MD simulations.

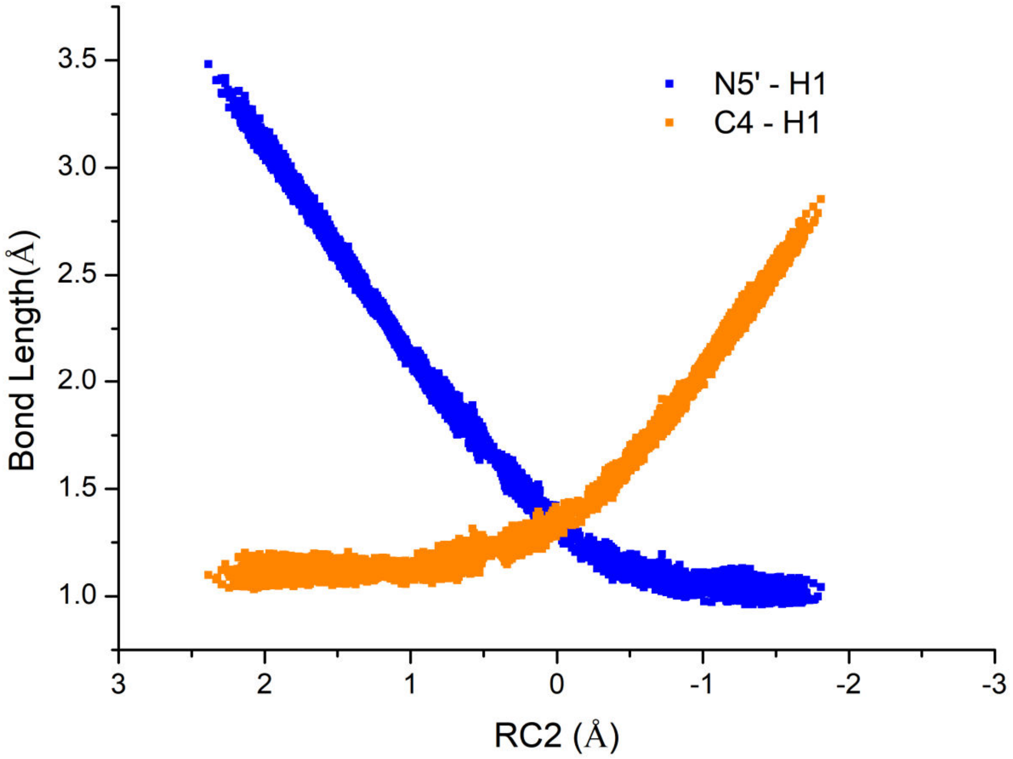

Fig. S18. The changes of bond length for N5'-H1 and C4-H1 along with the RC2 in the last 5 ps QM/MM MD simulations.

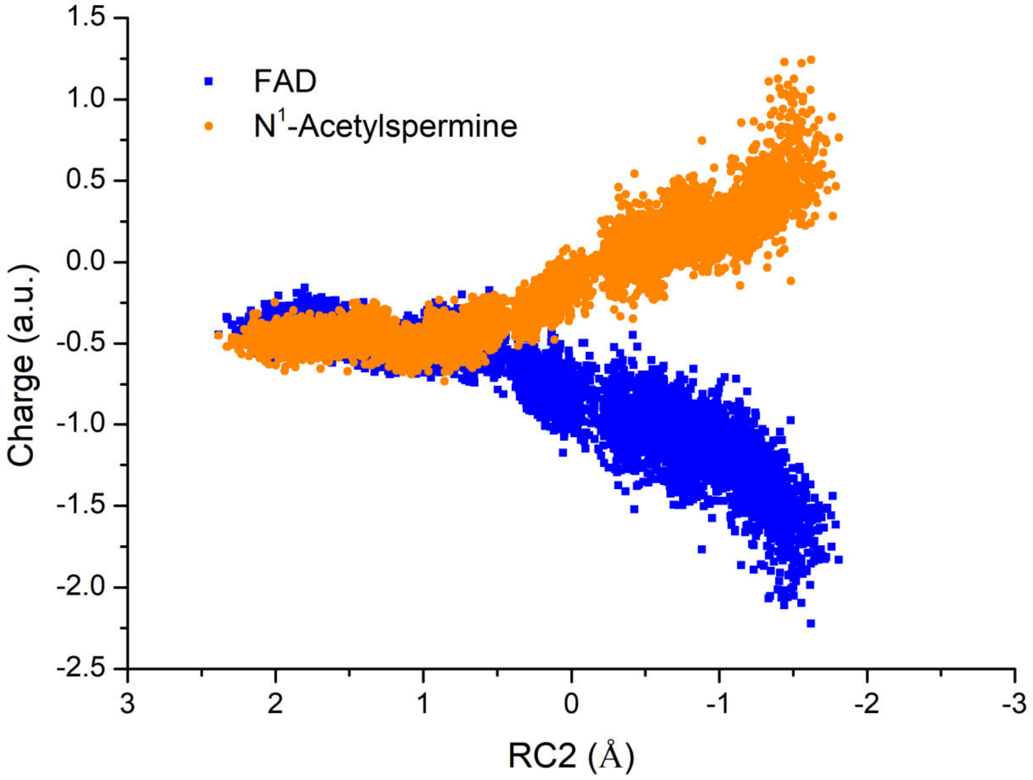

Fig. S19. The changes of charge for FAD and N[1]-Acetylspermine along with the RC2 in the last 5 ps QM/MM MD simulations.

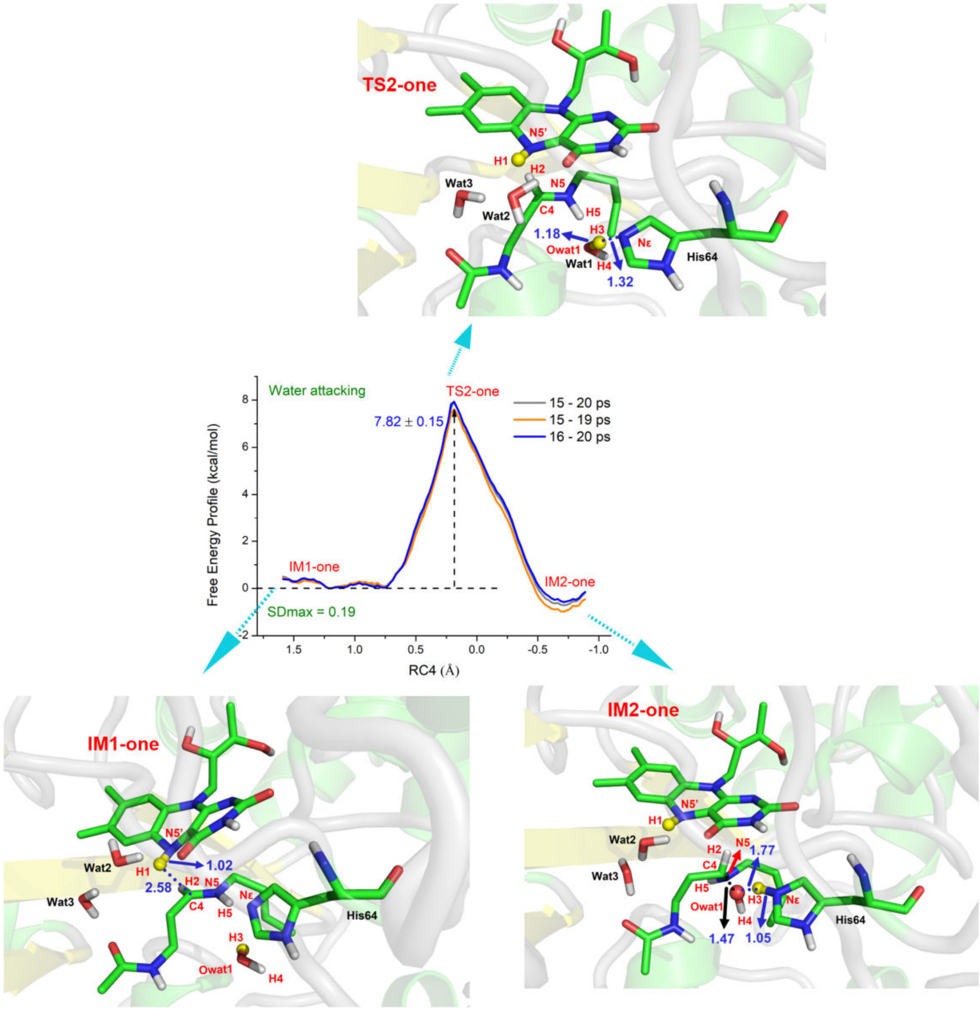

Fig. S20. The structure of reactant, transition state, and intermediate together with corresponding free energy profiles for water attacking to polyamine along RC4 obtained on the basis of last 5 ps QM/MM MD simulations.

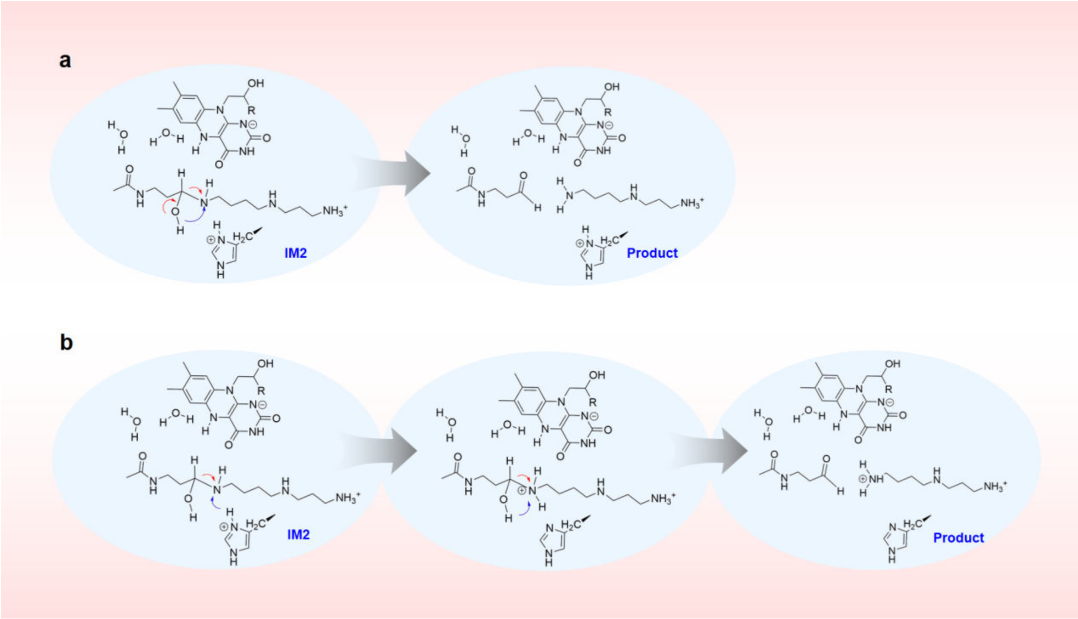

Fig. S21. The prediction of proton transfer and bond cleavage for the generation of final products.

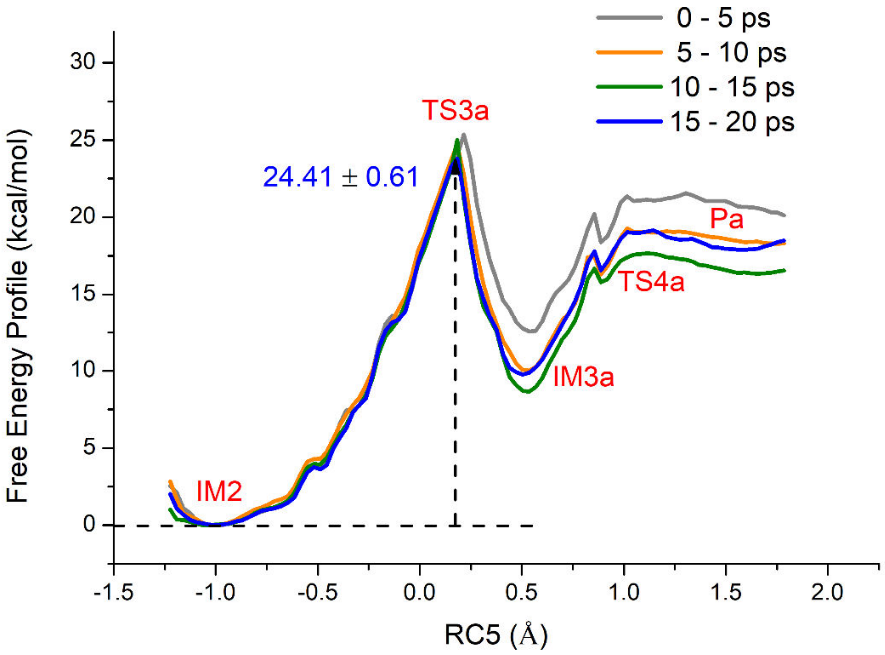

Fig. S22. The free energy profiles of different periods for generation of N-acetyl-3-aminopropannal and spermidine with one terminal amino group protonated obtained by QM/MM MD simulations combined with umbrella sampling.

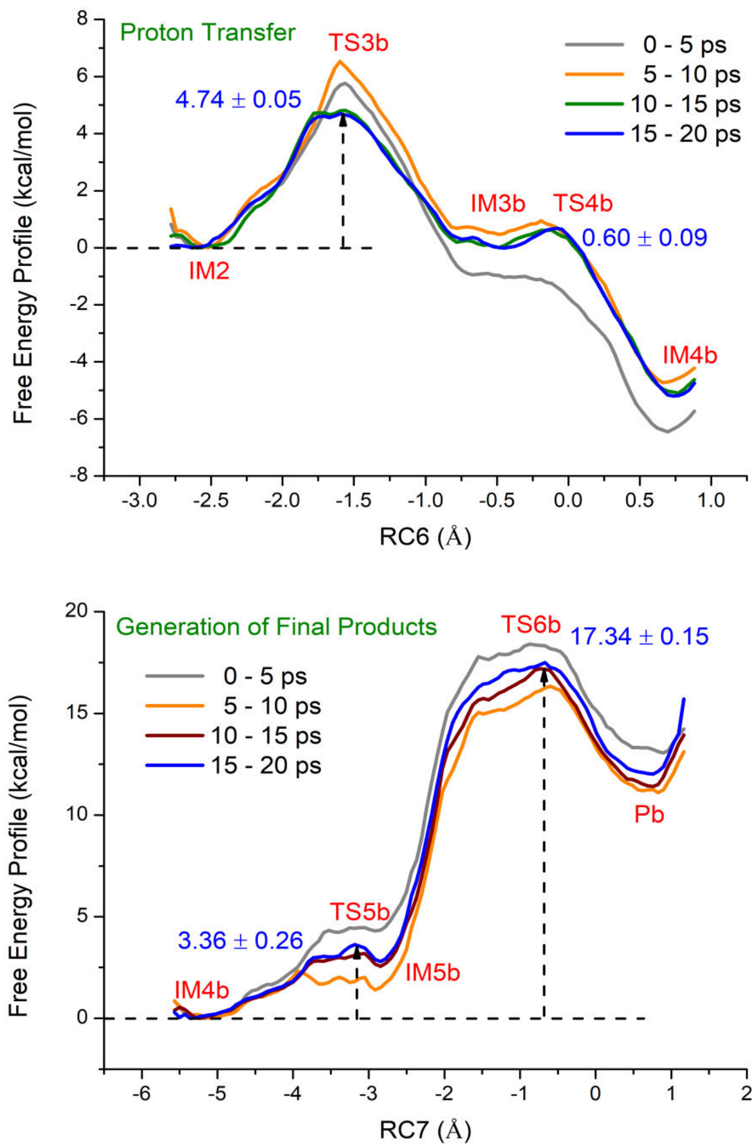

Fig. S23. The free energy profiles of different periods for generation of N-acetyl-3-aminopropannal and spermidine with two terminal amino group protonated obtained by QM/MM MD simulations combined with umbrella sampling.

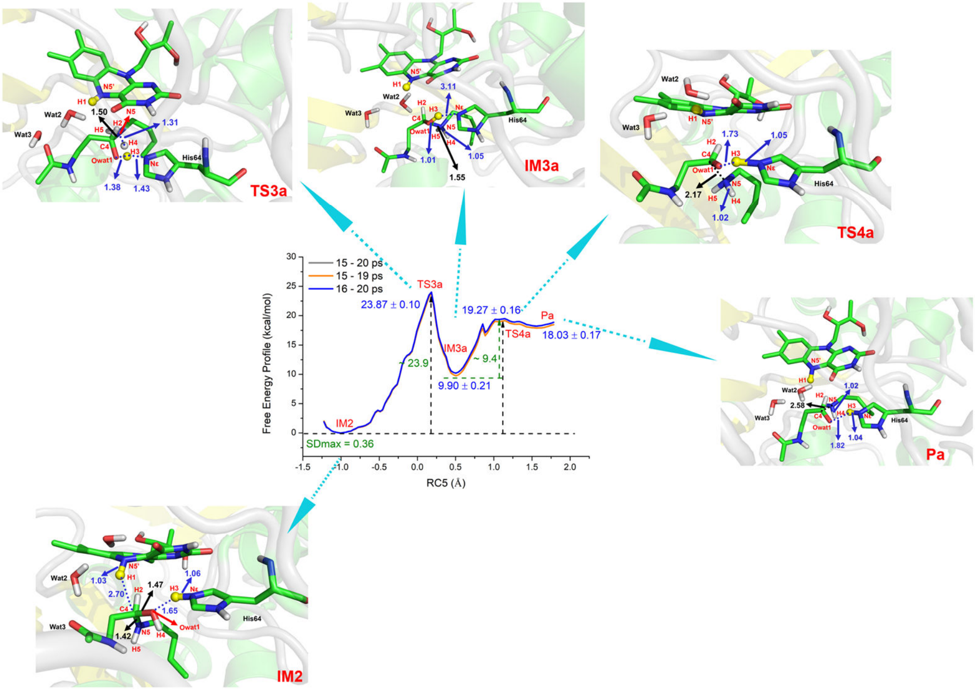

Fig. S24. The structure of reactant, transition states, and intermediates together with corresponding free energy profiles for generation of N-acetyl-3-aminopropannal and spermidine with one terminal amino group protonated along RC5 obtained on the basis of last 5 ps QM/MM MD simulations.

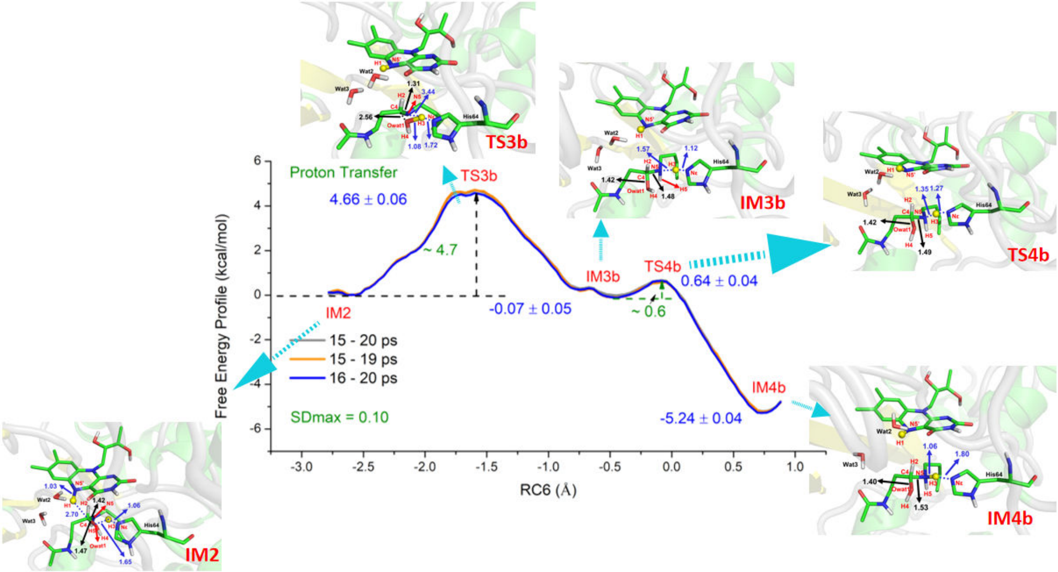

Fig. S25. The structure of reactant, transition states, and intermediates together with corresponding free energy profiles for proton transfer from His64 to polyamine along RC6 obtained on the basis of last 5 ps QM/MM MD simulations.

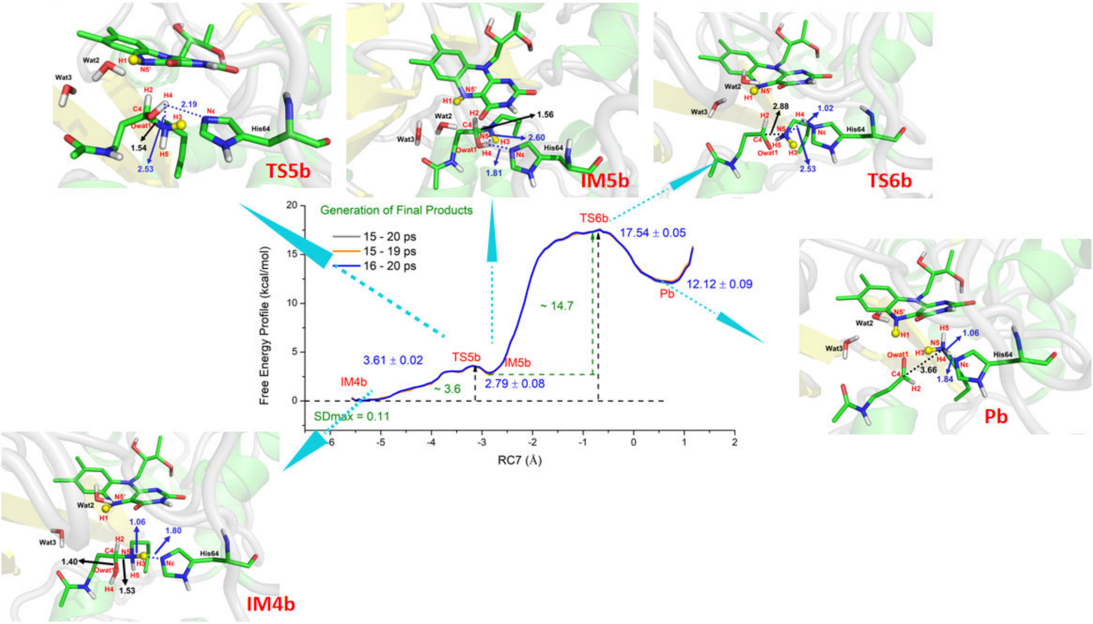

Fig. S26. The structure of reactant, transition states, and intermediates together with corresponding free energy profiles for generation of N-acetyl-3-aminopropannal and spermidine with two terminal amino group protonated along RC7 obtained on the basis of last 5 ps QM/MM MD simulations. The different between Fig. S26 and the Fig. 3g mainly because two free energy profiles along RC6 and RC7 are merged. In the Fig. S26, the IM4b is start point, and the data of different periods is zero, and in Fig. 3g, the IM4b is calculated corresponded to the terminal of RC6 for three sets of data for 15 - 20, 15 - 19, and 16 - 20 ps that are -5.19681, -5.22666, and -5.28190 kcal/mol, respectively.

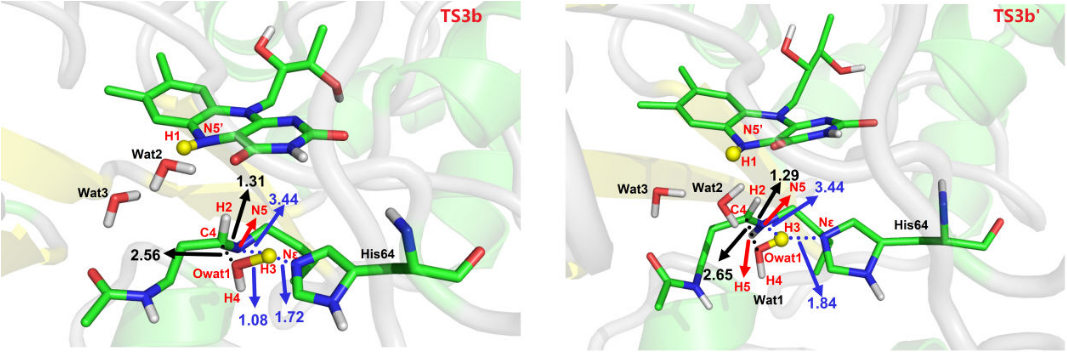

Fig. S27. The structure of TS3b and TS3b' for proton transfer from His64 to polyamine obtained on the basis of last 5 ps QM/MM MD simulations.

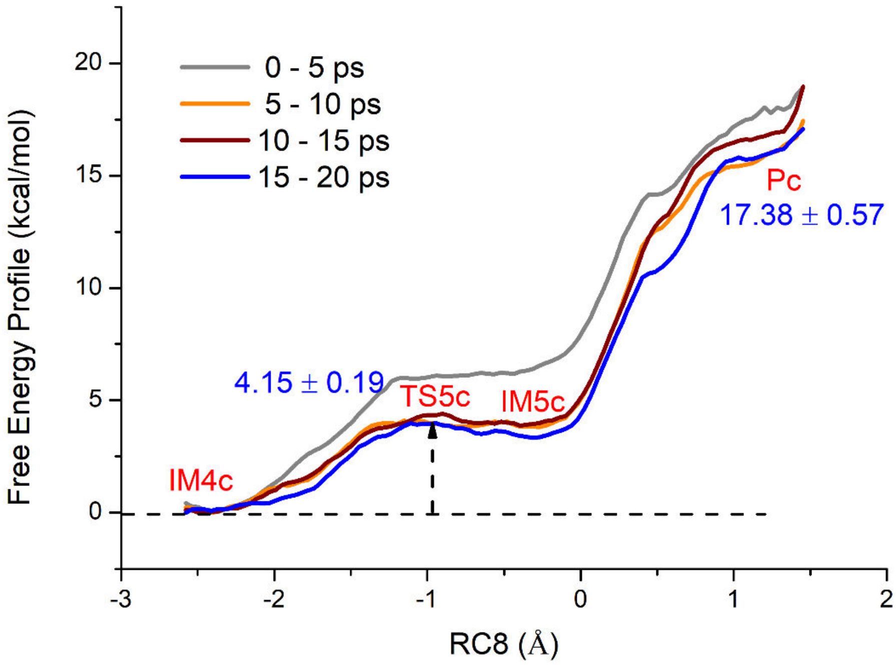

Fig. S28. The free energy profiles of different periods for proton transfer and bond broken along RC8 obtained on the basis of QM/MM MD simulations combined with umbrella sampling.

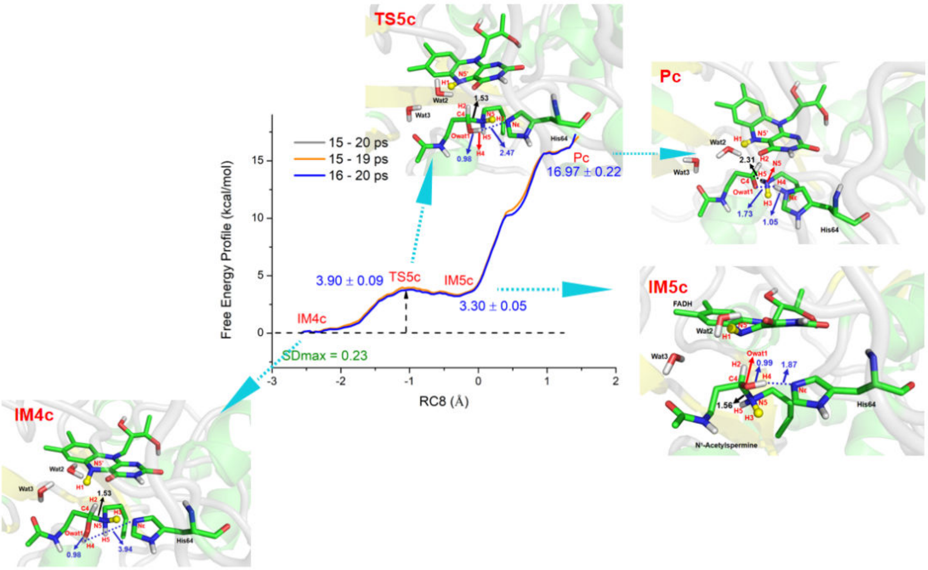

Fig. S29. The structure of reactant, transition state, intermediate, and product together with corresponding free energy profile for proton transfer and bond broken along RC8 obtained on the basis of 5 ps QM/MM MD simulations.

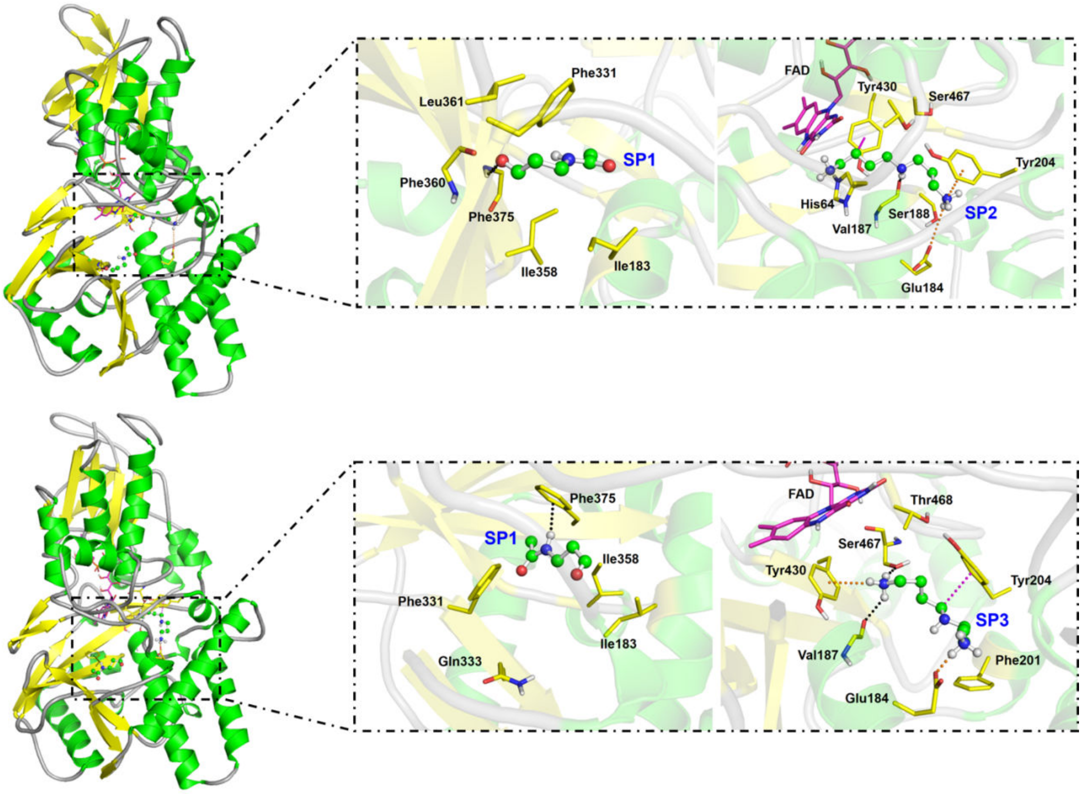

Fig. S30. The static binding mode of products SP1 and SP2 (SP3) in the active site of APAO obtained by MM MD simulations.

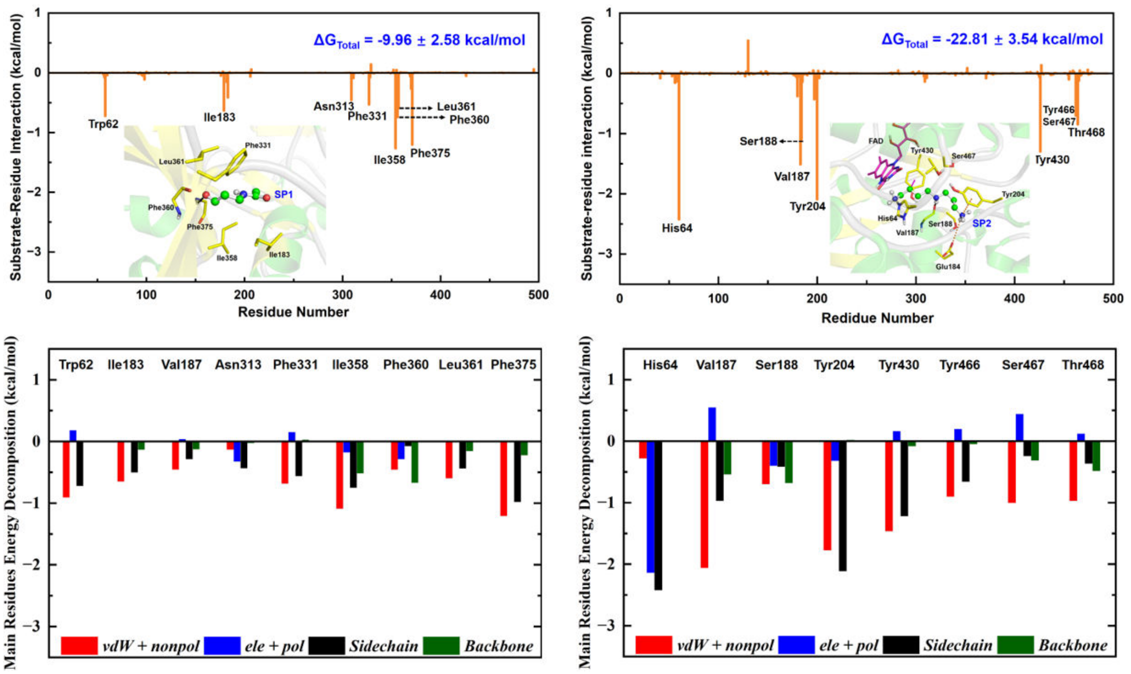

Fig. S31. The contribution for each residue and binding free energy decomposition for key residues for SP1 and SP2 in protein-products complex.

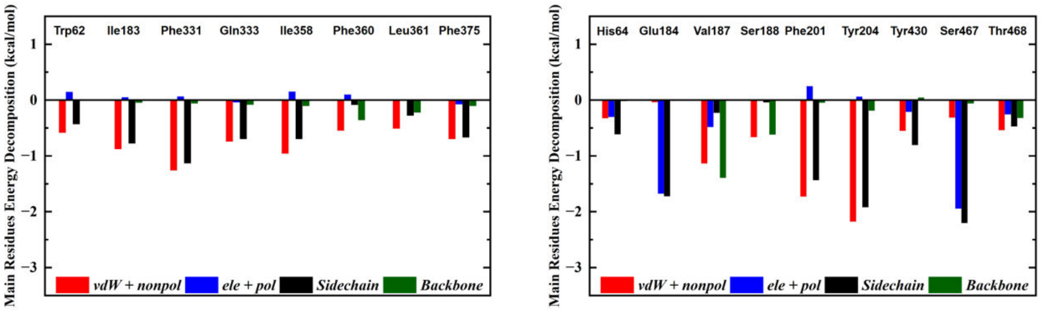

Fig. S32. The binding free energy decomposition for key residues for SP1 and SP3 in protein-products complex.

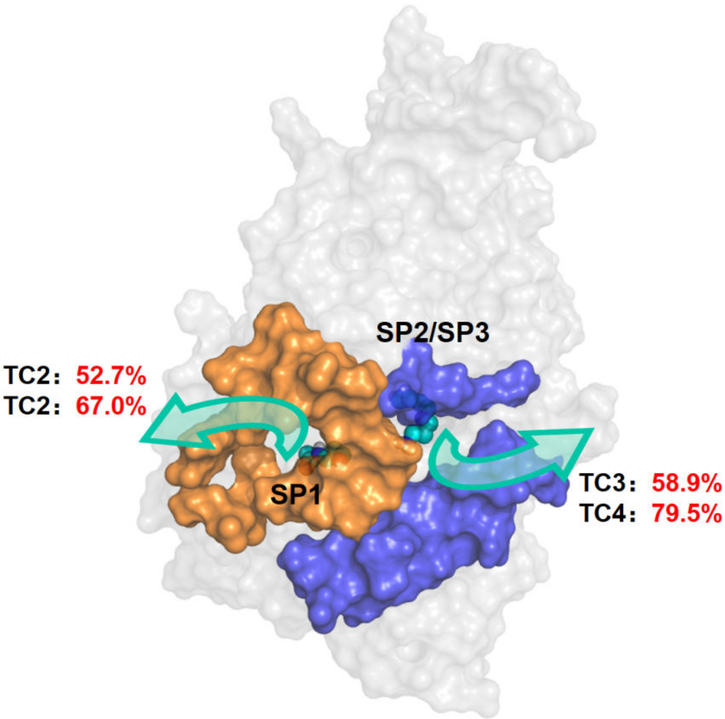

Fig. S33. The possible release channels and corresponding possibilities for two sets of products obtained by RAMD MD simulations.

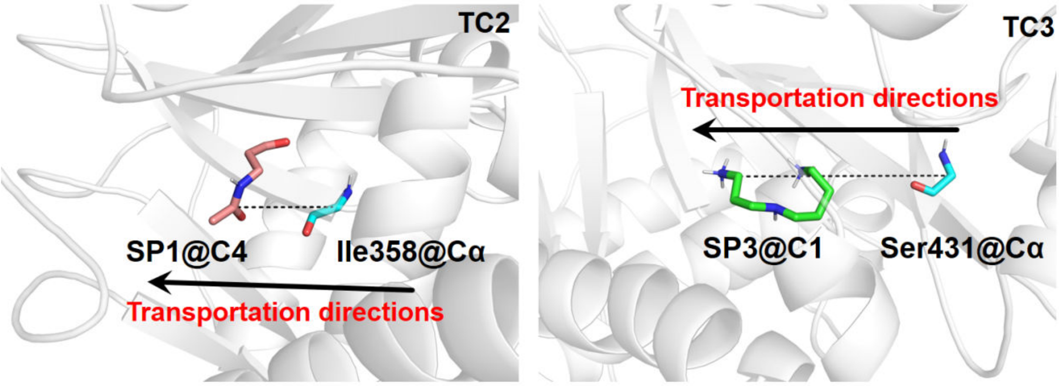

Fig. S34. The transportation coordinates for SP1 and SP3 in QM/MM MD simulations combined with umbrella sampling.

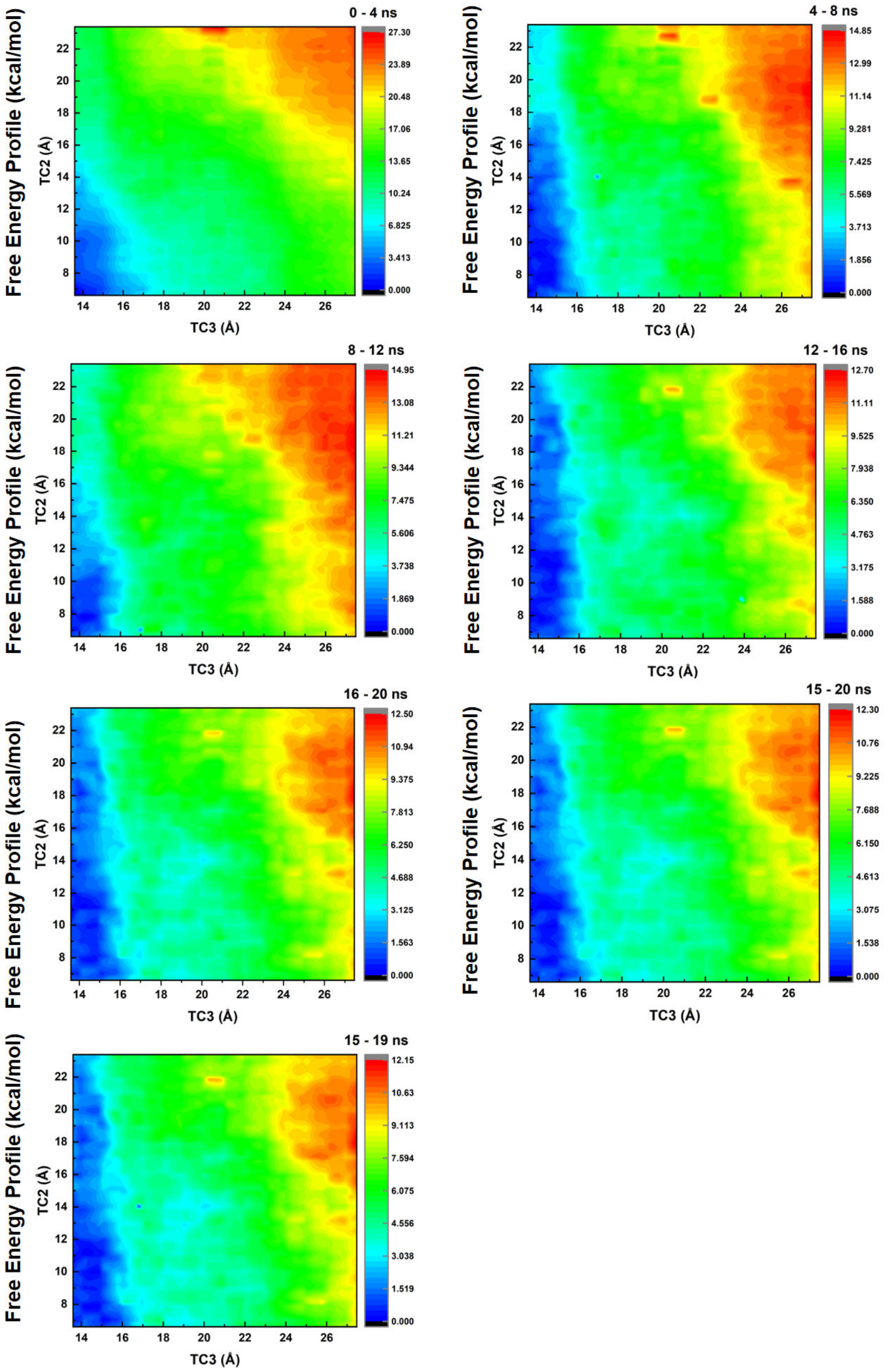

Fig. S35. The 2D free energy profiles obtained by QM/MM MD simulations combined with umbrella sampling for two products release from APAO in different time period.

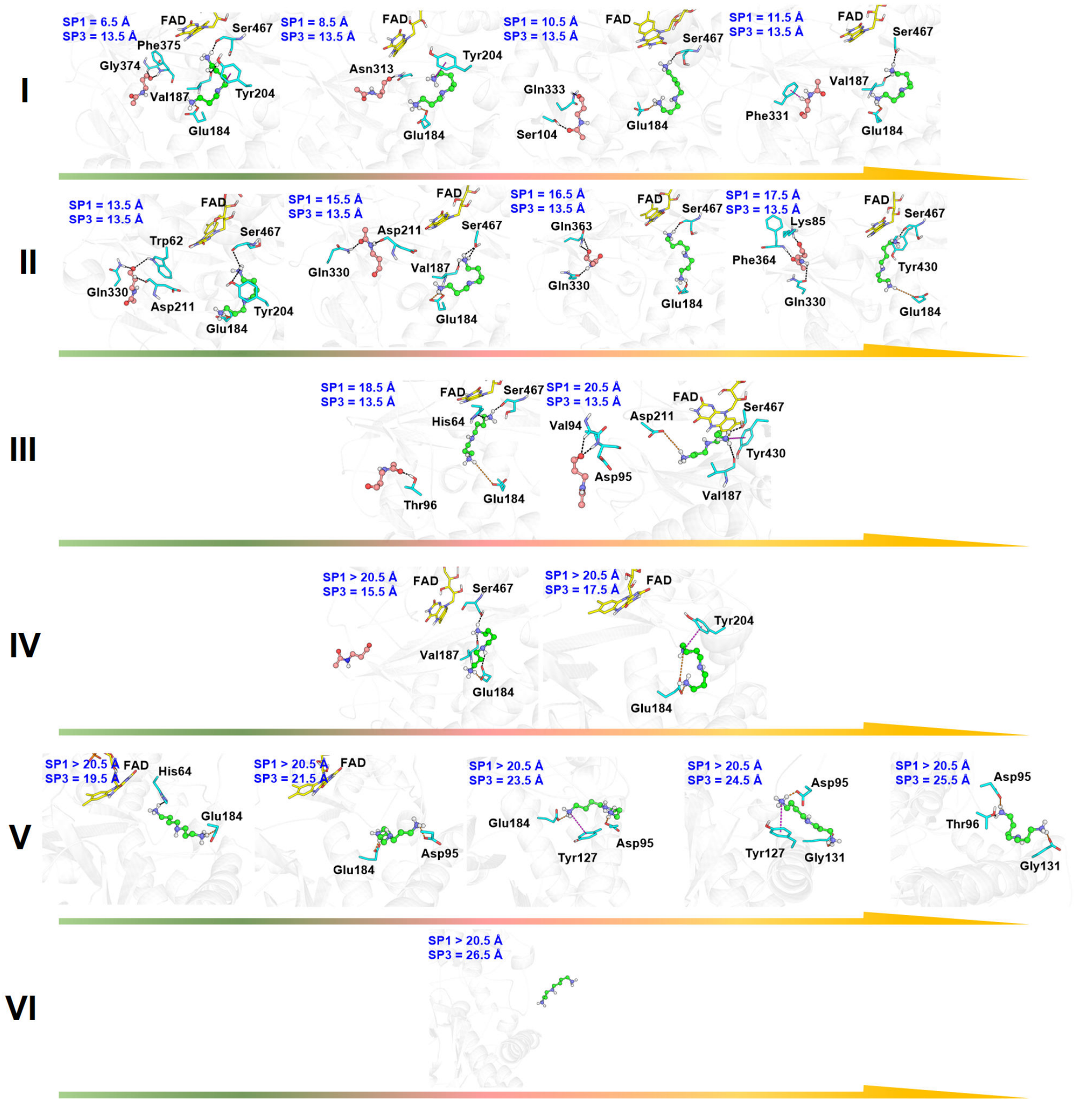

Fig. S36. The represent snapshot of the windows for release of N-acetyl-3-aminopropannal (SP1) and spermidine with two terminal amino group protonated (SP3) obtained by the cluster analysis in the last 5 ns of MM MD simulations.

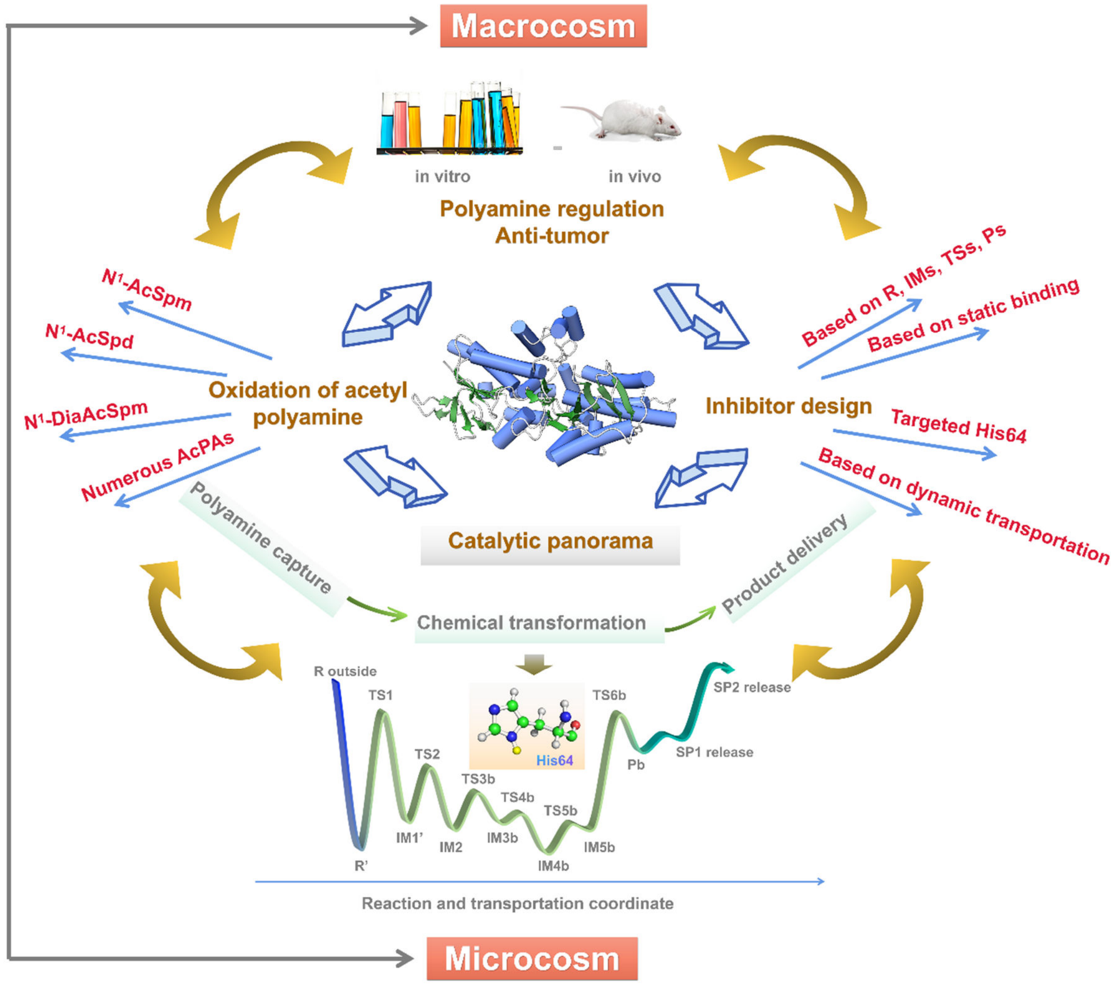

Fig. S37. The bridge of microcosm and macrocosm by catalytic-related network of APAO.

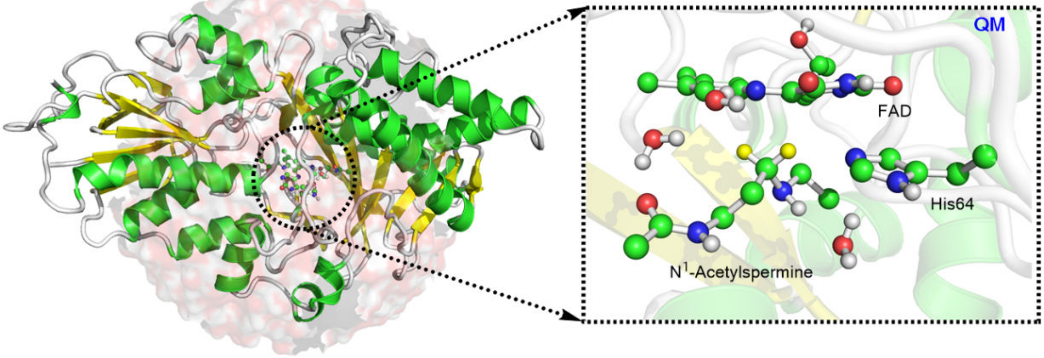

Fig. S38. The QM/MM model building.

Table S1. The location, number, and possibility of trajectories for N^1-acetylspermine delivery pathways based on RAMD MD simulations.[a]

Channel	Location	Number	Probability
P1	Glu84 ~ Pro103; Ser365 ~ Ser367; Pro327 ~ Gln330	85	75.9%
P2	Tyr204 ~ Pro208; Leu116 ~ Phe136; Leu93 ~ Pro103	27	24.1%

[a]The probability is estimated by the ratio of N_x/N_{total}, where N_x is the number of trajectories of channel P_x (x = 1 - 2), and N_{total} is the number of total trajectories.

Table S2. Individual energies terms of MM/GBSA results for the N[1]-acetylspermine-APAO system. (All data units are kcal/mol)

Component	Energy
ΔG_{vdW}	-42.47 ± 2.28
ΔG_{ele}	-388.52 ± 9.70
ΔG_{SA}	-5.97 ± 0.20
ΔG_{GB}	401.81 ± 8.83
ΔG_{gas}	-430.99 ± 9.31
ΔG_{solv}	395.94 ± 8.84
ΔG_{pol}[a]	13.29
ΔG_{nonpol}[b]	-48.44
ΔG_{Total}	-35.05 ± 3.21

[a] $\Delta G_{pol} = \Delta G_{ele} + \Delta G_{GB}$
[b] $\Delta G_{nonpol} = \Delta G_{vdW} + \Delta G_{SA}$

Table S3. The barrier heights with zero-point energy (ZPE) corrections, Wigner correction factor, and active energy barrier calculated by Arrhenius equation with three parameters

Level	ΔE_a (kcal/mol)	Wigner correction factor	$\Delta E_{a\text{-TST}}$ (kcal/mol)	$\Delta E_{a\text{-TST/W}}$ (kcal/mol)
M06-2X/6-31+G(d)	31.65	1.74	31.56	31.00
M06-2X/6-31G(d)	32.75	1.53	32.69	32.16
B3LYP/6-31G(d)	31.92	1.24	31.52	31.15

Table S4. Individual energies terms of MM/GBSA results for the N-acetyl-3-aminopropannal and spermidine with one terminal amino group protonated system. (All data units are kcal/mol)

SP1		SP2	
Component	Energy	Component	Energy
ΔG_{vdW}	-16.89 ± 2.08	ΔG_{vdW}	-25.95 ± 2.37
ΔG_{ele}	-7.19 ± 3.98	ΔG_{ele}	-380.60 ± 14.69
ΔG_{SA}	-2.63 ± 0.25	ΔG_{SA}	-4.01 ± 0.15
ΔG_{GB}	16.74 ± 3.54	ΔG_{GB}	387.75 ± 12.74
ΔG_{gas}	-24.08 ± 4.90	ΔG_{gas}	-406.55 ± 14.50
ΔG_{solv}	14.11 ± 3.44	ΔG_{solv}	383.74 ± 12.71
ΔG_{pol}[a]	9.55	ΔG_{pol}[a]	7.15
ΔG_{nonpol}[b]	-19.52	ΔG_{nonpol}[b]	-29.96
ΔG_{Total}	-9.96 ± 2.58	ΔG_{Total}	-22.81 ± 3.54

[a] $\Delta G_{pol} = \Delta G_{ele} + \Delta G_{GB}$
[b] $\Delta G_{nonpol} = \Delta G_{vdW} + \Delta G_{SA}$

Table S5. Individual energies terms of MM/GBSA results for the N-acetyl-3-aminopropannal and spermidine with two terminal amino group protonated system. (All data units are kcal/mol)

SP1		SP3	
Component	Energy	Component	Energy
ΔG_{vdW}	-15.87 ± 2.49	ΔG_{vdW}	-20.78 ± 3.71
ΔG_{ele}	-7.97 ± 4.40	ΔG_{ele}	-861.42 ± 28.83
ΔG_{SA}	-2.53 ± 0.28	ΔG_{SA}	-3.72 ± 0.20
ΔG_{GB}	17.08 ± 3.86	ΔG_{GB}	861.28 ± 26.10
ΔG_{gas}	-23.85 ± 5.02	ΔG_{gas}	-882.20 ± 26.79
ΔG_{solv}	14.54 ± 3.78	ΔG_{solv}	857.55 ± 26.16
ΔG_{pol}[a]	9.11	ΔG_{pol}[a]	-0.14
ΔG_{nonpol}[b]	-18.40	ΔG_{nonpol}[b]	-24.50
ΔG_{Total}	-9.31 ± 2.79	ΔG_{Total}	-24.65 ± 3.46

[a] $\Delta G_{pol} = \Delta G_{ele} + \Delta G_{GB}$

[b] $\Delta G_{nonpol} = \Delta G_{vdW} + \Delta G_{SA}$

Table S6. The location, number, and possibility of trajectories for N-acetyl-3-aminopropannal (SP1) and spermidine with one terminal amino group protonated (SP2) delivery pathways based on RAMD MD simulations.[a]

	Channel	Location	Number	Probability
SP1	P1	Glu84 ~ Pro103; Ser365 ~ Ser367; Pro327 ~ Gln330	59	52.7%
	P2	Tyr204 ~ Pro208; Leu116 ~ Phe136; Leu93 ~ Pro103	53	47.3%
SP2	P1	Glu84 ~ Pro103; Ser365 ~ Ser367; Pro327 ~ Gln330	46	41.1%
	P2	Tyr204 ~ Pro208; Leu116 ~ Phe136; Leu93 ~ Pro103	66	58.9%

[a]The probability is estimated by the ratio of N_x/N_{total}, where N_x is the number of trajectories of channel P_x (x = 1 - 2), and N_{total} is the number of total trajectories.

Table S7. The location, number, and possibility of trajectories for N-acetyl-3-aminopropannal (SP1) and spermidine with two terminal amino group protonated (SP3) delivery pathways based on RAMD MD simulations.

	Channel	Location	Number	Probability
SP1	P1	Glu84 ~ Pro103; Ser365 ~ Ser367; Pro327 ~ Gln330	75	67.0%
	P2	Tyr204 ~ Pro208; Leu116 ~ Phe136; Leu93 ~ Pro103	37	33.0%
SP3	P1	Glu84 ~ Pro103; Ser365 ~ Ser367; Pro327 ~ Gln330	23	20.5%
	P2	Tyr204 ~ Pro208; Leu116 ~ Phe136; Leu93 ~ Pro103	89	79.5%

Appendix

The Cartesian coordinates for reactant and transition state obtained at M06-2X/6-31+G(d), M06-2X/6-31G(d), and B3LYP/6-31G(d) levels.

M06-2X/6-31+G(d), reactant

O	-4.12640900	-0.76857700	1.64202700
C	-2.98694900	-0.65015900	2.04888300
N	-2.40991800	-1.52395200	2.92934500
H	-2.97278600	-2.30985800	3.24133200
C	-1.08132000	-1.49672700	3.41775700
O	-0.71541500	-2.40233900	4.13509600
N	-0.27565800	-0.43606700	3.06157300
C	-0.72509400	0.45385700	2.22571900
C	-2.06776300	0.45674700	1.63516400
N	-2.50078200	1.32304800	0.78752200
C	-1.64111900	2.31952000	0.38801900
C	-2.08588900	3.25117800	-0.56501200
H	-3.10338000	3.13702500	-0.93047400
C	-1.27022300	4.26905400	-1.02102600
C	-1.76484000	5.25938800	-2.04234700
H	-2.79677400	5.04074100	-2.32676900
H	-1.15162200	5.23886200	-2.95042600
H	-1.73165800	6.28369600	-1.65379100
C	0.04799700	4.36340800	-0.50406400
C	0.96398700	5.45820600	-0.98198900
H	0.53886600	6.44653700	-0.77293400
H	1.11698900	5.39839100	-2.06558300
H	1.94134300	5.39858100	-0.49755500
C	0.49966200	3.45065700	0.44057500
H	1.51231000	3.54684400	0.81609800
C	-0.33303300	2.41870800	0.90188300
N	0.08873000	1.48667600	1.83888900
C	1.45102500	1.56238400	2.36340400
H	1.57861500	0.76075800	3.08638600
H	2.16693200	1.44217500	1.54364800
C	0.82134200	-0.91266300	-0.16823800
H	1.26008500	-0.94733900	0.83956700
N	-0.53470600	-1.44152400	-0.06960200
H	-0.50014600	-2.43506900	0.15948200
C	-1.37070300	-1.22926500	-1.25043000
H	-1.51946000	-0.14186400	-1.34254800
H	-0.88506700	-1.55711600	-2.18362900
C	-2.72709600	-1.92791800	-1.08377100
H	-2.84159200	-2.24594300	-0.04102900
H	1.60222900	2.53074200	2.84704300
H	-2.77699700	-2.83820100	-1.69399900
H	0.74069400	0.14998800	-0.44530000
C	-3.90791300	-1.01112900	-1.42042000
H	-3.89077100	-0.73497500	-2.48076400
H	-3.81646300	-0.08716700	-0.84036500

N	-5.19029300	-1.60111200	-1.09484700
H	-5.47428300	-1.55164400	-0.12058100
C	-5.97094300	-2.37808500	-1.89560000
O	-6.98545000	-2.91864000	-1.47824400
C	-5.54227900	-2.51205800	-3.34730900
H	-5.63044700	-1.55041800	-3.86420200
H	-4.50573500	-2.85115000	-3.44010400
H	-6.20481600	-3.23389500	-3.82418700
C	1.76649200	-1.61498600	-1.14670900
H	1.38469700	-1.52710600	-2.17240900
H	1.79365700	-2.68810800	-0.90845300
C	3.17822900	-1.03569600	-1.07370400
H	3.55641700	-1.15027700	-0.04886100
H	3.15973000	0.04171400	-1.28735200
C	4.13498300	-1.70195100	-2.05876200
H	4.15639100	-2.79097500	-1.85959800
H	3.74267200	-1.56735100	-3.07379400
N	5.46915200	-1.11342100	-2.01400100
H	5.97842100	-1.37567700	-2.85531000
C	6.25163000	-1.51499600	-0.85025600
H	6.23820400	-2.61300000	-0.70599200
H	5.79626100	-1.07439200	0.04522500
C	7.68995300	-1.02702400	-0.96513200
H	8.15086700	-1.46360800	-1.86487000
H	7.70245800	0.06034100	-1.09611000
C	8.52962600	-1.39592600	0.25228700
H	8.48507200	-2.48694800	0.40981700
H	8.09643600	-0.92661900	1.14365700
N	9.89066300	-0.88250300	0.09973200
H	10.44216900	-1.06712400	0.93359900
H	10.35977700	-1.34927300	-0.67368300

M06-2X/6-31+G(d), transition state, 898.70i

O	-3.32705800	-0.97861000	2.19530800
C	-2.29380300	-0.43660600	2.59265400
N	-1.62521300	-0.86637700	3.71838600
H	-2.06228500	-1.59172800	4.27717600
C	-0.38100900	-0.38636800	4.18451500
O	0.08382600	-0.86881300	5.20091900
N	0.24182500	0.58659000	3.44104500
C	-0.34170800	1.04655200	2.35496400
C	-1.61260800	0.61577600	1.87075500
N	-2.21420500	1.06936200	0.73035100
C	-1.53089000	2.10622400	0.05424000
C	-2.12334200	2.67121400	-1.07580200
H	-3.10075200	2.29498800	-1.37306400
C	-1.51137200	3.68636000	-1.80098500
C	-2.17762600	4.27767700	-3.01585300
H	-3.13971400	3.79578400	-3.20811900
H	-1.55625900	4.16195200	-3.91174800
H	-2.35959900	5.35119300	-2.88843500
C	-0.25918400	4.16174800	-1.36201300
C	0.43612300	5.27122800	-2.10761000

H	-0.16988700	6.18466200	-2.11440600
H	0.61539700	4.99786400	-3.15389900
H	1.39997200	5.50988400	-1.65115300
C	0.33247800	3.60789600	-0.22986700
H	1.29596800	3.99015200	0.08875900
C	-0.28225800	2.57533800	0.49294800
N	0.31957500	2.01113900	1.62179500
C	1.61981400	2.49751600	2.06257400
H	1.90790800	1.92925200	2.94332300
H	2.36057600	2.35654100	1.26822900
C	0.63912500	-0.99567000	0.24991400
H	1.06934100	-0.89123500	1.25213200
N	-0.75517600	-1.40177400	0.39281300
H	-0.97086400	-2.09665700	1.10208200
C	-1.69720500	-1.13409100	-0.53234700
H	-2.30447900	0.16093300	0.02007700
H	-1.31352400	-0.67335500	-1.44714100
C	-2.92099700	-2.01292300	-0.62147900
H	-3.03210800	-2.56589400	0.31658200
H	1.56081300	3.56090100	2.31735600
H	-2.78133900	-2.74457200	-1.43186000
H	0.64329000	0.00139500	-0.20854800
C	-4.21755300	-1.22375600	-0.85481600
H	-4.15527600	-0.64427400	-1.78323000
H	-4.34905200	-0.51953400	-0.02843800
N	-5.38158200	-2.08312900	-0.90304900
H	-5.79725900	-2.35816800	-0.01971700
C	-5.89340200	-2.72976500	-1.99248600
O	-6.80867400	-3.52997100	-1.88522500
C	-5.29724800	-2.37280000	-3.34214500
H	-5.52983200	-1.33408400	-3.60002200
H	-4.20900200	-2.48909500	-3.35689200
H	-5.74149800	-3.03270300	-4.08670600
C	1.46048400	-1.96851700	-0.59490100
H	0.96717900	-2.10575800	-1.56699500
H	1.47774800	-2.95107100	-0.10416300
C	2.88432300	-1.45555900	-0.80215000
H	3.34953000	-1.27901000	0.17640800
H	2.86516400	-0.49159400	-1.32746600
C	3.73156900	-2.42827400	-1.62009900
H	3.85122000	-3.37010000	-1.05034500
H	3.19202600	-2.67356800	-2.54284600
N	5.01233600	-1.84652500	-2.00000000
H	5.43043600	-2.40924300	-2.73783900
C	5.96308000	-1.73060300	-0.89959300
H	6.06248200	-2.68126600	-0.34012600
H	5.58339300	-0.98486700	-0.19016300
C	7.32705800	-1.28167800	-1.40750000
H	7.70923000	-2.02210500	-2.12727700
H	7.22536800	-0.33444900	-1.94785600
C	8.34202200	-1.11128600	-0.28340100
H	8.41632100	-2.05199400	0.28822500
H	7.98302400	-0.34274000	0.41131000

N	9.62074100	-0.65410500	-0.82622500
H	10.28450600	-0.46672700	-0.07942000
H	10.03373400	-1.37464000	-1.41471900

M06-2X/6-31G(d), reactant

O	-4.09802700	-0.76770800	1.60899900
C	-2.96223700	-0.64128100	2.01920300
N	-2.38004600	-1.50737500	2.90393900
H	-2.93639900	-2.29836500	3.21052500
C	-1.05353500	-1.47320800	3.39906800
O	-0.68345800	-2.37243900	4.11647800
N	-0.25533700	-0.40393500	3.04215100
C	-0.71001600	0.47995400	2.20669300
C	-2.04993300	0.47186000	1.60881700
N	-2.48751800	1.33385200	0.76017500
C	-1.63408400	2.33846100	0.36478500
C	-2.08324900	3.26804400	-0.58623000
H	-3.09754800	3.14488900	-0.95547300
C	-1.27572200	4.29367200	-1.03615200
C	-1.77451700	5.28156600	-2.05695200
H	-2.80353900	5.05619300	-2.34493800
H	-1.15818100	5.26741000	-2.96273500
H	-1.74925700	6.30533200	-1.66717400
C	0.03871900	4.39833200	-0.51522700
C	0.94535600	5.50255500	-0.98798300
H	0.51059700	6.48609200	-0.77770800
H	1.10058000	5.44746800	-2.07121200
H	1.92193600	5.45101800	-0.50199900
C	0.49565100	3.48774900	0.42684900
H	1.50579800	3.59111800	0.80579400
C	-0.32900900	2.44857000	0.88289400
N	0.09830900	1.51731400	1.81729500
C	1.46016400	1.59658700	2.33962100
H	1.58716500	0.79410100	3.06147900
H	2.17479800	1.47895800	1.51873400
C	0.81107100	-0.90908000	-0.17309500
H	1.24938800	-0.93602200	0.83502400
N	-0.54574600	-1.43471400	-0.07137600
H	-0.50642100	-2.42979000	0.14812600
C	-1.37538700	-1.22764500	-1.25731900
H	-1.52935400	-0.14159000	-1.35039400
H	-0.88414700	-1.55179000	-2.18867600
C	-2.72883400	-1.93330900	-1.09490600
H	-2.83834400	-2.25730900	-0.05416700
H	1.61068600	2.56512900	2.82246100
H	-2.77593700	-2.84004900	-1.70992400
H	0.73288900	0.15101000	-0.45906500
C	-3.91821700	-1.02289900	-1.41967000
H	-3.92380300	-0.76235700	-2.48376000
H	-3.81459600	-0.09128100	-0.85458000
N	-5.19017300	-1.60996300	-1.05803800
H	-5.43326800	-1.56867500	-0.07371900
C	-5.96683600	-2.43685200	-1.81474300

O	-6.94178800	-3.00513300	-1.35388000
C	-5.57755700	-2.57886900	-3.27778900
H	-5.67890400	-1.62170500	-3.79937700
H	-4.54323300	-2.91666500	-3.39514400
H	-6.25109300	-3.30611100	-3.72925600
C	1.75352700	-1.62374900	-1.14418400
H	1.36941200	-1.55020800	-2.16977900
H	1.78062500	-2.69347600	-0.89271400
C	3.16614800	-1.04675000	-1.08292900
H	3.54209500	-1.13587800	-0.05497400
H	3.15208100	0.02439400	-1.32230000
C	4.12356200	-1.73968500	-2.04827100
H	4.13507100	-2.82421900	-1.82629600
H	3.73401300	-1.62603500	-3.06677900
N	5.45797100	-1.15274500	-2.01041000
H	5.96563800	-1.44113000	-2.84427600
C	6.23662400	-1.55500000	-0.84572500
H	6.23531900	-2.65309000	-0.70110700
H	5.77273700	-1.12410600	0.05019600
C	7.66937500	-1.04973800	-0.95031800
H	8.13869900	-1.47625800	-1.85014600
H	7.66956200	0.03726600	-1.07782900
C	8.50936400	-1.41333900	0.26775000
H	8.46972000	-2.50568400	0.42343000
H	8.06503900	-0.95313700	1.15840100
N	9.86328500	-0.88229600	0.12040800
H	10.40907600	-1.09015400	0.95311400
H	10.32949500	-1.35969900	-0.64869800

M06-2X/6-31G(d), transition state, 761.75i

O	-3.32323900	-1.00609800	2.14285000
C	-2.29596300	-0.46683600	2.55416100
N	-1.61814000	-0.92382000	3.66417700
H	-2.05010900	-1.66411500	4.20448600
C	-0.37666500	-0.44912100	4.14661000
O	0.09117800	-0.95432100	5.14597500
N	0.23772600	0.54890000	3.42529700
C	-0.35312700	1.03226300	2.35563800
C	-1.62038100	0.60657400	1.86186400
N	-2.22175900	1.07638600	0.72558700
C	-1.53687900	2.12061900	0.06220100
C	-2.12005500	2.69085700	-1.06813300
H	-3.08890600	2.30802900	-1.38232100
C	-1.50912400	3.71829500	-1.77466500
C	-2.16438900	4.31469800	-2.99224900
H	-3.11922000	3.82674400	-3.20203600
H	-1.53027800	4.21385900	-3.88067300
H	-2.35693400	5.38516400	-2.85760700
C	-0.26880500	4.20125500	-1.31575800
C	0.42357200	5.32438300	-2.04238400
H	-0.19164100	6.23129300	-2.04835200
H	0.61716900	5.06343600	-3.08900700
H	1.37919300	5.56885400	-1.57292700

C	0.31476100	3.64129000	-0.18400600
H	1.27147300	4.02721400	0.14891200
C	-0.29759400	2.59621900	0.52009500
N	0.29991400	2.02149000	1.64537500
C	1.58902400	2.51412500	2.10628400
H	1.86573500	1.93990400	2.98662600
H	2.34257200	2.38552600	1.32202600
C	0.64201100	-0.97083600	0.23482500
H	1.06904500	-0.86085400	1.23725800
N	-0.75199700	-1.37844000	0.37858700
H	-0.96562400	-2.07534300	1.08566500
C	-1.68914500	-1.12507300	-0.55182000
H	-2.32577900	0.19002000	0.01606300
H	-1.30824600	-0.65076300	-1.45939600
C	-2.90920500	-2.00600600	-0.64654800
H	-3.01140000	-2.56935200	0.28566900
H	1.51919100	3.57547400	2.36538100
H	-2.77381100	-2.72655800	-1.46703900
H	0.64351500	0.02331400	-0.22894200
C	-4.21302200	-1.22141500	-0.85918500
H	-4.16349700	-0.63556700	-1.78406700
H	-4.33242400	-0.52520500	-0.02486800
N	-5.37232500	-2.08415400	-0.89816600
H	-5.76339200	-2.37902400	-0.01133200
C	-5.87682500	-2.75347600	-1.97992100
O	-6.76383400	-3.57775800	-1.86084300
C	-5.30181200	-2.37814200	-3.33477500
H	-5.53870100	-1.33748500	-3.57827900
H	-4.21381800	-2.49152700	-3.36656500
H	-5.75546200	-3.03238800	-4.07790700
C	1.46254200	-1.94855400	-0.60429100
H	0.96899000	-2.08959300	-1.57522300
H	1.47787500	-2.92880700	-0.11005500
C	2.88715000	-1.44010700	-0.81363700
H	3.35282900	-1.25972700	0.16357400
H	2.87156900	-0.47916700	-1.34272800
C	3.73287000	-2.41704700	-1.62758700
H	3.84252100	-3.35956000	-1.05734400
H	3.19298800	-2.66060600	-2.55047800
N	5.01413000	-1.83684900	-2.00495800
H	5.42998800	-2.40842300	-2.73737000
C	5.96457400	-1.74332600	-0.90346800
H	6.06885500	-2.70154800	-0.35790900
H	5.58560800	-1.01214400	-0.17867800
C	7.32574800	-1.28029600	-1.40511500
H	7.70990600	-2.00798800	-2.13619300
H	7.21854900	-0.32724700	-1.93230300
C	8.34224900	-1.12058200	-0.28157900
H	8.41480700	-2.06793600	0.28090300
H	7.97830500	-0.36387100	0.42341700
N	9.61616400	-0.65020500	-0.82207800
H	10.27648500	-0.49213500	-0.06494600
H	10.02418600	-1.37819400	-1.40537000

B3LYP/6-31G(d), reactant

O	4.39738900	0.67958000	-0.62771500
C	4.39687500	-0.41997000	-0.09471100
N	5.45353800	-0.89970400	0.64038900
H	6.26196500	-0.29277600	0.73284200
C	5.55336700	-2.15311100	1.30369300
O	6.57374400	-2.41395500	1.91013800
N	4.47100800	-3.00846800	1.21879100
C	3.41382700	-2.65082900	0.54107800
C	3.25395400	-1.38380100	-0.17126800
N	2.19986700	-1.04558800	-0.85278700
C	1.15604700	-1.92900800	-0.91132800
C	0.00886400	-1.57723400	-1.65185800
H	0.02476100	-0.61373300	-2.15230100
C	-1.08986100	-2.41324500	-1.74714900
C	-2.30322700	-2.00270200	-2.54518200
H	-2.14845400	-1.02860900	-3.01721400
H	-3.19847600	-1.92861400	-1.91465700
H	-2.53191700	-2.72601600	-3.33783300
C	-1.04597600	-3.66705900	-1.07171700
C	-2.22108600	-4.60924200	-1.14340800
H	-2.43465400	-4.90247300	-2.17902100
H	-3.13349500	-4.13890100	-0.75625100
H	-2.03977700	-5.52001400	-0.56630600
C	0.08149800	-4.03291300	-0.34099500
H	0.08483900	-4.99333800	0.16043800
C	1.19630500	-3.18307900	-0.24686700
N	2.33592300	-3.51765000	0.46909700
C	2.41445100	-4.80761600	1.16538200
H	3.38749200	-4.86223000	1.64762500
H	1.62067000	-4.87294900	1.91530700
C	-1.57408300	1.49280300	1.15266200
H	-1.92418900	1.17228200	2.14383500
N	-0.28590500	2.15761600	1.33579400
H	-0.40431900	2.98509100	1.91899300
C	1.84458900	2.98408100	0.40232700
H	2.37159800	2.18309700	0.93144900
H	2.30668700	-5.62442400	0.44575900
H	1.82787700	3.85278600	1.07685700
H	-1.38768100	0.57249400	0.58054800
C	2.62271800	3.33917600	-0.87510800
H	2.15909200	4.19320700	-1.38110000
H	2.57742900	2.49049600	-1.56579300
N	4.02992100	3.62298700	-0.64117900
H	4.62948400	2.81119000	-0.53555100
C	4.62298700	4.82013500	-0.35606700
O	5.81167900	4.89266400	-0.05931500
C	3.74927300	6.06684600	-0.45632700
H	3.38779400	6.21751100	-1.48070200
H	2.87263500	6.00918500	0.19896700
H	4.35800300	6.92407500	-0.16616200
C	-2.69552600	2.29451900	0.46379100

H	-2.35936000	2.62599700	-0.52836200
H	-2.89261800	3.20873100	1.04425000
C	-3.98871700	1.48295200	0.31653500
H	-4.32161500	1.15017600	1.30956600
H	-3.79613800	0.57144000	-0.26565000
C	-5.11266000	2.26116500	-0.37733600
H	-5.34107600	3.17257000	0.21072400
H	-4.74914500	2.60235100	-1.35516900
N	-6.30227200	1.43852900	-0.61560700
H	-6.86206200	1.88207000	-1.34218700
C	-7.15295400	1.24959400	0.56009100
H	-7.43315500	2.21151800	1.03643800
H	-6.58260000	0.69081100	1.31297900
C	-8.41410100	0.46145300	0.20292700
H	-8.97816700	1.01167800	-0.56774000
H	-8.13357300	-0.49939200	-0.24225900
C	-9.32800400	0.21177200	1.40438300
H	-9.58583700	1.17974400	1.87395800
H	-8.77895100	-0.36352500	2.16134800
N	-10.49460300	-0.58521000	1.00495600
H	-11.07557500	-0.77562200	1.82010400
H	-11.07677000	-0.03812800	0.37099700
C	0.41614900	2.52220700	0.10571200
H	-0.10479100	3.29482900	-0.49260100
H	0.46100300	1.62214400	-0.52273700

B3LYP/6-31G(d), transition state, 510.97i

O	2.53446900	1.12253900	2.71161800
C	1.68300300	0.22781900	2.80500600
N	0.77343600	0.18570400	3.84780500
H	0.87129100	0.88276100	4.57682100
C	-0.30604400	-0.72170400	3.99401800
O	-1.04800300	-0.60442000	4.95744300
N	-0.45267400	-1.68124700	3.01708700
C	0.39030400	-1.71871500	2.00347200
C	1.47354000	-0.80888900	1.81405600
N	2.33996300	-0.84809600	0.74675900
C	2.16843900	-1.91423900	-0.16215200
C	3.06611900	-2.04984100	-1.22555500
H	3.87142000	-1.32306600	-1.30323100
C	2.96599900	-3.07810900	-2.15934200
C	3.95863100	-3.18260300	-3.29204000
H	4.69178800	-2.37148300	-3.24980900
H	3.46621500	-3.13706200	-4.27227400
H	4.51122800	-4.13055300	-3.26199000
C	1.92525800	-4.02228600	-2.00740400
C	1.77108100	-5.16450400	-2.98284900
H	2.66229000	-5.80512000	-2.99908800
H	1.62501900	-4.80398100	-4.00934600
H	0.91414000	-5.79416300	-2.72592600
C	1.03388000	-3.89863000	-0.94054300
H	0.24326600	-4.63354500	-0.84458700
C	1.12755300	-2.85298100	-0.00719200

N	0.22485800	-2.72542900	1.05625700
C	-0.83422900	-3.71542500	1.23654000
H	-1.40466800	-3.42709100	2.11674500
H	-1.48873200	-3.73875400	0.35820300
C	-0.95580600	0.74522200	0.04891600
H	-1.41439000	0.26734100	0.92091700
N	0.32864400	1.30798000	0.46650000
H	0.32590200	1.86524200	1.31383500
C	1.42673200	1.40589100	-0.32214000
H	2.30488800	0.14002700	0.21922200
H	1.28613400	1.02633600	-1.33523400
C	2.39996900	2.54417100	-0.10968000
H	2.24330400	2.96644900	0.88759400
H	-0.40534000	-4.71263500	1.38811200
H	2.18779400	3.33904700	-0.84309000
H	-0.74994800	-0.04489400	-0.68076900
C	3.88604900	2.13961300	-0.22166900
H	4.08151800	1.65943900	-1.18630000
H	4.10295600	1.41008500	0.56311500
N	4.79042000	3.26437100	-0.05964500
H	4.99215500	3.56781800	0.88598100
C	5.29938600	4.10130600	-1.02219600
O	5.96536100	5.08227800	-0.71897700
C	5.02492400	3.73109700	-2.47477300
H	5.48253800	2.76839800	-2.73199100
H	3.95226500	3.65782500	-2.68783900
H	5.46096600	4.50853200	-3.10288600
C	-1.90324200	1.79598500	-0.54989900
H	-1.42246900	2.25482000	-1.42519500
H	-2.05227400	2.60177900	0.18257000
C	-3.25799800	1.19494700	-0.94422300
H	-3.71959400	0.73630100	-0.05980800
H	-3.12051900	0.38940900	-1.67699700
C	-4.20978500	2.23673900	-1.54699800
H	-4.33700100	3.07080100	-0.82800700
H	-3.73998200	2.66331900	-2.44250600
N	-5.48653900	1.64906700	-1.95303400
H	-5.91761200	2.25381600	-2.64981800
C	-6.44037000	1.45854200	-0.85876100
H	-6.60098400	2.38874600	-0.27669600
H	-6.02071300	0.72678100	-0.15661900
C	-7.77936800	0.93700500	-1.38244500
H	-8.19164000	1.66150800	-2.10353900
H	-7.62194200	0.00329100	-1.93301700
C	-8.80601900	0.70108600	-0.27267700
H	-8.93160800	1.63192700	0.31190200
H	-8.41676400	-0.05245100	0.42421500
N	-10.05662800	0.17773900	-0.83528900
H	-10.71207100	-0.02894500	-0.08314000
H	-10.50185400	0.89916200	-1.40212500

SI References

1. S. Canneaux, F. Bohr and E. Henon, KiSThelP: A Program to Predict Thermodynamic Properties and Rate Constants from Quantum Chemistry Results, J. Comput. Chem., 2014, 35, 82-93.
2. I. M. Alecu, J. J. Zheng, Y. Zhao and D. G. Truhlar, Computational Thermochemistry: Scale Factor Databases and Scale Factors for Vibrational Frequencies Obtained from Electronic Model Chemistries, J. Chem. Theory Comput., 2010, 6, 2872-2887.
